# A unique genetic code change in the mitochondrial genome of the parasitic nematode *Radopholus similis*

**DOI:** 10.1186/1756-0500-2-192

**Published:** 2009-09-24

**Authors:** Joachim EM Jacob, Bartel Vanholme, Thomas Van Leeuwen, Godelieve Gheysen

**Affiliations:** 1 Department of Molecular Biotechnology, Faculty of Bioscience Engineering, Ghent University, Coupure links 653, 9000 Gent, Belgium; 2 VIB (Flanders Institute for Biotechnology) Department of Plant Systems Biology, Ghent University, Technologiepark 927, 9052 Gent, Belgium; 3 Department of Crop Protection, Faculty of Bioscience Engineering, Ghent University, Coupure links 653, 9000 Gent, Belgium

## Abstract

**Background:**

Mitochondria (mt) contain their own autonomously replicating DNA, constituted as a small circular genome encoding essential subunits of the respiratory chain. Mt DNA is characterized by a genetic code which differs from the standard one. Interestingly, the mt genome of nematodes share some peculiar features, such as small transfer RNAs, truncated ribosomal RNAs and - in the class of Chromadorean nematodes - unidirectional transcription.

**Findings:**

We present the complete mt genomic sequence (16,791 bp) of the plant-parasitic nematode *Radopholus similis *(class Chromadorea). Although it has a gene content similar to most other nematodes, many idiosyncrasies characterize the extremely AT-rich mt genome of *R. similis *(85.4% AT). The secondary structure of the large (16S) rRNA is further reduced, the gene order is unique, the large non-coding region contains two large repeats, and most interestingly, the UAA codon is reassigned from translation termination to tyrosine. In addition, 7 out of 12 protein-coding genes lack a canonical stop codon and analysis of transcriptional data showed the absence of polyadenylation. Northern blot analysis confirmed that only one strand is transcribed and processed. Furthermore, using nucleotide content bias methods, regions for the origin of replication are suggested.

**Conclusion:**

The extraordinary mt genome of *R. similis *with its unique genetic code appears to contain exceptional features correlated to DNA decoding. Therefore the genome may provide an incentive to further elucidate these barely understood processes in nematodes. This comprehension may eventually lead to parasitic nematode-specific control targets as healthy mitochondria are imperative for organism survival. In addition, the presented genome is an interesting exceptional event in genetic code evolution.

## Background

Nematodes are one of the largest phyla of multicellular animals on earth with over 20,000 described species. The burrowing nematode *Radopholus similis *infects numerous (sub)tropical crops and is considered as one of the most damaging pests on banana. Recently transcriptomic sequence data of this parasite were studied and several 'expressed sequence tags' (ESTs) originated from genes of the mitochondrial (mt) genome [1]. Mitochondria are found in all eukaryotic cells and provide the cell with energy through the process of oxidative phosphorylation. Originating from an ancestral endosymbiotic α-proteobacterial species [2], they still contain a haploid, autonomously replicating genome of relatively short length, in nematodes ranging from 12.5 kb to 26 kb [3]. To date, complete mt genomes of 31 nematode species are available in GenBank: 9 of the Enoplean class and 22 (mostly comprising animal-parasitic nematodes) of the Chromadorean class. The mt gene products are usually 2 ribosomal RNAs, 22 transfer RNAs and 12-13 intronless protein-coding genes which encode crucial subunits in respiratory complexes I, III, IV and V. Mt genomes of nematodes differ in some aspects from other metazoan mt genomes. The compact nematode mt genomes usually lack ATPase subunit 8, and contain shortened rRNA molecules and truncated tRNAs. Nearly all nematode mt tRNAs lack one arm, either the TΨC arm which is replaced by the 'TV-replacement loop', or the DHU arm which is replaced by D-replacement loops [3,4]. In addition, in nematodes of the class Chromadorea (containing most important parasitic nematodes), all mt genes are unidirectionally transcribed from one strand.

## Results and discussion

**Figure 1:**
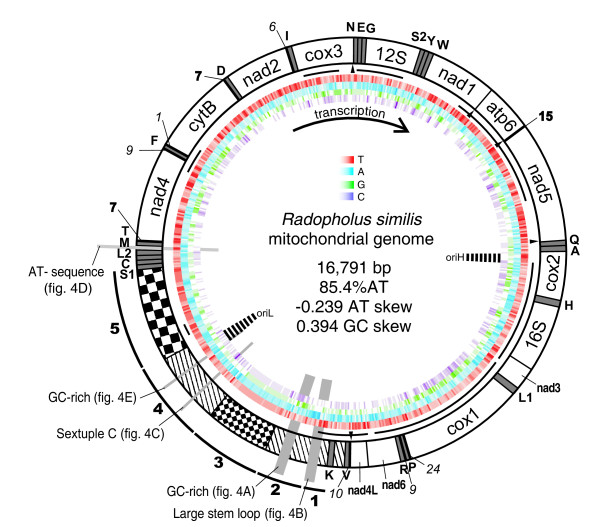
**Overview of the organization of the circular mt DNA of *R. similis***. The arrow indicates direction of transcription. Genes and non-coding regions are indicated: in white, the protein-coding and rRNA genes, in gray, the tRNA genes called by their amino acid symbol (S1: Ser-AGN, S2: Ser-UCN, L1: Leu-CUN, L2: Leu-UUR). Bold and italic numbers indicate non-coding and overlapping nucleotides between neighboring genes, respectively. The pattern-filled part represents the large non-coding region, divided in five regions as explained in the text. The repeat region of 302 bp is filled with large checkers and the 26 bp repeat region is filled with small checkers. The black lines at the inner periphery of the ring represent EST sequences, with UAG stop codons indicated by little black triangles. The colored bar code-like circles represent the nucleotide content of the coding strand, differing intensities corresponding to different content (red: thymine, blue: adenine, green: guanine, purple: cytosine). The positions of secondary structures depicted in figure 4 are indicated by light-gray balks perpendicular to the circle. The predicted origins of replication for the heavy (OriH) and light strand (OriL) are indicated (see figure 5).

The complete mt genome of *R. similis *was amplified in three overlapping fragments, with the complete assembly being 16,791 bp [EMBL:FN313571] (figure 1), which was confirmed by southern blot (additional file 1). With an AT-content of 85.4%, it is the most AT-rich nematode mt genome sequenced to date, and the first complete mt genome of a Chromadorean plant-parasite (hereby disregarding the highly atypical multipartite mitochondrial genome of *Globodera *sp. [5]). All genes are unidirectionally transcribed from the coding strand, which has an asymmetrical nucleotide composition of 52.9% T, 32.5% A, 10.2% G and 4.4% C and is also referred to as the heavy strand. A quarter of the genome is non-coding, comprising two large repeat regions. Similar to other described nematode mt genomes, 22 tRNAs have been predicted ranging from 51 nt to 59 nt in length (additional file 2). All anticodons are conserved in nematodes, except for the UCG anticodon of tRNA^Arg^, which is common in other metazoans, but deviates from the ACG anticodon used by most Chromadorean nematode mt genomes (table 1). Contrary to other nematodes, 2 nucleotides occur instead of one between the DHU and the anticodon arm in some *R. similis *tRNAs. In those tRNA species, the DHU arm is on both sides bordered by uracil (occurring in 8 tRNAs; additional file 2).

**Table 1:** Relative synonymous codon usage (RSCU) and number of codons per 1000 codons (NC1000) in the protein coding genes of the mitochondrial genome of *R. similis*.

**AA**	**codon**	RSCU*	**NC1000**	**AA**	**codon**	**RSCU**	**NC1000**	**AA**	**codon**	**RSCU**	**NC1000**	**AA**	**codon**	**RSCU**	**NC1000**
			
**F**	TTC	0.003	0.295	**S2**	TCA	1.009	8.267	**Y**	TAC	0.000	0.000	**C**	TGC	0.000	0.000
	TTT	1.997	179.805		TCC	0.000	0.000		TAT	1.368	38.382		TGT	2.000	6.200
									
**L2**	TTA	3.436	152.938		TCG	0.108	0.886		TAA	1.632	45.763	**W**	TGA	1.721	10.924
												
	TTG	0.564	25.096		TCT	2.883	23.620	**Stop**	TAG	1.000	1.48		TGG	0.279	1.771
			
**L1**	CTA	0.696	1.181	**P**	CCA	1.231	4.724	**H**	CAC	0.041	0.295	**R**	CGA	0.296	0.590
	CTC	0.000	0.000		CCC	0.000	0.000		CAT	1.959	14.172		CGC	0.000	0.000
												
	CTG	0.174	0.295		CCG	0.154	0.590	**Q**	CAA	1.267	5.610		CGG	0.000	0.000
	CTT	3.130	5.314		CCT	2.615	10.038		CAG	0.733	3.248		CGT	3.704	7.381
			
**I**	ATC	0.006	0.295	**T**	ACA	0.678	2.952	**N**	AAC	0.023	0.590	**S1**	AGA	0.932	10.038
	ATT	1.994	100.384		ACC	0.000	0.000		AAT	1.977	51.668		AGC	0.055	0.590
									
**M**	ATA	1.716	53.440		ACG	0.068	0.295	**K**	AAA	1.448	22.439		AGG	0.137	1.476
	ATG	0.284	8.857		ACT	3.254	14.172		AAG	0.552	8.562		AGT	2.877	31.001
			
**V**	GTA	1.117	14.762	**A**	GCA	0.596	2.067	**D**	GAC	0.000	0.000	**G**	GGA	0.722	7.676
	GTC	0.089	1.181		GCC	0.085	0.295		GAT	2.000	18.010		GGC	0.028	0.295
												
	GTG	0.268	3.543		GCG	0.000	0.000	**E**	GAA	1.460	13.581		GGG	0.167	1.771
	GTT	2.525	33.363		GCT	3.319	11.515		GAG	0.540	5.019		GGT	3.083	32.772

* RSCU is the number of times a particular codon is observed relative to the number of times a codon would be observed in the absence of any codon usage bias. Based on a total of 3378 codons.

**Figure 2:**
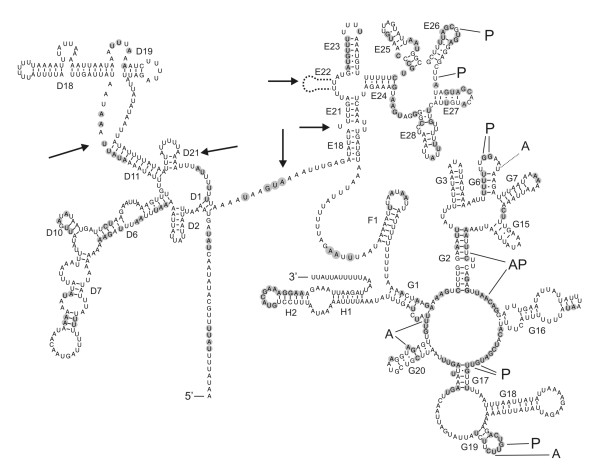
**Predicted secondary structure of the 16S rRNA gene of *R. similis***. Watson-Crick base pairing and G:U base pairing is indicated by a line and a dot, respectively. Numbering of helices is according to De Rijk et al. [29] and other published nematode mt rRNA structures. Binding sites for the amino-acyl of tRNAs (A), peptidyl-transferase (P), or both (AP) as defined by Noller [30] and Hu [6] are indicated. Compared to other nematode 16S rRNAs, shaded nucleotides are conserved in at least 90% of the cases, while arrows indicate remarkable deletions.

Another characteristic feature is the occurrence of three instead of two nucleotides between the amino-acyl acceptor stem and the DHU arm (occurring in 10 tRNAs). Two rRNA genes (12S and 16S) were identified on the mt genome. The 12S rRNA gene (or *rrnS*) is 692 bp long, comparable to other nematode mitochondrial 12S rRNA genes (698 ± 33 bp) (additional file 3). The boundaries of this gene were validated through a CT-RT-PCR technique (additional file 4). The secondary structure of this rRNA revealed similar topology as other published nematode 12S rRNA structures [6-9]. In contrast, the large subunit rRNA (16S rRNA or *rrnL*) is 840 bp in length, which is considerably smaller than the average of 943 ± 57 bp for other nematodes. The reductions in the overall comparable structure compared to other nematodes are indicated by arrows in figure 2. Most conserved nucleotides in nematode mt rRNAs are generally found in loops and not in stems (figure 2 and additional file 3), which is in agreement with observations that interacting proteins often bind to loops [10]. Many nucleotides involved in the P site and A site of the ribosome are conserved; however, conserved residues in the G3 stem, which has been implicated as part of the exit site, are absent [8].

**Figure 3:**
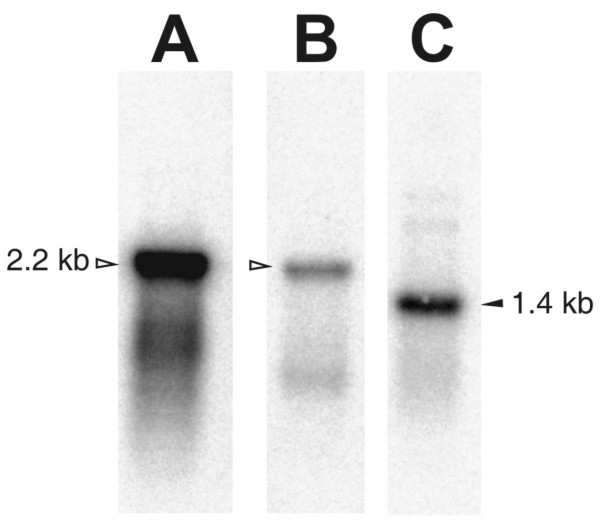
**Northern blot on mt RNA of *R. similis***. A. *cox1 *antisense probe. Hollow arrowhead indicates 2.2 kb. Expected length is 1.6 kb. B. *nad5 *antisense probe. Expected length is 1.5 kb. C. *cytB *antisense probe. Black arrowhead indicates 1.4 kb. Expected length is 1.0 kb. Probing with sense probes gave no detectable signals in all cases (data not shown).

**Figure 4:**
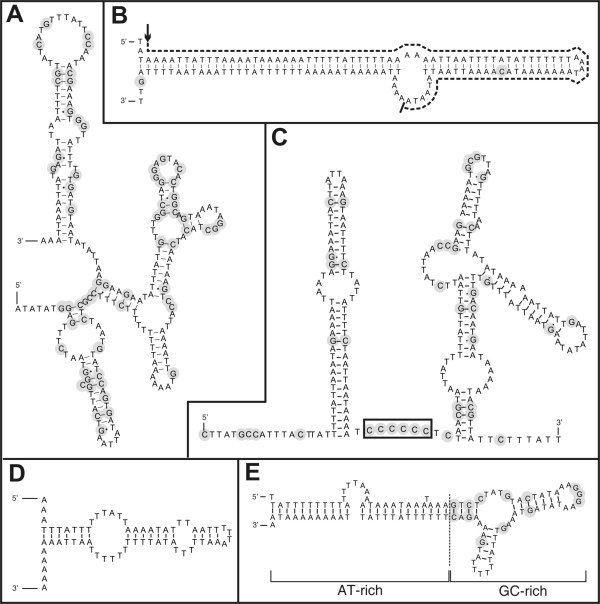
**Summary of potential secondary structures in the non-coding regions**. C and G nucleotides are shaded. A. GC-rich patch located before the short repeat region (9108..9335); B. large stem loop with A-rich 'bulge', with EST evidence indicated by dashed line (arrow: 5' start) (8906..9036); C. The sextuple C motif located in the sequence between the two repeat regions (10498..10729); D. AT-rich non-coding region between tRNA^Met ^and tRNA^Thr ^(12611..12675); E. local GC-rich region, surrounded by AT-rich sequences, located in the sequence between the two repeat regions (10855..10963). A stretch of 8 A nucleotides at the 3' end is similar as described by Hu [6] and Jex [7].

The mt genome contains 12 protein-coding genes (PCGs), lacking - as the majority of nematode mt genomes - the ATPase subunit 8 gene. To our surprise, we discovered that the UAA codon, which encodes translation termination according to the standard invertebrate mitochondrial genetic code, was abundant in all open reading frames of the 12 PCGs. Based on alignments with other nematode mt proteins, UAA encodes for tyrosine in the *R. similis *mt genome [11]. The UAA^Stop ^to UAA^Tyr ^reassignment was confirmed for another *Radopholus *species (*R. arabocoffeae*) based on a sequenced mtDNA fragment (additional file 5). This genetic code change has never been confirmed in any other animal genus. Though a similar codon reassignment was once reported for the planarian *Dugesia japonica *[12], subsequent studies could not confirm this finding [13]. Thus until now, the UAA^Stop ^to UAA^Tyr ^reassignment is unique for the mt genome of *Radopholus*. The sole Stop codon in use, UAG, could only be inferred for 5 PCGs: *nad4L*, *cox3*, *nad1*, *atp6 *and *nad5*. Of the remaining genes, *nad6 *is separated from the subsequent *nad4L *by a single thymine. This is often called a 'truncated stop', as it can form a complete canonical UAA stop codon after polyadenylation [14]. Intriguingly, this could not be the case for *Radopholus *due to the codon reassignment. Absence of stop codons is a very uncommon feature in mitochondrial genes. However, it has been shown that mitochondria of humans, plants and dinoflagellates are capable of translating templates lacking Stop codons [15-17]. The inferred start codons are UUG (2), AUA (5), AUG, AUU (2) and UUA (2), all of which have been reported before in nematode mt genomes. Northern blot using 3 different probes specifically hybridizing to *cytB *(no stop codon), *nad5 *(UAG stop codon) and *cox1 *(no stop codon) revealed that the transcripts of these genes are processed into monocistronic transcripts, although the length appears larger than expected (1.4 kb instead of 1.0, 2.2 instead of 1.5, 2.2 instead of 1.6, for *cytB*, *nad5 *and *cox1*, respectively; see figure 3). This observation can not be explained easily, but it may point to a modification of mRNA molecules that affects their migration in denaturing agarose gels. Probing with the corresponding sense probes revealed no signals (data not shown). We conclude that exclusively the heavy strand is transcribed, eventually producing monocistronic transcripts lacking stop codons as a template for protein synthesis. Investigating EST data, 623 ESTs were aligned to the mt genome. The ESTs cover 7,971 bp (47.5%) of the mt genome. Remarkably, the majority (n = 581) is derived from 12S rRNA, pointing to a higher expression level and/or higher stability compared to other mt genes. None of the mt ESTs contain a poly(A) tail and - in remarkable contrast to the northern blot results - large parts of the mt genome are contiguously covered by EST data, crossing gene borders (figure 1). Only one EST cluster aligned to a gene boundary by starting exactly at the 5' end of tRNA^Glu ^(additional file 6). Attempts to ascertain PCG boundaries using CT-RT-PCR failed. Besides some indels in poly(T) tracts, barely any sequence difference between the mt DNA and ESTs were present - except for 12S rRNA. Three different EST clusters align to this gene, containing 549, 18 and 2 ESTs respectively, with differences of 0, 2.4 and 7.5% respectively. Some transcriptomic data match parts of the non-coding region, often called the control or AT-region in nematode mt genomes. Based on sequence features, the control region in the mt genome of *R. similis *was artificially divided into five sub-regions (figure 1 and 4). Characteristic for the first part are poly-thymine and poly-adenine stretches, resulting in a high AT content of 87.6%. Some of the poly-T stretches are separated by a single G nucleotide, a motif which is found in several other nematode control regions [4,18,19]. A remarkably large and nearly perfect stem loop of 126 bp ends the first region. The second region of 460 bp runs until the small repeat region. This region is enriched in GC nucleotides (81.2% AT), which are concentrated in a 225 bp region and can adapt a stable secondary structure (figure 4A). The third region (84.0% AT) contains a repeat region of 806 bp in which a 26 bp motif is repeated 31 times. The fourth region is an AT-rich sequence part (87.5% AT) of 944 bp with unique features such as a roughly equal G- and C-content and a remarkable stretch of 6 C nucleotides. The sequence surrounding this sextuple C-motif can fold into stable secondary structures (figure 4C). The control region ends with another repeat region of 1,208 bp, containing a 302 bp sequence directly repeated 4 times. This repeat region partly overlaps with the adjacent tRNA^Ser^_AGN_. In contrast to *Caenorhabditis elegans *and *Ascaris suum*, no runs of AT dinucleotides are found in the control region [20]. Fourteen other smaller non-coding regions were found interspersed in between the genes. Although most are small (ranging from 1 to 15 bp), one is 65 bp in length and located between tRNA^Met ^and tRNA^Thr^. This region is solely composed of AT nucleotides and can form a secondary structure (figure 4D).

**Figure 5:**
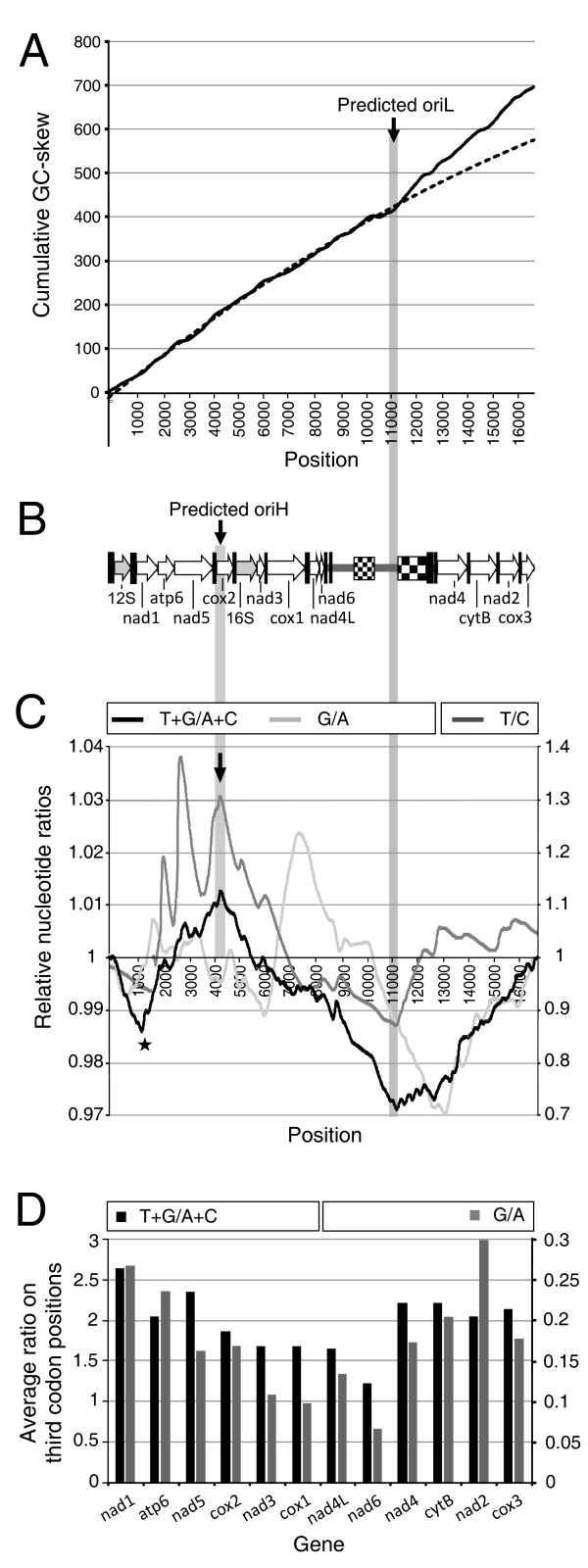
**Nucleotide composition analysis of the mt genome of *R. similis***. A. Based on a cumulative GC-skew (G-C/G+C) graph, the offset (vertical bar) from the fitted model (dashed line) corresponds to the location of the origin of replication of the light strand (OriL) [22]. B. A linear representation of the circular mt genome, with rRNA and PCGs as indicated. Black bars and checkers represent tRNAs and repeat regions, respectively. The predicted OriL is located near the sextuple C motif. The prediction of OriH is explained in C and D. C. Due to deaminations preferably occurring during single-strandness, the ratios T/C (right y-axis), G/A (left y-axis) and T+G/C+A (left y-axis) differ. From these curves, in which 1 is the mean value, predictions about OriL and OriH can be made. The minimum of the T/C curve corresponds to the OriL, while for G/A the minimum is located in the tRNAs preceding *nad4*. These differences could be explained by different kinetics for both deamination processes [21]. The T+G/C+A measure (covering both processes) reaches a maximum at the start of *cox2 *and a minimum at the predicted OriL. From this, two replication origins could be concluded, with OriH located at the start of *cox2*. If OriL is at 11,000 and OriH at 4,000, both replicases meet around 1,000 (assuming similar speeds), leaving this region single-stranded for a short period, causing a local minimum (star). D. The average T+G/A+C (left y-axis) and G/A (right y-axis) of the nucleotides at third codon positions. From *cox2 *on, both measures decrease with a minimum at *nad6*. The G/A ratio shows a pattern corresponding to that of T+G/A+C, as opposed to graph C, where the kinetics of the A to G mutations [21] and the natural variation cause a high level of noise in the G/A ratio.

Mt DNA replication is an asymmetrical process, in which the heavy strand replication initiation precedes that of the light strand. Hence some sequence parts of mt genomes spend more time single stranded, a state in which the DNA is more prone to deaminations of cytosine to uracil, and to a lesser extent of adenine to hypoxanthine [21]. The subsequent accumulation of T and G (at the expense of C and A, respectively) in these regions can provide information about the origins of replication [22]. In this way, the light strand origin of replication is predicted to be located in secondary structures surrounding the sextuple C motif (figure 1 and 5). Analysis of the relative amount of T and G versus A and C, on complete sequence as well as on nucleotides of the third codon positions, showed a pattern that could only be explained by assuming two different origins of replication [21]. Based on these observations, we hypothesize that the origin of the heavy strand is located in the *cox2 *gene (figure 5). Although for nematodes nothing is known on this issue, our data suggest a similar mode of action for mt replication as other Metazoa, with origins of replications correlated to secondary structures in the DNA. The predicted OriL is located at the region harboring the sextuple C motif and surrounding secondary structures, and the predicted OriH lies in the vicinity of two tRNA genes, which could function as replication origins as observed in Vertebrates [23]. However, no clear similarity between the secondary structures reported for other nematodes (see for example [9]) and those of *R. similis *was found.

## Methods

### DNA extraction and LD-PCR

*R. similis *was cultured as decribed in [1]. On 500 ng of total phenol/chloroform extracted DNA from approximately 10,000 nematodes, LD-PCR was performed using a combination of Expand Long Range dNTP Pack (Roche, Mannheim, Germany) and Phusion High-Fidelity DNA Polymerase (Finnzymes, Espoo, Finland) with gene specific primers based on EST sequences [1] (see additional file 6). PCRs were done following the manufacturer's instructions in 50 μL reaction mixtures, containing 6% DMSO. All fragments were directly sequenced.

### Extraction of mitochondria

Approximately 400 μL packed nematodes (approximately 200,000 nematodes) were washed with sterile demineralized water, and brought into 1 mL sucrose buffer (300 mM sucrose, 30 mM Tris, and 10 mM EDTA, pH7.9). With the aid of a Teflon pestle and some sand, a nematode homogenate was obtained and centrifuged for 5 min at 500 g. The supernatant was further purified by additional centrifugation for 5 min at 1000 g. A pellet of mitochondria was obtained by centrifuging the supernatant at 15,000 g for 45 min. Obtained mitochondria were used immediately.

### Mitochondrial RNA extraction

The mitochondria were dissolved in 200 μL Trireagent (Sigma, St. Louis, MO, USA), and incubated for 1 h at RT. The solution was three times sonicated at the lowest setting for 2 sec while keeping on ice (Branson Sonifier 250, Danbury, USA). After incubation for 15 min at room temperature, 40 μL chloroform was added and the tube was vigorously shaken by hand. Further RNA extraction steps were performed following the manufacturer's instructions.

### CT-RT-PCR

Mt RNA was circularized using T4 RNA ligase (Fermentas, St. Leon-Rot, Germany), using 1 μg mt RNA in a total volume of 20 μL, following the manufacturer's instructions. After incubation for 1 h at 37°C, the volume was adjusted to 200 μL with demineralized water, and RNA purified by standard phenol/chloroform extraction. After resuspension in 20 μL demineralized water, the circularized RNA was used for the reverse transcriptase reaction using SuperScript II (Invitrogen, Carlsbad, CA, USA) following manufacturer's instructions and a reverse-oriented gene-specific primer located at the 5' end of the gene (see additional file 4 and 7). The resulting cDNA was subjected to nested PCR using gene-specific primers. PCR products were ligated into the pGEM-T vector (Promega, Wisconsin, USA) and transformed into *E. coli *DH5α cells. Positive clones were selected on LB plates supplemented with carbenicilin (100 μg/ml) and inserts were sequenced.

### Northern blot

Mt RNA was separated on a 1× MOPS 1% formaldehyde agarose gel and blotted (downward capillary) onto Hybond-N+ membrane (Amersham, Uppsala, Sweden). RNA probes were generated from plasmids containing fragments of *cox1*, *cytB *and *nad5 *as template (see additional file 7 for primers) using Riboprobe kit (Promega) in presence of ^32^P-labeled nucleotides. Hybridization was done overnight at 56°C, and after washing, signals were visualized using a fluor imager FLA-5100 (Fujifilm, Tokyo, Japan).

### Southern blot

1.2 μg total DNA was digested with SpeI and XmnI restriction enzymes, and separated by inversed field electrophoresis in 0.5 × TBE 1% agarose gel. After capillary blot, hybridization was performed using the High Prime DIG labeling Starter Kit (Roche) following manufacturer's instructions with a probe constructed using primers RsNADHD_2 and RsCOXI_7 (see additional file 7) covering 3,504 bp of the mitochondrial genome. The chemiluminescent signal was detected on a fluor imager FLA-5100 using standard settings (Fujifilm).

### Annotation and nucleotide composition analysis

Combinations of BLAST searches and ClustalW alignments to other nematode mt genes (collected using MitoBank2.1 [24]) were used to detect protein gene and rRNA sequences. Folding of rRNA sequences based on published structures was done with the aid of Mfold [25], and structures were edited with RNAviz [26]. Predictions of the tRNA genes by tRNAScan-SE [27], set to detect mt nematode tRNAs, were confirmed by aligning to other nematode mt tRNAs using LARA [28]. Analysis of nucleotide composition was performed with in-house perl programs. Based on Grigoriev to estimate the OriL [22], GC-skew values (G-C/G+C) were calculated for each 5^th ^position in the genome using a window of 300 nt and the cumulative values plotted. The graph followed a second order polynomial function (R^2 ^> 0.999), except for an offset starting around position 11,100, which points to OriL. Due to deaminations occurring during on single-stranded states caused by replication, T/C and G/A ratios increase along mt genomes. Based on a sliding window of 244 nt (depending on C content), the T/C, G/A and T+G/C+A was calculated for every 10^th ^position. The cumulative values followed a linear function (R^2^>0.999). The relative difference with this function is plotted, where 1 corresponds to the mean increase over the genome. Values higher than one represent a higher than average amount of T, G, or T+G compared to C, A, or C+A respectively. In addition, for each gene, the different ratios were determined based on nucleotides on the third codon positions. In this way, T/C could not be calculated for 7 genes (*cox2*, *cytB*, *nad2*, *nad4*, *nad4L *and *nad6*) due to the lack of C. Therefore, only T+G/C+A and G/A is plotted in figure 5D.

## Competing interests

The authors declare that they have no competing interests.

## Authors' contributions

JJ carried out the experiments and drafted the manuscript. BV, TVL and LG participated in discussion of the results, design and drafting of the manuscript. All authors read and approved the final manuscript.

## Supplementary Material

Additional file 1 **Southern blot on digested *R. similis *mt DNA.** Lane 1 and 2 are digested with the single-cutters SpeI and XmnI respectively. Lane 3 is digested with both enzymes. Expected lengths are 16.8 kb for lane 1 and lane2 and 5.4 kb for lane 3. For information of the used probe, see 'Methods' section in the text, and additional file 7. Click here for file

Additional file 2 **Secondary structures predicted for the 22 tRNA genes of the *R. similis *mitochondrial genome.** In the downward right corner, the general tRNA structure is depicted with indication of the different stem-loops and features. Click here for file

Additional file 3 **Predicted secondary structure of the mt 12S rRNA (rrnS) gene of *R. similis*. Watson-Crick base pairing is indicated by a line, whereas a G:U base pairing is indicated by a dot**. Proposed tertiary interactions are represented by long, straight lines. Numbers at stems identify the conserved secondary structure elements. Shaded nucleotides are conserved in at least 90% of the currently available nematode mt 12S rRNA sequences. Click here for file

Additional file 4 **A. Schematic representation of the 'circularization-reverse transcriptase-polymerase chain reaction' (CT-RT-PCR) approach to determine the 5' and 3' ends of an RNA molecule.** B. alignment of the results from two independent 12S rRNA gene experiments. For ease, the sequence of the insert of the two clones are split at the 5'-3' junction, and called *-5pr and *-3pr in the alignment. The drawing above the alignment corresponds to the scheme depicted under A. Click here for file

Additional file 5 **The sequence (normal and reverse complement, first two lines) of a cloned fragment of the mitochondrial genome of *R. arabocoffeae *aligned to the corresponding region of *R. similis*.** The genes are indicated above the alignment with the gene boundary indicated by a vertical black bar. TAA codons are boxed and indicated by stars. Click here for file

Additional file 6 **Alignment of all EST data to the mitochondrial genome. The first line is a sequence part of the mitochondrial genome of *R. similis*. **The subsequent lines starting with 'rs' indicate mitochondrial gene sequences. The other sequences are mitochondrial EST sequences, indicated by clone name or gi number. Only the EST sequences derived from 12S rRNA are represented as clusters since they contain too many sequences to be nicely presented in this alignment. Click here for file

Additional file 7 **Listing of the used primer sequences for amplifying the genome, the CT-RT-PCR and the northern blot.** Click here for file
