# Supplementary figures and images for: A unique genetic code change in the mitochondrial genome of the parasitic nematode Radopholus similis
Source: BMC Res Notes. 2009 Sep 24;2:192. doi: 10.1186/1756-0500-2-192 (PMC2761399; doi:10.1186/1756-0500-2-192)

# Additional file 1

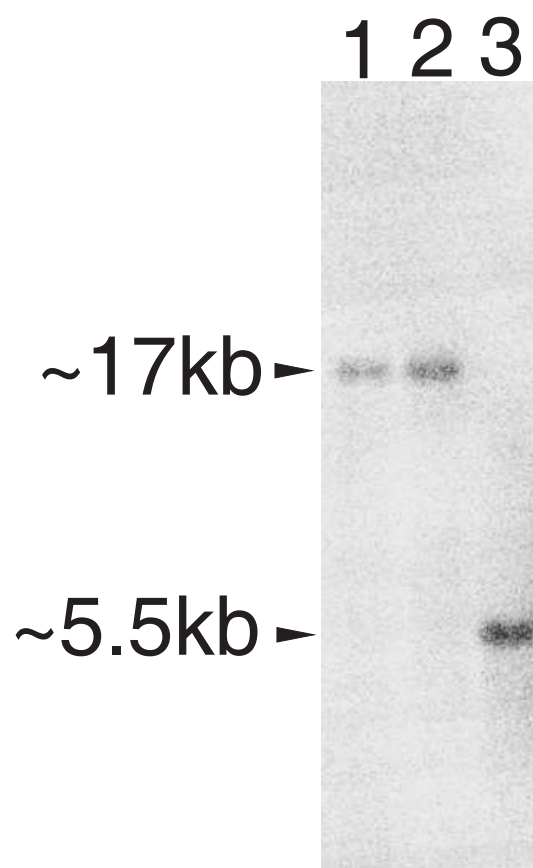

Supplement: Additional file 1 — Southern blot on digested R. similis mt DNA. Lane 1 and 2 are digested with the single-cutters SpeI and XmnI respectively. Lane 3 is digested with both enzymes. Expected lengths are 16.8 kb for lane 1 and lane2 and 5.4 kb for lane 3. For information of the used probe, see 'Methods' section in the text, and additional file 7. [file 1756-0500-2-192-S1.PDF]

# Additional file 2

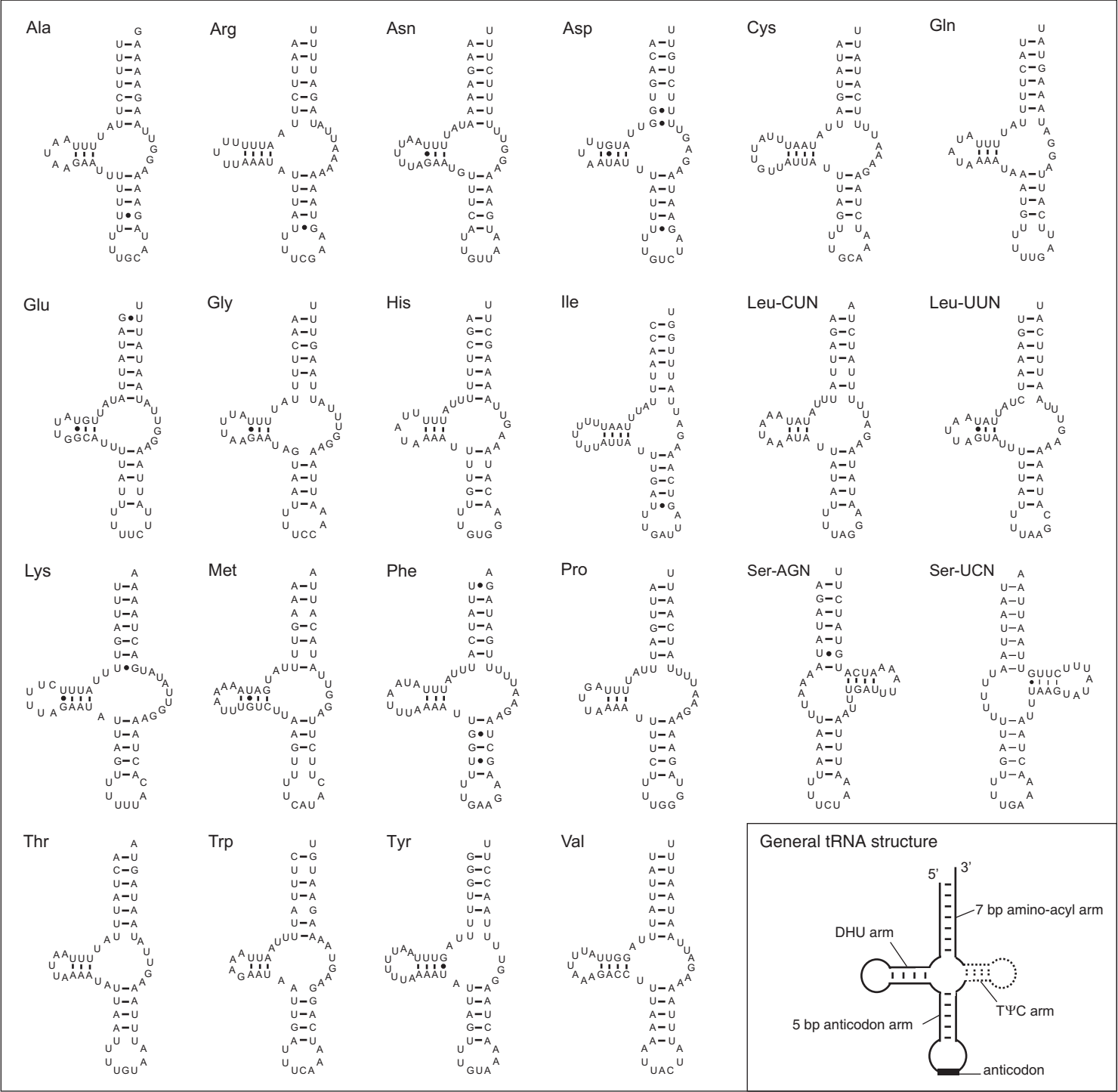

Supplement: Additional file 2 — Secondary structures predicted for the 22 tRNA genes of the R. similis mitochondrial genome. In the downward right corner, the general tRNA structure is depicted with indication of the different stem-loops and features. [file 1756-0500-2-192-S2.PDF]

# Additional file 3

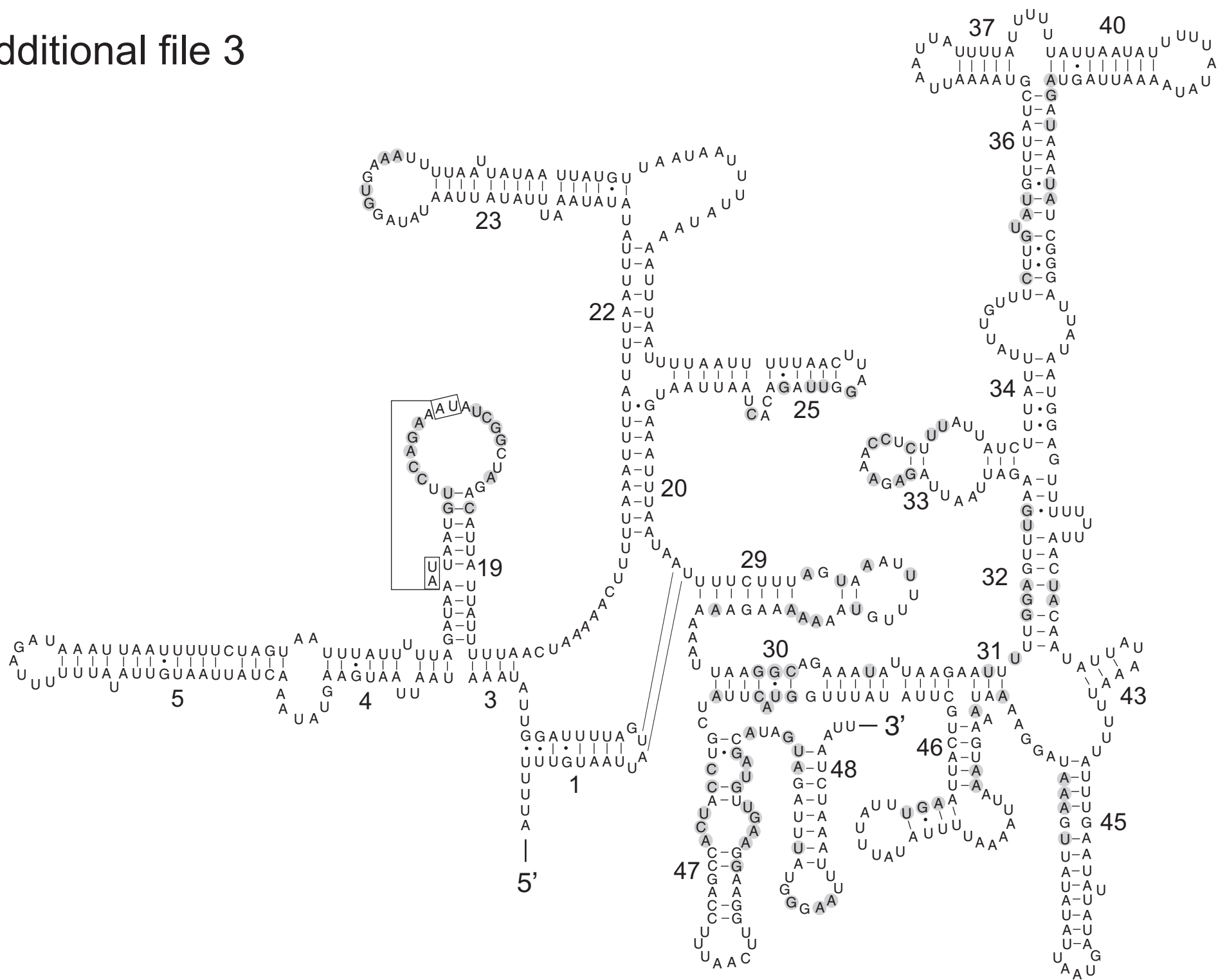

Supplement: Additional file 3 — Predicted secondary structure of the mt 12S rRNA (rrnS) gene of R. similis. Watson-Crick base pairing is indicated by a line, whereas a G:U base pairing is indicated by a dot. Proposed tertiary interactions are represented by long, straight lines. Numbers at stems identify the conserved secondary structure elements. Shaded nucleotides are conserved in at least 90% of the currently available nematode mt 12S rRNA sequences. [file 1756-0500-2-192-S3.PDF]
