# Supplementary material for: A unique genetic code change in the mitochondrial genome of the parasitic nematode Radopholus similis
Source: BMC Res Notes. 2009 Sep 24;2:192. doi: 10.1186/1756-0500-2-192 (PMC2761399; doi:10.1186/1756-0500-2-192)

Additional file 4

A. Schematic representation of the CT-RT-PCR technique

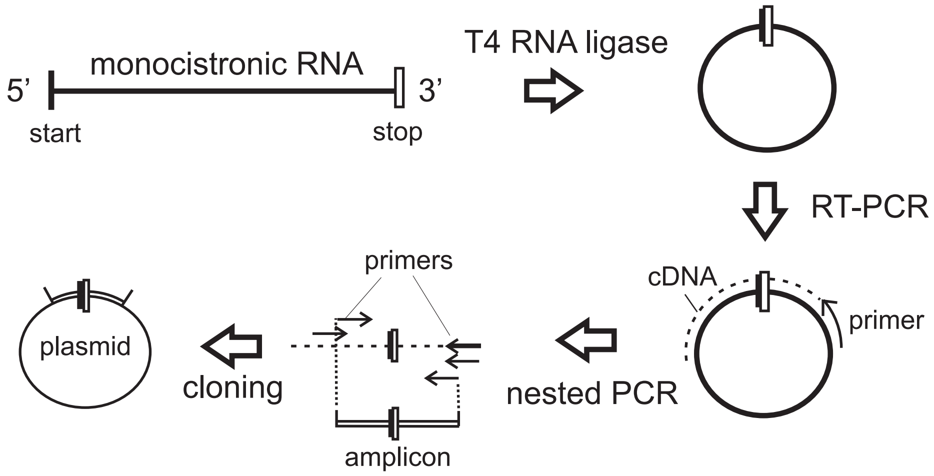

B. Results of the CT-RT-PCR of 12S rRNA of *Radopholus similis*

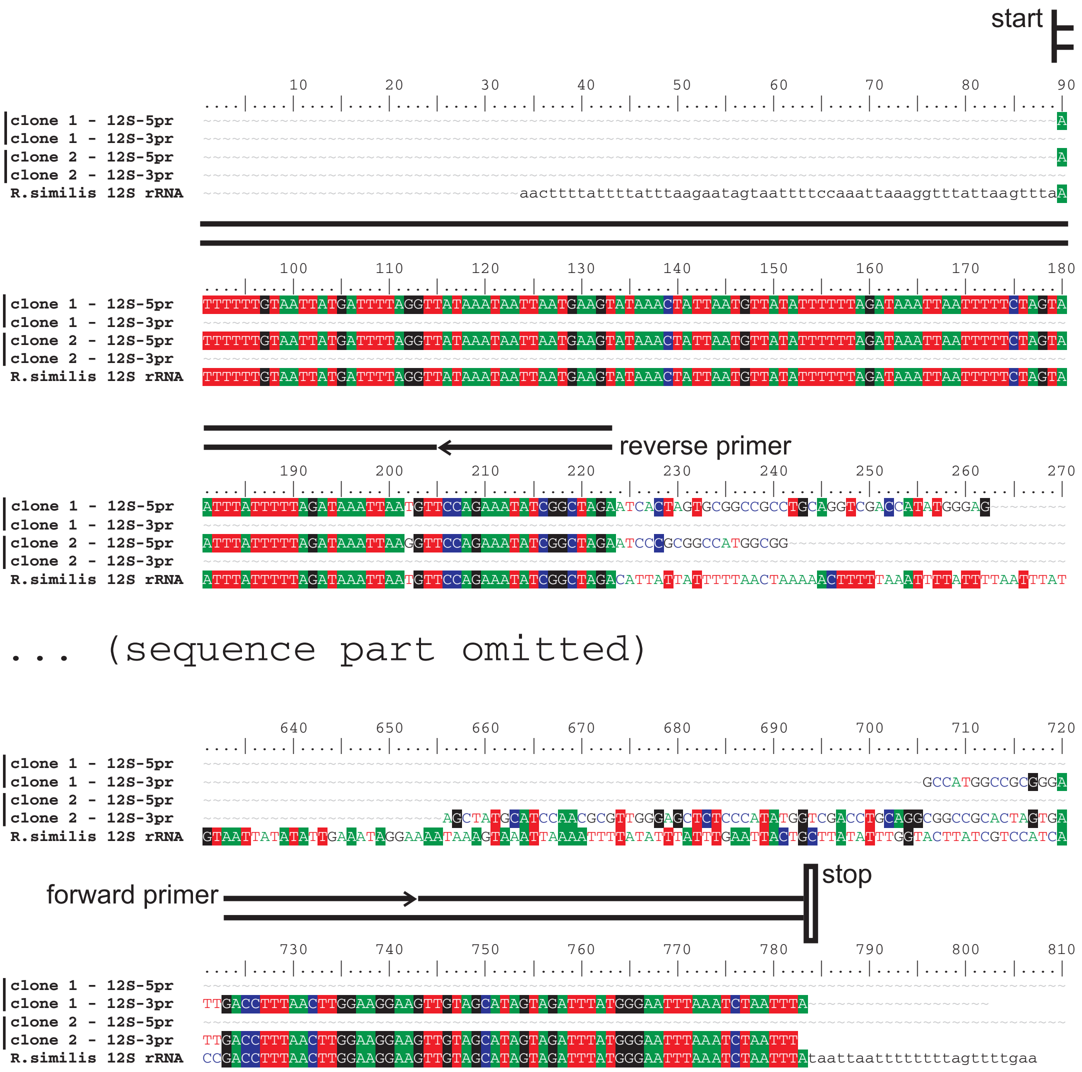

Supplement: Additional file 4 — A. Schematic representation of the 'circularization-reverse transcriptase-polymerase chain reaction' (CT-RT-PCR) approach to determine the 5' and 3' ends of an RNA molecule. B. alignment of the results from two independent 12S rRNA gene experiments. For ease, the sequence of the insert of the two clones are split at the 5'-3' junction, and called *-5pr and *-3pr in the alignment. The drawing above the alignment corresponds to the scheme depicted under A. [file 1756-0500-2-192-S4.PDF]
