# Supplementary material for: A unique genetic code change in the mitochondrial genome of the parasitic nematode Radopholus similis
Source: BMC Res Notes. 2009 Sep 24;2:192. doi: 10.1186/1756-0500-2-192 (PMC2761399; doi:10.1186/1756-0500-2-192)

Additional file 5

Legend

vietPop: *Radopholus arabocoffeae* population originating from Vietnam.
uganPop: *Radopholus similis* population originating from Uganda.

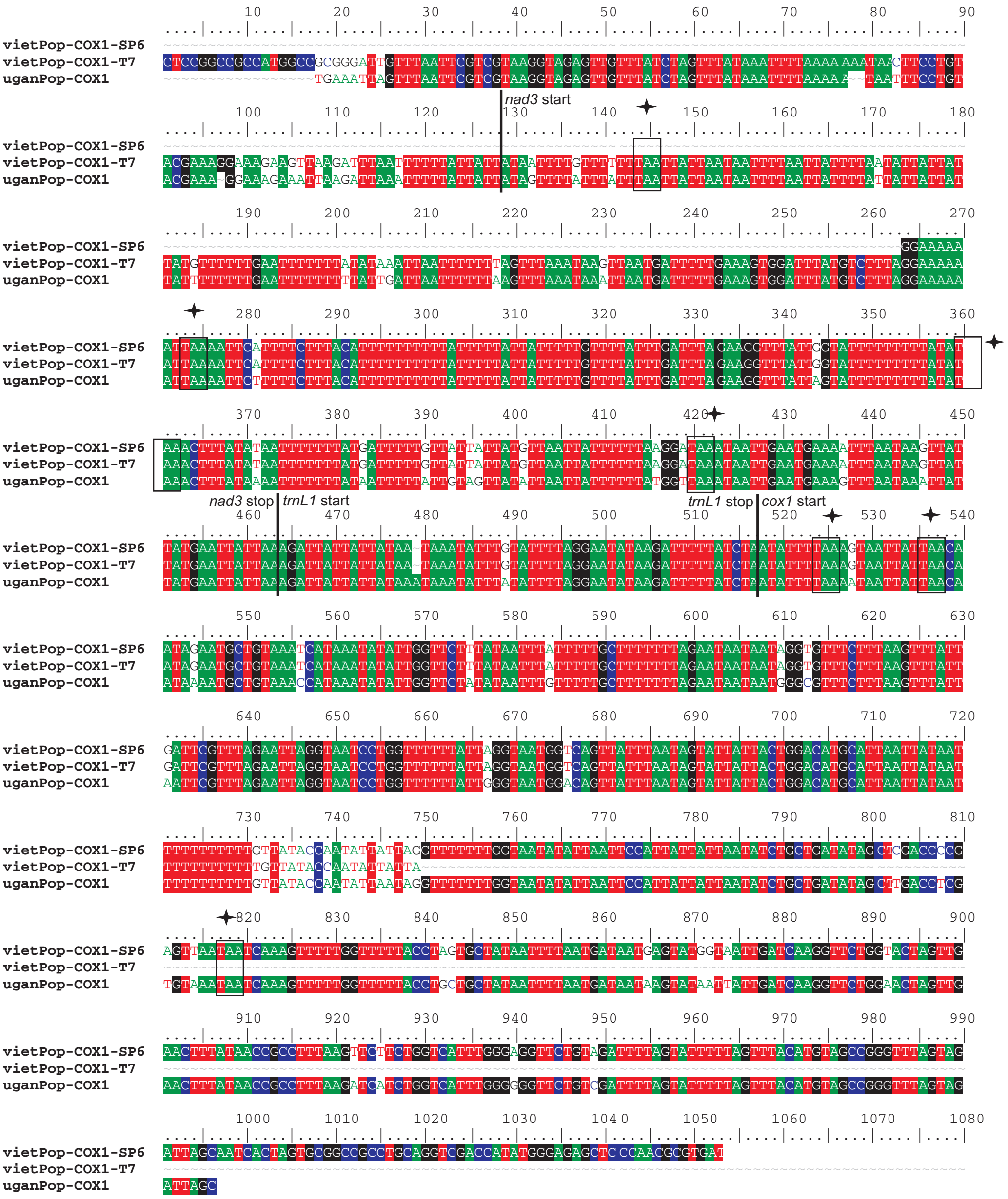

Supplement: Additional file 5 — The sequence (normal and reverse complement, first two lines) of a cloned fragment of the mitochondrial genome of R. arabocoffeae aligned to the corresponding region of R. similis. The genes are indicated above the alignment with the gene boundary indicated by a vertical black bar. TAA codons are boxed and indicated by stars. [file 1756-0500-2-192-S5.PDF]
