# Supplementary material for: A unique genetic code change in the mitochondrial genome of the parasitic nematode Radopholus similis
Source: BMC Res Notes. 2009 Sep 24;2:192. doi: 10.1186/1756-0500-2-192 (PMC2761399; doi:10.1186/1756-0500-2-192)
Supplement: Additional file 6 — Alignment of all EST data to the mitochondrial genome. The first line is a sequence part of the mitochondrial genome of R. similis. The subsequent lines starting with 'rs' indicate mitochondrial gene sequences. The other sequences are mitochondrial EST sequences, indicated by clone name or gi number. Only the EST sequences derived from 12S rRNA are represented as clusters since they contain too many sequences to be nicely presented in this alignment. [file 1756-0500-2-192-S6.PDF]

Additional file 6

ESTs matching from *trnE* to *rrnS*

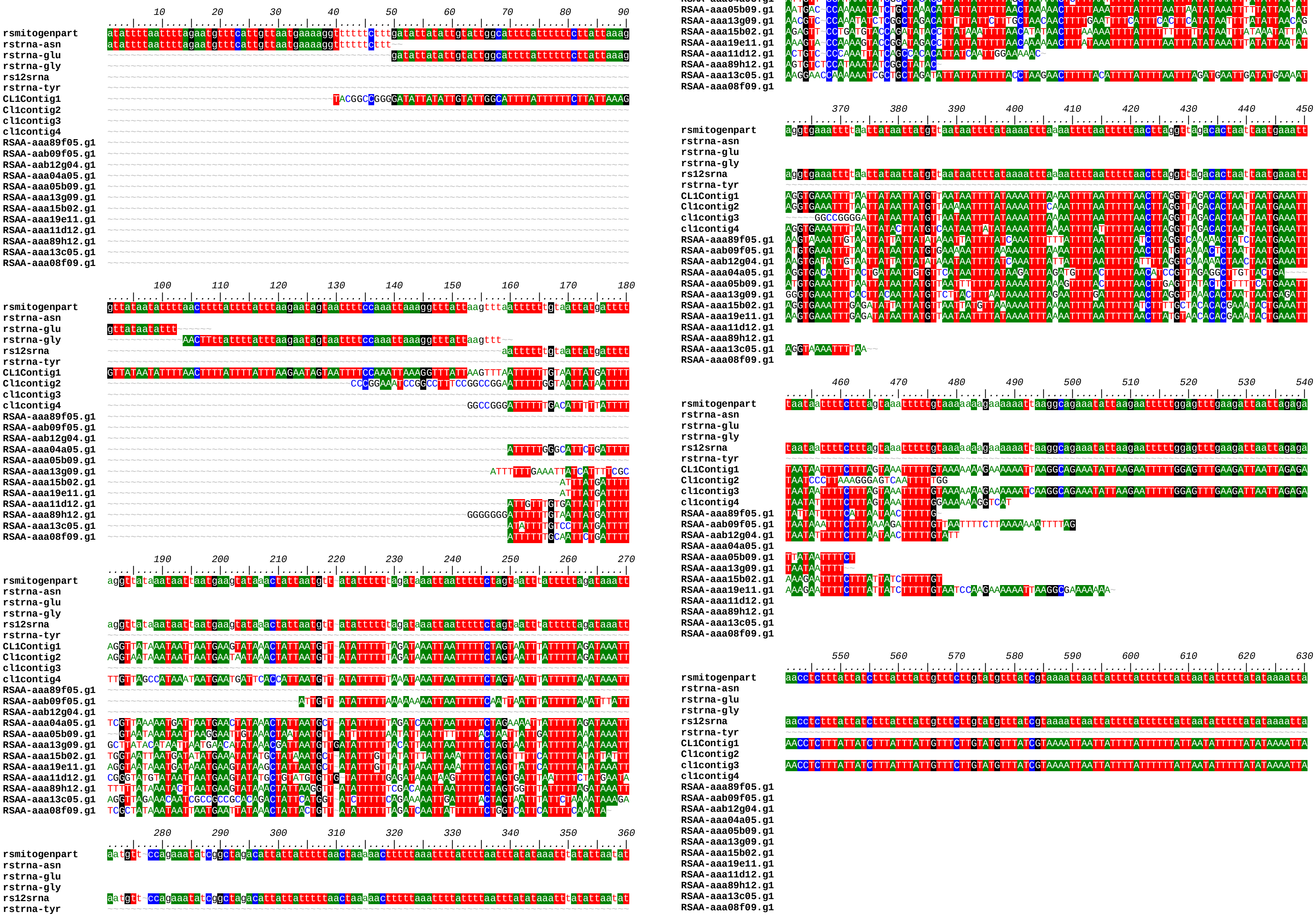

```
                      640       650       660       670       680       690       700       710       720
                  ....|....|....|....|....|....|....|....|....|....|....|....|....|....|....|....|....|....|
rsmitogenpart     gtagataaatatcgggattataatggagttttttttaactacaatattataaatttttatttgaatattatagtaattatatattgaaat
rstrna-asn
rstrna-glu
rstrna-gly
rs12srna          gtagataaatatcgggattataatggagttttttttaactacaatattataaatttttatttgaatattatagtaattatatattgaaat
rstrna-tyr        ~~~~~~~~~~~~~~~~~~~~~~~~~~~~~~~~~~~~~~~~~~~~~~~~~~~~~~~~~~~~~~~~~~~~~~~~~~~~~~~~~~~~~~~~~~
CL1Contig1        GTAGATAAATATCGGGATTATAATGGAGTTTTTTTTAACTACAATATTATAAATTTTTATTTGAATATTATAGTAATTATATATTGAAAT
Cl1contig2
cl1contig3        GTAGATAAATATCGGGGACACTAATTAATGAAATTTAATAATTTTCTTTAGTAAATTTTTGGGA
cl1contig4
RSAA-aaa89f05.g1
RSAA-aab09f05.g1
RSAA-aab12g04.g1
RSAA-aaa04a05.g1
RSAA-aaa05b09.g1
RSAA-aaa13g09.g1
RSAA-aaa15b02.g1
RSAA-aaa19e11.g1
RSAA-aaa11d12.g1
RSAA-aaa89h12.g1
RSAA-aaa13c05.g1
RSAA-aaa08f09.g1

                      730       740       750       760       770       780       790       800       810
                  ....|....|....|....|....|....|....|....|....|....|....|....|....|....|....|....|....|....|
rsmitogenpart     aggaaaataaagtaaattaaaattttatatttatttgaattactgcttatatttggtacttatcgtccatcaccgacctttaacttggaa
rstrna-asn
rstrna-glu
rstrna-gly
rs12srna          aggaaaataaagtaaattaaaattttatatttatttgaattactgcttatatttggtacttatcgtccatcaccgacctttaacttggaa
rstrna-tyr        ~~~~~~~~~~~~~~~~~~~~~~~~~~~~~~~~~~~~~~~~~~~~~~~~~~~~~~~~~~~~~~~~~~~~~~~~~~~~~~~~~~~~~~~~~~
CL1Contig1        AGGAA
Cl1contig2
cl1contig3
cl1contig4
RSAA-aaa89f05.g1
RSAA-aab09f05.g1
RSAA-aab12g04.g1
RSAA-aaa04a05.g1
RSAA-aaa05b09.g1
RSAA-aaa13g09.g1
RSAA-aaa15b02.g1
RSAA-aaa19e11.g1
RSAA-aaa11d12.g1
RSAA-aaa89h12.g1
RSAA-aaa13c05.g1
RSAA-aaa08f09.g1

                      820       830       840       850       860       870       880       890       900
                  ....|....|....|....|....|....|....|....|....|....|....|....|....|....|....|....|....|....|
rsmitogenpart     ggaagttgtagcatagtagatttatgggaatttaaatctaatttaattaattttttttagttttgaaaactaatttaagtatatttcttg
rstrna-asn
rstrna-glu
rstrna-gly
rs12srna          ggaagttgtagcatagtag
rstrna-tyr        ~~~~~~~~~~~~~~~~~~~~~~~~~~~~~~~~~~~~~~~~~~~~~~~~~~~~~~~~~~~~~~~~~~~~~~~~~~~~~~~~~~~~~~~~~~
CL1Contig1
Cl1contig2
cl1contig3
cl1contig4
RSAA-aaa89f05.g1
RSAA-aab09f05.g1
RSAA-aab12g04.g1
RSAA-aaa04a05.g1
RSAA-aaa05b09.g1
RSAA-aaa13g09.g1
RSAA-aaa15b02.g1
RSAA-aaa19e11.g1
RSAA-aaa11d12.g1
RSAA-aaa89h12.g1
RSAA-aaa13c05.g1
RSAA-aaa08f09.g1

                      910       920       930       940       950       960
                  ....|....|....|....|....|....|....|....|....|....|....|....|....
rsmitogenpart     ttaattaagggtttttagtttaattttttaaaatattagtttgtaaaactaaggttttttaacctt
rstrna-asn
rstrna-glu
rstrna-gly
rs12srna
rstrna-tyr        ~~~~~~~~gggtttttagtttaattttttaaaatattagtttgtaaaactaaggttttttaacctt
CL1Contig1
Cl1contig2
cl1contig3
cl1contig4
RSAA-aaa89f05.g1
RSAA-aab09f05.g1
RSAA-aab12g04.g1
RSAA-aaa04a05.g1
RSAA-aaa05b09.g1
RSAA-aaa13g09.g1
RSAA-aaa15b02.g1
RSAA-aaa19e11.g1
RSAA-aaa11d12.g1
RSAA-aaa89h12.g1
RSAA-aaa13c05.g1
RSAA-aaa08f09.g1
```

EST matching from *nad1* to *nad5*

```
                         10        20        30        40        50        60        70        80        90
                     ....|....|....|....|....|....|....|....|....|....|....|....|....|....|....|....|....|....|
rsmitogenpart        atattattatttatatttttttttaatagttttaattttgttaagtttagcatttttaactttaagtgaacattttcttttagctttaagt
rsnad1               atattattatttatatttttttttaatagttttaattttgttaagtttagcatttttaactttaagtgaacattttcttttagctttaagt
rsatp6               ~~~~~~~~~~~~~~~~~~~~~~~~~~~~~~~~~~~~~~~~~~~~~~~~~~~~~~~~~~~~~~~~~~~~~~~~~~~~~~~~~~~~~~~~~~
rsnad5               ~~~~~~~~~~~~~~~~~~~~~~~~~~~~~~~~~~~~~~~~~~~~~~~~~~~~~~~~~~~~~~~~~~~~~~~~~~~~~~~~~~~~~~~~~~
gi|159496584|gb|EY   ~~~~~~~~~~~~~~~~~~~~~~~~~~~~~~~~~~~~~~~~~~~~~~~~~~~~~~~~~~~~~~~~~~~~~~~~~~~~~~~~~~~~~~~~~~
gi|159496970|gb|EY   ~~~~~~~~~~~~~~~~~~~~~~~~~~~~~~~~~~~~~~~~~~~~~~~~~~~~~~~~~~~~~~~~~~~~~~~~~~~~~~~~~~~~~~~~~~
gi|159493588|gb|EY   ~~~~~~~~~~~~~~~~~~~~~~~~~~~~~~~~~~~~~~~~~~~~~~~~~~~~~~~~~~~~~~~~~~~~~~~~~~~~~~~~~~~~~~~~~~
gi|159493287|gb|EY   ~~~~~~~~~~~~~~~~~~~~~~~~~~~~~~~~~~~~~~~~~~~~~~~~~~~~~~~~~~~~~~~~~~~~~~~~~~~~~~~~~~~~~~~~~~
gi|159495054|gb|EY   ~~~~~~~~~~~~~~~~~~~~~~~~~~~~~~~~~~~~~~~~~~~~~~~~~~~~~~~~~~~~~~~~~~~~~~~~~~~~~~~~~~~~~~~~~~
gi|159493125|gb|EY   ~~~~~~~~~~~~~~~~~~~~~~~~~~~~~~~~~~~~~~~~~~~~~~~~~~~~~~~~~~~~~~~~~~~~~~~~~~~~~~~~~~~~~~~~~~
gi|159493667|gb|EY   ~~~~~~~~~~~~~~~~~~~~~~~~~~~~~~~~~~~~~~~~~~~~~~~~~~~~~~~~~~~~~~~~~~~~~~~~~~~~~~~~~~~~~~~~~~
gi|159498247|gb|EY   ~~~~~~~~~~~~~~~~~~~~~~~~~~~~~~~~~~~~~~~~~~~~~~~~~~~~~~~~~~~~~~~~~~~~~~~~~~~~~~~~~~~~~~~~~~

                        100       110       120       130       140       150       160       170       180
                     ....|....|....|....|....|....|....|....|....|....|....|....|....|....|....|....|....|....|
rsmitogenpart        cagaatcgtctttcacctaataaaagacttttttaagggttatttcaagctttggttgatggtttaaagttattgcaaaaagaataagtg
rsnad1               cagaatcgtctttcacctaataaaagacttttttaagggttatttcaagctttggttgatggtttaaagttattgcaaaaagaataagtg
rsatp6               ~~~~~~~~~~~~~~~~~~~~~~~~~~~~~~~~~~~~~~~~~~~~~~~~~~~~~~~~~~~~~~~~~~~~~~~~~~~~~~~~~~~~~~~~~~
rsnad5               ~~~~~~~~~~~~~~~~~~~~~~~~~~~~~~~~~~~~~~~~~~~~~~~~~~~~~~~~~~~~~~~~~~~~~~~~~~~~~~~~~~~~~~~~~~
gi|159496584|gb|EY   ~~~~~~~~~~~~~~~~~~~~~~~~~~~~~~~~~~~~~~~~~~~~~~~~~~~~~~~~~~~~~~~~~~~~~~~~~~~~~~~~~~~~~~~~~~
gi|159496970|gb|EY   ~~~~~~~~~~~~~~~~~~~~~~~~~~~~~~~~~~~~~~~~~~~~~~~~~~~~~~~~~~~~~~~~~~~~~~~~~~~~~~~~~~~~~~~~~~
gi|159493588|gb|EY   ~~~~~~~~~~~~~~~~~~~~~~~~~~~~~~~~~~~~~~~~~~~~~~~~~~~~~~~~~~~~~~~~~~~~~~~~~~~~~~~~~~~~~~~~~~
gi|159493287|gb|EY   ~~~~~~~~~~~~~~~~~~~~~~~~~~~~~~~~~~~~~~~~~~~~~~~~~~~~~~~~~~~~~~~~~~~~~~~~~~~~~~~~~~~~~~~~~~
gi|159495054|gb|EY   ~~~~~~~~~~~~~~~~~~~~~~~~~~~~~~~~~~~~~~~~~~~~~~~~~~~~~~~~~~~~~~~~~~~~~~~~~~~~~~~~~~~~~~~~~~
gi|159493125|gb|EY   ~~~~~~~~~~~~~~~~~~~~~~~~~~~~~~~~~~~~~~~~~~~~~~~~~~~~~~~~~~~~~~~~~~~~~~~~~~~~~~~~~~~~~~~~~~
gi|159493667|gb|EY   ~~~~~~~~~~~~~~~~~~~~~~~~~~~~~~~~~~~~~~~~~~~~~~~~~~~~~~~~~~~~~~~~~~~~~~~~~~~~~~~~~~~~~~~~~~
gi|159498247|gb|EY   ~~~~~~~~~~~~~~~~~~~~~~~~~~~~~~~~~~~~~~~~~~~~~~~~~~~~~~~~~~~~~~~~~~~~~~~~~~~~~~~~~~~~~~~~~~

                        190       200       210       220       230       240       250       260       270
                     ....|....|....|....|....|....|....|....|....|....|....|....|....|....|....|....|....|....|
rsmitogenpart        ttagtagagaagataatatttattaattattatttattaacttttattaatttttctttaatattaattttgttttttattattccttat
rsnad1               ttagtagagaagataatatttattaattattatttattaacttttattaatttttctttaatattaattttgttttttattattccttat
rsatp6               ~~~~~~~~~~~~~~~~~~~~~~~~~~~~~~~~~~~~~~~~~~~~~~~~~~~~~~~~~~~~~~~~~~~~~~~~~~~~~~~~~~~~~~~~~~
rsnad5               ~~~~~~~~~~~~~~~~~~~~~~~~~~~~~~~~~~~~~~~~~~~~~~~~~~~~~~~~~~~~~~~~~~~~~~~~~~~~~~~~~~~~~~~~~~
gi|159496584|gb|EY   ~~~~~~~~~~~~~~~~~~~~~~~~~~~~~~~~~~~~~~~~~~~~~~~~~~~~~~~~~~~~~~~~~~~~~~~~~~~~~~~~~~~~~~~~~~
gi|159496970|gb|EY   ~~~~~~~~~~~~~~~~~~~~~~~~~~~~~~~~~~~~~~~~~~~~~~~~~~~~~~~~~~~~~~~~~~~~~~~~~~~~~~~~~~~~~~~~~~
gi|159493588|gb|EY   ~~~~~~~~~~~~~~~~~~~~~~~~~~~~~~~~~~~~~~~~~~~~~~~~~~~~~~~~~~~~~~~~~~~~~~~~~~~~~~~~~~~~~~~~~~
gi|159493287|gb|EY   ~~~~~~~~~~~~~~~~~~~~~~~~~~~~~~~~~~~~~~~~~~~~~~~~~~~~~~~~~~~~~~~~~~~~~~~~~~~~~~~~~~~~~~~~~~
gi|159495054|gb|EY   ~~~~~~~~~~~~~~~~~~~~~~~~~~~~~~~~~~~~~~~~~~~~~~~~~~~~~~~~~~~~~~~~~~~~~~~~~~~~~~~~~~~~~~~~~~
gi|159493125|gb|EY   ~~~~~~~~~~~~~~~~~~~~~~~~~~~~~~~~~~~~~~~~~~~~~~~~~~~~~~~~~~~~~~~~~~~~~~~~~~~~~~~~~~~~~~~~~~
gi|159493667|gb|EY   ~~~~~~~~~~~~~~~~~~~~~~~~~~~~~~~~~~~~~~~~~~~~~~~~~~~~~~~~~~~~~~~~~~~~~~~~~~~~~~~~~~~~~~~~~~
gi|159498247|gb|EY   ~~~~~~~~~~~~~~~~~~~~~~~~~~~~~~~~~~~~~~~~~~~~~~~~~~~~~~~~~~~~~~~~~~~~~~~~~~~~~~~~~~~~~~~~~~

                        280       290       300       310       320       330       340       350       360
                     ....|....|....|....|....|....|....|....|....|....|....|....|....|....|....|....|....|....|
rsmitogenpart        ttttatttttttagtatgaagttgaattttttttttttattatttattattggtttaagtgtttatttttttattgtagtaagttaattt
rsnad1               ttttatttttttagtatgaagttgaattttttttttttattatttattattggtttaagtgtttatttttttattgtagtaagttaattt
rsatp6               ~~~~~~~~~~~~~~~~~~~~~~~~~~~~~~~~~~~~~~~~~~~~~~~~~~~~~~~~~~~~~~~~~~~~~~~~~~~~~~~~~~~~~~~~~~
rsnad5               ~~~~~~~~~~~~~~~~~~~~~~~~~~~~~~~~~~~~~~~~~~~~~~~~~~~~~~~~~~~~~~~~~~~~~~~~~~~~~~~~~~~~~~~~~~
gi|159496584|gb|EY   ~~~~~~~~~~~~~~~~~~~~~~~~~~~~~~~~~~~~~~~~~~~~~~~~~~~~~~~~~~~~~~~~~~~~~~~~~~~~~~~~~~~~~~~~~~
gi|159496970|gb|EY   ~~~~~~~~~~~~~~~~~~~~~~~~~~~~~~~~~~~~~~~~~~~~~~~~~~~~~~~~~~~~~~~~~~~~~~~~~~~~~~~~~~~~~~~~~~
gi|159493588|gb|EY   ~~~~~~~~~~~~~~~~~~~~~~~~~~~~~~~~~~~~~~~~~~~~~~~~~~~~~~~~~~~~~~~~~~~~~~~~~~~~~~~~~~~~~~~~~~
gi|159493287|gb|EY   ~~~~~~~~~~~~~~~~~~~~~~~~~~~~~~~~~~~~~~~~~~~~~~~~~~~~~~~~~~~~~~~~~~~~~~~~~~~~~~~~~~~~~~~~~~
gi|159495054|gb|EY   ~~~~~~~~~~~~~~~~~~~~~~~~~~~~~~~~~~~~~~~~~~~~~~~~~~~~~~~~~~~~~~~~~~~~~~~~~~~~~~~~~~~~~~~~~~
gi|159493125|gb|EY   ~~~~~~~~~~~~~~~~~~~~~~~~~~~~~~~~~~~~~~~~~~~~~~~~~~~~~~~~~~~~~~~~~~~~~~~~~~~~~~~~~~~~~~~~~~
gi|159493667|gb|EY   ~~~~~~~~~~~~~~~~~~~~~~~~~~~~~~~~~~~~~~~~~~~~~~~~~~~~~~~~~~~~~~~~~~~~~~~~~~~~~~~~~~~~~~~~~~
gi|159498247|gb|EY   ~~~~~~~~~~~~~~~~~~~~~~~~~~~~~~~~~~~~~~~~~~~~~~~~~~~~~~~~~~~~~~~~~~~~~~~~~~~~~~~~~~~~~~~~~~

                        370       380       390       400       410       420       430       440       450
                     ....|....|....|....|....|....|....|....|....|....|....|....|....|....|....|....|....|....|
rsmitogenpart        agtaaatctaaatatgctatttttgggtctttacgttcttctagacaagcactttcttttgatattatttttatttttagaattattata
rsnad1               agtaaatctaaatatgctatttttgggtctttacgttcttctagacaagcactttcttttgatattatttttatttttagaattattata
rsatp6               ~~~~~~~~~~~~~~~~~~~~~~~~~~~~~~~~~~~~~~~~~~~~~~~~~~~~~~~~~~~~~~~~~~~~~~~~~~~~~~~~~~~~~~~~~~
rsnad5               ~~~~~~~~~~~~~~~~~~~~~~~~~~~~~~~~~~~~~~~~~~~~~~~~~~~~~~~~~~~~~~~~~~~~~~~~~~~~~~~~~~~~~~~~~~
gi|159496584|gb|EY   ~~~~~~~~~~~~~~~~~~~~~~~~~~~~~~~~~~~~~~~~~~~~~~~~~~~~~~~~~~~~~~~~~~~~~~~~~~~~~~~~~~~~~~~~~~
gi|159496970|gb|EY   ~~~~~~~~~~~~~~~~~~~~~~~~~~~~~~~~~~~~~~~~~~~~~~~~~~~~~~~~~~~~~~~~~~~~~~~~~~~~~~~~~~~~~~~~~~
```

```
gi|159493588|gb|EY
gi|159493287|gb|EY
gi|159495054|gb|EY
gi|159493125|gb|EY
gi|159493667|gb|EY
gi|159498247|gb|EY

                        460       470       480       490       500       510       520       530       540
               ....|....|....|....|....|....|....|....|....|....|....|....|....|....|....|....|
rsmitogenpart  tttattttttataaaaaaaaaatttatttaatattattaatattttttttttggttaaatgttttttttttttttaattttcttttgatt
rsnad1         tttattttttataaaaaaaaaatttatttaatattattaatattttttttttggttaaatgttttttttttttttaattttcttttgatt
rsatp6
rsnad5
gi|159496584|gb|EY
gi|159496970|gb|EY
gi|159493588|gb|EY
gi|159493287|gb|EY
gi|159495054|gb|EY
gi|159493125|gb|EY
gi|159493667|gb|EY
gi|159498247|gb|EY

                        550       560       570       580       590       600       610       620       630
               ....|....|....|....|....|....|....|....|....|....|....|....|....|....|....|....|
rsmitogenpart  atggctgaattaaatcgagctcctttgattttctgagggtgagagtgagttagttagtggttaaaatttagagttaggaagtttattt
rsnad1         atggctgaattaaatcgagctcctttgattttctgagggtgagagtgagttagttagtggttaaaatttagagttaggaagtttattt
rsatp6
rsnad5
gi|159496584|gb|EY
gi|159496970|gb|EY
gi|159493588|gb|EY
gi|159493287|gb|EY
gi|159495054|gb|EY
gi|159493125|gb|EY
gi|159493667|gb|EY
gi|159498247|gb|EY

                        640       650       660       670       680       690       700       710       720
               ....|....|....|....|....|....|....|....|....|....|....|....|....|....|....|....|
rsmitogenpart  tttattttttttttttaagtgaataaggtttttagtttaataagttttattttaaattaattattttttaattaaaattaattattt
rsnad1         tttattttttttttttaagtgaataaggtttttagtttaataagttttattttaaattaattattttttaattaaaattaattattt
rsatp6
rsnad5
gi|159496584|gb|EY ~~~~~TTTTTTTTTAAGTGGATAAAGGTTTTTTAGTTTAATAAGTTTTATTTT~AAATTAATTATTTTTTAAATAAAATTAATTATTT
gi|159496970|gb|EY
gi|159493588|gb|EY
gi|159493287|gb|EY
gi|159495054|gb|EY
gi|159493125|gb|EY
gi|159493667|gb|EY
gi|159498247|gb|EY

                        730       740       750       760       770       780       790       800       810
               ....|....|....|....|....|....|....|....|....|....|....|....|....|....|....|....|
rsmitogenpart  ggttttattattttttttattattatttttattcgtagtgttttacctcgttatcgttatgatatattaataagttttttttgattatta
rsnad1         ggttttattattttttttattattatttttattcgtagtgttttacctcgttatcgttatgatatattaataagttttttttgattatta
rsatp6
rsnad5
gi|159496584|gb|EY GGTTTTATTATTTTTTTT~ATTATTATTTTTATTCGTAGTGTTTTACCTCGTTATCGTTATGATATATTAATAAGTTTTTTTTGATTATTA
gi|159496970|gb|EY
gi|159493588|gb|EY
gi|159493287|gb|EY
gi|159495054|gb|EY
gi|159493125|gb|EY
gi|159493667|gb|EY
gi|159498247|gb|EY

                        820       830       840       850       860       870       880       890       900
               ....|....|....|....|....|....|....|....|....|....|....|....|....|....|....|....|
rsmitogenpart  ctttttattattttatttattttttttttttattattgattattttgttttagtattataaatataattttttgttttaatgatattttatt
rsnad1         ctttttattattttatttattttttttttttattattgattattttgttttag~~~~
rsatp6         ~~~~~~~~~~~~~~~~~~~~~~~~~~~~~~~~~~~~~~~~~~~~~~~~~~~~~~~~ataaatataattttgttttaatgatattttatt
rsnad5
gi|159496584|gb|EY CTTTTTATTATTTTATTTATTTTTTTTTTTATTATTGATTATTTTGTTTTAGTATTATAAATATAATTTTTGTTTTAATGATATTTTTATT
gi|159496970|gb|EY
gi|159493588|gb|EY
gi|159493287|gb|EY
gi|159495054|gb|EY
gi|159493125|gb|EY
gi|159493667|gb|EY
gi|159498247|gb|EY

                        910       920       930       940       950       960       970       980       990
               ....|....|....|....|....|....|....|....|....|....|....|....|....|....|....|....|
rsmitogenpart  gaattttttttttattagatatattttttttaaaaattaaaatttttttaaaggttataatgatttattattaaaataatttaattttttt
rsnad1
rsatp6         gaattttttttttattagatatattttttttaaaaattaaaatttttttaaaggttataatgatttattattaaaataatttaattttttt
rsnad5
gi|159496584|gb|EY GAATTTTTTTTT~ATTAGATATATTTTTTTTAAAAATTAAAATTTTTTAAAGGTTATAATGATTTATTATTAAAATAATTTAATTTTTTT
gi|159496970|gb|EY
gi|159493588|gb|EY

gi|159493287|gb|EY
gi|159495054|gb|EY
gi|159493125|gb|EY
gi|159493667|gb|EY
gi|159498247|gb|EY

                        1000      1010      1020      1030      1040      1050      1060      1070      1080
               ....|....|....|....|....|....|....|....|....|....|....|....|....|....|....|....|
rsmitogenpart  ttatttaaattttaatttagatttaattatatcgtatttttatttgattcatttttataagatctgattttgtttgttttttttagtta
rsnad1
rsatp6         ttatttaaattttaatttagatttaattatatcgtatttttatttgattcatttttataagatctgattttgtttgttttttttagtta
rsnad5
gi|159496584|gb|EY TTATTTAAATTTTAATTTAGATTTAATTATATCGTATTTTTATTTGATTCATTTTTATAAGATCTGATTTTGTTTGTTTTTTTTAGTTA
gi|159496970|gb|EY ~~~~~~~~~~~~~~~~~~~~~~~~~~~~~~~~~~~~~~~~TTTTTTTTTTTTTTTATAAGATCTGATTTTGTTTGTTTTTTTTAGTTA
gi|159493588|gb|EY
gi|159493287|gb|EY
gi|159495054|gb|EY
gi|159493125|gb|EY
gi|159493667|gb|EY
gi|159498247|gb|EY

                        1090      1100      1110      1120      1130      1140      1150      1160      1170
               ....|....|....|....|....|....|....|....|....|....|....|....|....|....|....|....|
rsmitogenpart  attaccttttttttaaagttttttttataaataattccattaattactttataattatttttttatagtttttattactatattaataaa
rsnad1
rsatp6         attaccttttttttaaagttttttttataaataattccattaattactttataattatttttttatagtttttattactatattaataaa
rsnad5
gi|159496584|gb|EY ATTACCTTTTTTTTAAAGTTTTTTTT~ATAAATAATTCCATTAATTACTTTATAATTATTTTTTATAGTTTTTATTACTATATTAATAAA
gi|159496970|gb|EY ATTACCTTTTTTTTAAAGTTTTTTTT~ATAAATAATTCCATTAATTACTTTATAATTATTTTTTATAGTTTTTATTACTATATTAATAAA
gi|159493588|gb|EY
gi|159493287|gb|EY
gi|159495054|gb|EY
gi|159493125|gb|EY
gi|159493667|gb|EY
gi|159498247|gb|EY

                        1180      1190      1200      1210      1220      1230      1240      1250      1260
               ....|....|....|....|....|....|....|....|....|....|....|....|....|....|....|....|
rsmitogenpart  cattagttttactataagtttaattagaggtgatattgaattaaagataatataagagattttttgttatggaattatttggtaatttaat
rsnad1
rsatp6         cattagttttactataagtttaattagaggtgatattgaattaaagataatataagagattttttgttatggaattatttggtaatttaat
rsnad5
gi|159496584|gb|EY CATTAGTTTTACTATAAGTTTAATTAGAGGTGATATTGAATTTTATCTCCCGGCC~~~~
gi|159496970|gb|EY CATTAGTTTTACTATAAGTTTAATTAGAGGTGATATTGAATTAAAGATAATATAAGAGATTTTTTGTTATGGAATTATTTGGTAATTTAAT
gi|159493588|gb|EY
gi|159493287|gb|EY
gi|159495054|gb|EY
gi|159493125|gb|EY
gi|159493667|gb|EY
gi|159498247|gb|EY

                        1270      1280      1290      1300      1310      1320      1330      1340      1350
               ....|....|....|....|....|....|....|....|....|....|....|....|....|....|....|....|
rsmitogenpart  tcgtccaattgctttatttatacgtttattaattaatttaattttaggacattttattaagttaattattgtaagttttattttagctaa
rsnad1
rsatp6         tcgtccaattgctttatttatacgtttattaattaatttaattttaggacattttattaagttaattattgtaagttttattttagctaa
rsnad5
gi|159496584|gb|EY
gi|159496970|gb|EY TCGTCCAATTGCTTTATTTATACGTTTATTAATTAATTTAATTTTAGGACATTTTATTAAGTTAATTATTGTAAGTTTTATTTTAGCTAA
gi|159493588|gb|EY ~~~~~~~~~~~~~~~~TTTATACGTTTATTAATTAATTTAATTTTAGGACATTTTATTAAGTTAATTATTGTAAGTTTTATTTTAGCTAA
gi|159493287|gb|EY
gi|159495054|gb|EY
gi|159493125|gb|EY
gi|159493667|gb|EY
gi|159498247|gb|EY

                        1360      1370      1380      1390      1400      1410      1420      1430      1440
               ....|....|....|....|....|....|....|....|....|....|....|....|....|....|....|....|
rsmitogenpart  ttaatgaaattatattttttttgattatttttctatattagttgaattttttagtaattttagttcagttacatattttttttagttttaat
rsnad1
rsatp6         ttaatgaaattatattttttttgattatttttctatattagttgaattttttagtaattttagttcagttacatattttttttagttttaat
rsnad5
gi|159496584|gb|EY
gi|159496970|gb|EY TTAATGAAATTATATTTTTTTTGATTATTTTTCTATATTAGTTGAATTTTTTAGTAGTTTTAGTTCAGTTACATATTTTTTTTAGTTTTAAT
gi|159493588|gb|EY TTAATGAAATTATATTTTTTTTGATTATTTTTCTATATTAGTTGAATTTTTTAGTAATTTTAGTTCAGTTACATATTTTTTTTAGTTTTAAT
gi|159493287|gb|EY
gi|159495054|gb|EY
gi|159493125|gb|EY
gi|159493667|gb|EY
gi|159498247|gb|EY

                        1450      1460      1470      1480      1490      1500      1510      1520      1530
               ....|....|....|....|....|....|....|....|....|....|....|....|....|....|....|....|
rsmitogenpart  tatattttaattaggtgagtagtgaagaattttttttttatattttttattttaatttattttttttatttattaaggtttaattttttttt
rsnad1
rsatp6         tatattttaattaggtgagtag~~~~
rsnad5         ~~~~~~~~~~~~~~~~~~~~~~~~~~~~~~tttttttatatttttttattttaatttattttttttatttattaaggtttaattttttttt
gi|159496584|gb|EY
gi|159496970|gb|EY TATATTTTAATTAGGTGAGTAGTGAAGAATTTTTTTTTATATTTTTTATTTTAATTTATTTTTTTTATTTATTAAGGTTTAATTTTTTTT
gi|159493588|gb|EY TATATTTTAATTAGGTGAGTAGTGAAGAATTTTTTTT~ATATTTTTTATTTTAATTTATTTTTTTTATTTATTAAGGTTTAATTTTTTTT
gi|159493287|gb|EY ~~~~~~~~~~~~~~~~~~GGCCGGGGAATTTTTTTTTATG TTTTTTATTTTAATTTATTTTTTTTATTTATTAAGGTTTAATTTTTTTT
```

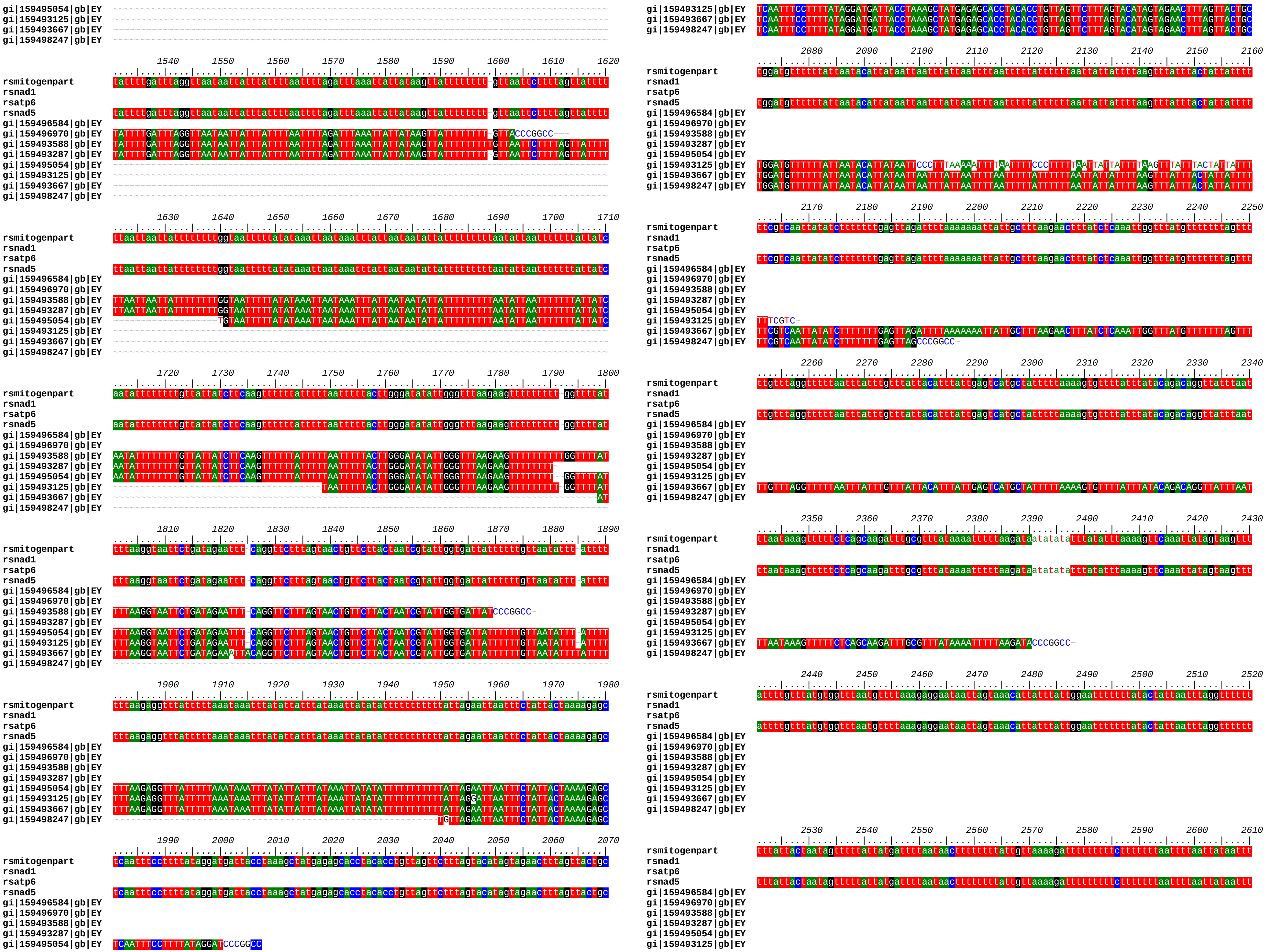

```
gi|159493667|gb|EY
gi|159498247|gb|EY

                          2620      2630      2640      2650      2660      2670      2680      2690      2700
                    ....|....|....|....|....|....|....|....|....|....|....|....|....|....|....|....|....|....|
rsmitogenpart       taattatttgaattttagttttatttatttttttttaactttaatattaatttttttttctttgtttttaatttaatatttaagttaaaa
rsnad1
rsatp6
rsnad5              taattatttgaattttagttttatttatttttttttaactttaatattaatttttttttctttgtttttaatttaatatttaagttaaaa
gi|159496584|gb|EY
gi|159496970|gb|EY
gi|159493588|gb|EY
gi|159493287|gb|EY
gi|159495054|gb|EY
gi|159493125|gb|EY
gi|159493667|gb|EY
gi|159498247|gb|EY

                          2710      2720      2730      2740      2750      2760      2770      2780      2790
                    ....|....|....|....|....|....|....|....|....|....|....|....|....|....|....|....|....|....|
rsmitogenpart       ttttttttttttaagagtaatatttctttatttagatttttatttaattattttttaaattgttataataataatagttttttttttgtt
rsnad1
rsatp6
rsnad5              ttttttttttttaagagtaatatttctttatttagatttttatttaattattttttaaattgttataataataatagttttttttttgtt
gi|159496584|gb|EY
gi|159496970|gb|EY
gi|159493588|gb|EY
gi|159493287|gb|EY
gi|159495054|gb|EY
gi|159493125|gb|EY
gi|159493667|gb|EY
gi|159498247|gb|EY

                          2800      2810      2820      2830      2840      2850      2860      2870      2880
                    ....|....|....|....|....|....|....|....|....|....|....|....|....|....|....|....|....|....|
rsmitogenpart       ttttaattatctttattttttttttgaaataaaagtaattaagagattaatatttatttttattattaggtttattttattaaaactttttt
rsnad1
rsatp6
rsnad5              ttttaattatctttattttttttttgaaataaaagtaattaagagattaatatttatttttattattaggtttattttattaaaactttttt
gi|159496584|gb|EY
gi|159496970|gb|EY
gi|159493588|gb|EY
gi|159493287|gb|EY
gi|159495054|gb|EY
gi|159493125|gb|EY
gi|159493667|gb|EY
gi|159498247|gb|EY

                          2890      2900      2910      2920      2930      2940      2950      2960      2970
                    ....|....|....|....|....|....|....|....|....|....|....|....|....|....|....|....|....|....|
rsmitogenpart       aattaataatattttttttaatatatttttgttttttttgaattttttttaattttatttttaataaaattaataatttaggttaatattt
rsnad1
rsatp6
rsnad5              aattaataatattttttttaatatatttttgttttttttgaattttttttaattttatttttaataaaattaataatttaggttaatattt
gi|159496584|gb|EY
gi|159496970|gb|EY
gi|159493588|gb|EY
gi|159493287|gb|EY
gi|159495054|gb|EY
gi|159493125|gb|EY
gi|159493667|gb|EY
gi|159498247|gb|EY

                          2980      2990      3000      3010
                    ....|....|....|....|....|....|....|....|....|.
rsmitogenpart       tttattagtttttttgattattataattatattgatttttgtttag
rsnad1
rsatp6
rsnad5              tttattagtttttttgattattataattatattgatttttgtttag
gi|159496584|gb|EY
gi|159496970|gb|EY
gi|159493588|gb|EY
gi|159493287|gb|EY
gi|159495054|gb|EY
gi|159493125|gb|EY
gi|159493667|gb|EY
gi|159498247|gb|EY
```

EST matching from *cox2* to end of coding region

```
                            10        20        30        40        50        60        70        80        90
                    ....|....|....|....|....|....|....|....|....|....|....|....|....|....|....|....|....|....|
rsmitogen           ttttttaattaaaatatatttagtatttttaataggttagtaattaagttagattgatttcataattttaattgtattttattattatgt
rscox2              ttttttaattaaaatatatttagtatttttaataggttagtaattaagttagattgatttcataattttaattgtattttattattatgt
rstrna-his          ~~~~~~~~~~~~~~~~~~~~~~~~~~~~~~~~~~~~~~~~~~~~~~~~~~~~~~~~~~~~~~~~~~~~~~~~~~~~~~~~~~~~~~~~~~
rs16s               ~~~~~~~~~~~~~~~~~~~~~~~~~~~~~~~~~~~~~~~~~~~~~~~~~~~~~~~~~~~~~~~~~~~~~~~~~~~~~~~~~~~~~~~~~~
rsnad3              ~~~~~~~~~~~~~~~~~~~~~~~~~~~~~~~~~~~~~~~~~~~~~~~~~~~~~~~~~~~~~~~~~~~~~~~~~~~~~~~~~~~~~~~~~~
rstrna-leu          ~~~~~~~~~~~~~~~~~~~~~~~~~~~~~~~~~~~~~~~~~~~~~~~~~~~~~~~~~~~~~~~~~~~~~~~~~~~~~~~~~~~~~~~~~~
rscox3              ~~~~~~~~~~~~~~~~~~~~~~~~~~~~~~~~~~~~~~~~~~~~~~~~~~~~~~~~~~~~~~~~~~~~~~~~~~~~~~~~~~~~~~~~~~
rstrna-pro          ~~~~~~~~~~~~~~~~~~~~~~~~~~~~~~~~~~~~~~~~~~~~~~~~~~~~~~~~~~~~~~~~~~~~~~~~~~~~~~~~~~~~~~~~~~
rstrna-arg          ~~~~~~~~~~~~~~~~~~~~~~~~~~~~~~~~~~~~~~~~~~~~~~~~~~~~~~~~~~~~~~~~~~~~~~~~~~~~~~~~~~~~~~~~~~
rsnad6              ~~~~~~~~~~~~~~~~~~~~~~~~~~~~~~~~~~~~~~~~~~~~~~~~~~~~~~~~~~~~~~~~~~~~~~~~~~~~~~~~~~~~~~~~~~
rsnad4L             ~~~~~~~~~~~~~~~~~~~~~~~~~~~~~~~~~~~~~~~~~~~~~~~~~~~~~~~~~~~~~~~~~~~~~~~~~~~~~~~~~~~~~~~~~~
rstrna-val          ~~~~~~~~~~~~~~~~~~~~~~~~~~~~~~~~~~~~~~~~~~~~~~~~~~~~~~~~~~~~~~~~~~~~~~~~~~~~~~~~~~~~~~~~~~
rstrna-leu          ~~~~~~~~~~~~~~~~~~~~~~~~~~~~~~~~~~~~~~~~~~~~~~~~~~~~~~~~~~~~~~~~~~~~~~~~~~~~~~~~~~~~~~~~~~
159494961oke        ~~~~~~~~~~~~~~~~~~~~~~~~~~~~~~~~~~~~~~~~~~~~~~~~~~~~~~~~~~~~~~~~~~~~~~~~~~~~~~~~~~~~~~~~~~
159496557oke        ~~~~~~~~~~~~~~~~~~~~~~~~~~~~~~~~~~~~~~~~~~~~~~~~~~~~~~~~~~~~~~~~~~~~~~~~~~~~~~~~~~~~~~~~~~
159497093oke        ~~~~~~~~~~~~~~~~~~~~~~~~~~~~~~~~~~~~~~~~~~~~~~~~~~~~~~~~~~~~~~~~~~~~~~~~~~~~~~~~~~~~~~~~~~
159496622oke        ~~~~~~~~~~~~~~~~~~~~~~~~~~~~~~~~~~~~~~~~~~~~~~~~~~~~~~~~~~~~~~~~~~~~~~~~~~~~~~~~~~~~~~~~~~
159498117oke        ~~~~~~~~~~~~~~~~~~~~~~~~~~~~~~~~~~~~~~~~~~~~~~~~~~~~~~~~~~~~~~~~~~~~~~~~~~~~~~~~~~~~~~~~~~
159494290oke        ~~~~~~~~~~~~~~~~~~~~~~~~~~~~~~~~~~~~~~~~~~~~~~~~~~~~~~~~~~~~~~~~~~~~~~~~~~~~~~~~~~~~~~~~~~
159495636oke        ~~~~~~~~~~~~~~~~~~~~~~~~~~~~~~~~~~~~~~~~~~~~~~~~~~~~~~~~~~~~~~~~~~~~~~~~~~~~~~~~~~~~~~~~~~
159497178oke        ~~~~~~~~~~~~~~~~~~~~~~~~~~~~~~~~~~~~~~~~~~~~~~~~~~~~~~~~~~~~~~~~~~~~~~~~~~~~~~~~~~~~~~~~~~
159498304oke        ~~~~~~~~~~~~~~~~~~~~~~~~~~~~~~~~~~~~~~~~~~~~~~~~~~~~~~~~~~~~~~~~~~~~~~~~~~~~~~~~~~~~~~~~~~
159496326oke        ~~~~~~~~~~~~~~~~~~~~~~~~~~~~~~~~~~~~~~~~~~~~~~~~~~~~~~~~~~~~~~~~~~~~~~~~~~~~~~~~~~~~~~~~~~
159495404oke        ~~~~~~~~~~~~~~~~~~~~~~~~~~~~~~~~~~~~~~~~~~~~~~~~~~~~~~~~~~~~~~~~~~~~~~~~~~~~~~~~~~~~~~~~~~
159493141oke        ~~~~~~~~~~~~~~~~~~~~~~~~~~~~~~~~~~~~~~~~~~~~~~~~~~~~~~~~~~~~~~~~~~~~~~~~~~~~~~~~~~~~~~~~~~
159493570oke        ~~~~~~~~~~~~~~~~~~~~~~~~~~~~~~~~~~~~~~~~~~~~~~~~~~~~~~~~~~~~~~~~~~~~~~~~~~~~~~~~~~~~~~~~~~
159493499oke        ~~~~~~~~~~~~~~~~~~~~~~~~~~~~~~~~~~~~~~~~~~~~~~~~~~~~~~~~~~~~~~~~~~~~~~~~~~~~~~~~~~~~~~~~~~
159493217oke        ~~~~~~~~~~~~~~~~~~~~~~~~~~~~~~~~~~~~~~~~~~~~~~~~~~~~~~~~~~~~~~~~~~~~~~~~~~~~~~~~~~~~~~~~~~
159493216oke        ~~~~~~~~~~~~~~~~~~~~~~~~~~~~~~~~~~~~~~~~~~~~~~~~~~~~~~~~~~~~~~~~~~~~~~~~~~~~~~~~~~~~~~~~~~
159493234oke        ~~~~~~~~~~~~~~~~~~~~~~~~~~~~~~~~~~~~~~~~~~~~~~~~~~~~~~~~~~~~~~~~~~~~~~~~~~~~~~~~~~~~~~~~~~
159492908oke        ~~~~~~~~~~~~~~~~~~~~~~~~~~~~~~~~~~~~~~~~~~~~~~~~~~~~~~~~~~~~~~~~~~~~~~~~~~~~~~~~~~~~~~~~~~
159491934oke        ~~~~~~~~~~~~~~~~~~~~~~~~~~~~~~~~~~~~~~~~~~~~~~~~~~~~~~~~~~~~~~~~~~~~~~~~~~~~~~~~~~~~~~~~~~
159492538           ~~~~~~~~~~~~~~~~~~~~~~~~~~~~~~~~~~~~~~~~~~~~~~~~~~~~~~~~~~~~~~~~~~~~~~~~~~~~~~~~~~~~~~~~~~
159498161           ~~~~~~~~~~~~~~~~~~~~~~~~~~~~~~~~~~~~~~~~~~~~~~~~~~~~~~~~~~~~~~~~~~~~~~~~~~~~~~~~~~~~~~~~~~
159493124           ~~~~~~~~~~~~~~~~~~~~~~~~~~~~~~~~~~~~~~~~~~~~~~~~~~~~~~~~~~~~~~~~~~~~~~~~~~~~~~~~~~~~~~~~~~
159493661           ~~~~~~~~~~~~~~~~~~~~~~~~~~~~~~~~~~~~~~~~~~~~~~~~~~~~~~~~~~~~~~~~~~~~~~~~~~~~~~~~~~~~~~~~~~
159494727           ~~~~~~~~~~~~~~~~~~~~~~~~~~~~~~~~~~~~~~~~~~~~~~~~~~~~~~~~~~~~~~~~~~~~~~~~~~~~~~~~~~~~~~~~~~
159496944           ~~~~~~~~~~~~~~~~~~~~~~~~~~~~~~~~~~~~~~~~~~~~~~~~~~~~~~~~~~~~~~~~~~~~~~~~~~~~~~~~~~~~~~~~~~
159494492           ~~~~~~~~~~~~~~~~~~~~~~~~~~~~~~~~~~~~~~~~~~~~~~~~~~~~~~~~~~~~~~~~~~~~~~~~~~~~~~~~~~~~~~~~~~
159496140           ~~~~~~~~~~~~~~~~~~~~~~~~~~~~~~~~~~~~~~~~~~~~~~~~~~~~~~~~~~~~~~~~~~~~~~~~~~~~~~~~~~~~~~~~~~
159497160           ~~~~~~~~~~~~~~~~~~~~~~~~~~~~~~~~~~~~~~~~~~~~~~~~~~~~~~~~~~~~~~~~~~~~~~~~~~~~~~~~~~~~~~~~~~
aaa07c02.g1         ~~~~~~~~~~~~~~~~~~~~~~~~~~~~~~~~~~~~~~~~~~~~~~~~~~~~~~~~~~~~~~~~~~~~~~~~~~~~~~~~~~~~~~~~~~

                           100       110       120       130       140       150       160       170       180
                    ....|....|....|....|....|....|....|....|....|....|....|....|....|....|....|....|....|....|
rsmitogen           attgtttattttgttttatttttatttttgatatttttttttgttaaattttaattttaaattaaatttgagtttttataatttagaatta
rscox2              attgtttattttgttttatttttatttttgatatttttttttgttaaattttaattttaaattaaatttgagtttttataatttagaatta
rstrna-his          ~~~~~~~~~~~~~~~~~~~~~~~~~~~~~~~~~~~~~~~~~~~~~~~~~~~~~~~~~~~~~~~~~~~~~~~~~~~~~~~~~~~~~~~~~~
rs16s               ~~~~~~~~~~~~~~~~~~~~~~~~~~~~~~~~~~~~~~~~~~~~~~~~~~~~~~~~~~~~~~~~~~~~~~~~~~~~~~~~~~~~~~~~~~
rsnad3              ~~~~~~~~~~~~~~~~~~~~~~~~~~~~~~~~~~~~~~~~~~~~~~~~~~~~~~~~~~~~~~~~~~~~~~~~~~~~~~~~~~~~~~~~~~
rstrna-leu          ~~~~~~~~~~~~~~~~~~~~~~~~~~~~~~~~~~~~~~~~~~~~~~~~~~~~~~~~~~~~~~~~~~~~~~~~~~~~~~~~~~~~~~~~~~
rscox3              ~~~~~~~~~~~~~~~~~~~~~~~~~~~~~~~~~~~~~~~~~~~~~~~~~~~~~~~~~~~~~~~~~~~~~~~~~~~~~~~~~~~~~~~~~~
rstrna-pro          ~~~~~~~~~~~~~~~~~~~~~~~~~~~~~~~~~~~~~~~~~~~~~~~~~~~~~~~~~~~~~~~~~~~~~~~~~~~~~~~~~~~~~~~~~~
rstrna-arg          ~~~~~~~~~~~~~~~~~~~~~~~~~~~~~~~~~~~~~~~~~~~~~~~~~~~~~~~~~~~~~~~~~~~~~~~~~~~~~~~~~~~~~~~~~~
rsnad6              ~~~~~~~~~~~~~~~~~~~~~~~~~~~~~~~~~~~~~~~~~~~~~~~~~~~~~~~~~~~~~~~~~~~~~~~~~~~~~~~~~~~~~~~~~~
rsnad4L             ~~~~~~~~~~~~~~~~~~~~~~~~~~~~~~~~~~~~~~~~~~~~~~~~~~~~~~~~~~~~~~~~~~~~~~~~~~~~~~~~~~~~~~~~~~
rstrna-val          ~~~~~~~~~~~~~~~~~~~~~~~~~~~~~~~~~~~~~~~~~~~~~~~~~~~~~~~~~~~~~~~~~~~~~~~~~~~~~~~~~~~~~~~~~~
rstrna-leu          ~~~~~~~~~~~~~~~~~~~~~~~~~~~~~~~~~~~~~~~~~~~~~~~~~~~~~~~~~~~~~~~~~~~~~~~~~~~~~~~~~~~~~~~~~~
159494961oke        ~~~~~~~~~~~~~~~~~~~~~~~~~~~~~~~~~~~~~~~~~~~~~~~~~~~~~~~~~~~~~~~~~~~~~~~~~~~~~~~~~~~~~~~~~A
159496557oke        ~~~~~~~~~~~~~~~~~~~~~~~~~~~~~~~~~~~~~~~~~~~~~~~~~~~~~~~~~~~~~~~~~~~~~~~~~~~~~~~~~~~~~~~~~A
159497093oke        ~~~~~~~~~~~~~~~~~~~~~~~~~~~~~~~~~~~~~~~~~~~~~~~~~~~~~~~~~~~~~~~~~~~~~~~~~~~~~~~~~~~~~~~~~~
159496622oke        ~~~~~~~~~~~~~~~~~~~~~~~~~~~~~~~~~~~~~~~~~~~~~~~~~~~~~~~~~~~~~~~~~~~~~~~~~~~~~~~~~~~~~~~~~~
159498117oke        ~~~~~~~~~~~~~~~~~~~~~~~~~~~~~~~~~~~~~~~~~~~~~~~~~~~~~~~~~~~~~~~~~~~~~~~~~~~~~~~~~~~~~~~~~~
159494290oke        ~~~~~~~~~~~~~~~~~~~~~~~~~~~~~~~~~~~~~~~~~~~~~~~~~~~~~~~~~~~~~~~~~~~~~~~~~~~~~~~~~~~~~~~~~~
159495636oke        ~~~~~~~~~~~~~~~~~~~~~~~~~~~~~~~~~~~~~~~~~~~~~~~~~~~~~~~~~~~~~~~~~~~~~~~~~~~~~~~~~~~~~~~~~~
159497178oke        ~~~~~~~~~~~~~~~~~~~~~~~~~~~~~~~~~~~~~~~~~~~~~~~~~~~~~~~~~~~~~~~~~~~~~~~~~~~~~~~~~~~~~~~~~~
159498304oke        ~~~~~~~~~~~~~~~~~~~~~~~~~~~~~~~~~~~~~~~~~~~~~~~~~~~~~~~~~~~~~~~~~~~~~~~~~~~~~~~~~~~~~~~~~~
159496326oke        ~~~~~~~~~~~~~~~~~~~~~~~~~~~~~~~~~~~~~~~~~~~~~~~~~~~~~~~~~~~~~~~~~~~~~~~~~~~~~~~~~~~~~~~~~~
159495404oke        ~~~~~~~~~~~~~~~~~~~~~~~~~~~~~~~~~~~~~~~~~~~~~~~~~~~~~~~~~~~~~~~~~~~~~~~~~~~~~~~~~~~~~~~~~~
159493141oke        ~~~~~~~~~~~~~~~~~~~~~~~~~~~~~~~~~~~~~~~~~~~~~~~~~~~~~~~~~~~~~~~~~~~~~~~~~~~~~~~~~~~~~~~~~~
159493570oke        ~~~~~~~~~~~~~~~~~~~~~~~~~~~~~~~~~~~~~~~~~~~~~~~~~~~~~~~~~~~~~~~~~~~~~~~~~~~~~~~~~~~~~~~~~~
159493499oke        ~~~~~~~~~~~~~~~~~~~~~~~~~~~~~~~~~~~~~~~~~~~~~~~~~~~~~~~~~~~~~~~~~~~~~~~~~~~~~~~~~~~~~~~~~~
159493217oke        ~~~~~~~~~~~~~~~~~~~~~~~~~~~~~~~~~~~~~~~~~~~~~~~~~~~~~~~~~~~~~~~~~~~~~~~~~~~~~~~~~~~~~~~~~~
159493216oke        ~~~~~~~~~~~~~~~~~~~~~~~~~~~~~~~~~~~~~~~~~~~~~~~~~~~~~~~~~~~~~~~~~~~~~~~~~~~~~~~~~~~~~~~~~~
159493234oke        ~~~~~~~~~~~~~~~~~~~~~~~~~~~~~~~~~~~~~~~~~~~~~~~~~~~~~~~~~~~~~~~~~~~~~~~~~~~~~~~~~~~~~~~~~~
159492908oke        ~~~~~~~~~~~~~~~~~~~~~~~~~~~~~~~~~~~~~~~~~~~~~~~~~~~~~~~~~~~~~~~~~~~~~~~~~~~~~~~~~~~~~~~~~~
159491934oke        ~~~~~~~~~~~~~~~~~~~~~~~~~~~~~~~~~~~~~~~~~~~~~~~~~~~~~~~~~~~~~~~~~~~~~~~~~~~~~~~~~~~~~~~~~~
159492538           ~~~~~~~~~~~~~~~~~~~~~~~~~~~~~~~~~~~~~~~~~~~~~~~~~~~~~~~~~~~~~~~~~~~~~~~~~~~~~~~~~~~~~~~~~~
159498161           ~~~~~~~~~~~~~~~~~~~~~~~~~~~~~~~~~~~~~~~~~~~~~~~~~~~~~~~~~~~~~~~~~~~~~~~~~~~~~~~~~~~~~~~~~~
159493124           ~~~~~~~~~~~~~~~~~~~~~~~~~~~~~~~~~~~~~~~~~~~~~~~~~~~~~~~~~~~~~~~~~~~~~~~~~~~~~~~~~~~~~~~~~~
159493661           ~~~~~~~~~~~~~~~~~~~~~~~~~~~~~~~~~~~~~~~~~~~~~~~~~~~~~~~~~~~~~~~~~~~~~~~~~~~~~~~~~~~~~~~~~~
159494727           ~~~~~~~~~~~~~~~~~~~~~~~~~~~~~~~~~~~~~~~~~~~~~~~~~~~~~~~~~~~~~~~~~~~~~~~~~~~~~~~~~~~~~~~~~~
159496944           ~~~~~~~~~~~~~~~~~~~~~~~~~~~~~~~~~~~~~~~~~~~~~~~~~~~~~~~~~~~~~~~~~~~~~~~~~~~~~~~~~~~~~~~~~~
159494492           ~~~~~~~~~~~~~~~~~~~~~~~~~~~~~~~~~~~~~~~~~~~~~~~~~~~~~~~~~~~~~~~~~~~~~~~~~~~~~~~~~~~~~~~~~~
159496140           ~~~~~~~~~~~~~~~~~~~~~~~~~~~~~~~~~~~~~~~~~~~~~~~~~~~~~~~~~~~~~~~~~~~~~~~~~~~~~~~~~~~~~~~~~~
159497160           ~~~~~~~~~~~~~~~~~~~~~~~~~~~~~~~~~~~~~~~~~~~~~~~~~~~~~~~~~~~~~~~~~~~~~~~~~~~~~~~~~~~~~~~~~~
aaa07c02.g1         ~~~~~~~~~~~~~~~~~~~~~~~~~~~~~~~~~~~~~~~~~~~~~~~~~~~~~~~~~~~~~~~~~~~~~~~~~~~~~~~~~~~~~~~~~~

                           190       200       210       220       230       240       250       260       270
                    ....|....|....|....|....|....|....|....|....|....|....|....|....|....|....|....|....|....|
rsmitogen           ttaggttgttttttacctatattaattttattaatacagatattaccttctttaatttttttataaaatttacattatataggttaagaa
rscox2              ttaggttgttttttacctatattaattttattaatacagatattaccttctttaatttttttataaaatttacattatataggttaagaa
rstrna-his          ~~~~~~~~~~~~~~~~~~~~~~~~~~~~~~~~~~~~~~~~~~~~~~~~~~~~~~~~~~~~~~~~~~~~~~~~~~~~~~~~~~~~~~~~~~
rs16s               ~~~~~~~~~~~~~~~~~~~~~~~~~~~~~~~~~~~~~~~~~~~~~~~~~~~~~~~~~~~~~~~~~~~~~~~~~~~~~~~~~~~~~~~~~~
```

```
rsnad3           ~~~~~~~~~~~~~~~~~~~~~~~~~~~~~~~~~~~~~~~~~~~~~~~~~~~~~~~~~~~~~~~~~~~~~~~~~~~~~~~~~~~~~~~~~~
rstrna-leu       ~~~~~~~~~~~~~~~~~~~~~~~~~~~~~~~~~~~~~~~~~~~~~~~~~~~~~~~~~~~~~~~~~~~~~~~~~~~~~~~~~~~~~~~~~~
rscox3           ~~~~~~~~~~~~~~~~~~~~~~~~~~~~~~~~~~~~~~~~~~~~~~~~~~~~~~~~~~~~~~~~~~~~~~~~~~~~~~~~~~~~~~~~~~
rstrna-pro       ~~~~~~~~~~~~~~~~~~~~~~~~~~~~~~~~~~~~~~~~~~~~~~~~~~~~~~~~~~~~~~~~~~~~~~~~~~~~~~~~~~~~~~~~~~
rstrna-arg       ~~~~~~~~~~~~~~~~~~~~~~~~~~~~~~~~~~~~~~~~~~~~~~~~~~~~~~~~~~~~~~~~~~~~~~~~~~~~~~~~~~~~~~~~~~
rsnad6           ~~~~~~~~~~~~~~~~~~~~~~~~~~~~~~~~~~~~~~~~~~~~~~~~~~~~~~~~~~~~~~~~~~~~~~~~~~~~~~~~~~~~~~~~~~
rsnad4L          ~~~~~~~~~~~~~~~~~~~~~~~~~~~~~~~~~~~~~~~~~~~~~~~~~~~~~~~~~~~~~~~~~~~~~~~~~~~~~~~~~~~~~~~~~~
rstrna-val       ~~~~~~~~~~~~~~~~~~~~~~~~~~~~~~~~~~~~~~~~~~~~~~~~~~~~~~~~~~~~~~~~~~~~~~~~~~~~~~~~~~~~~~~~~~
rstrna-leu       ~~~~~~~~~~~~~~~~~~~~~~~~~~~~~~~~~~~~~~~~~~~~~~~~~~~~~~~~~~~~~~~~~~~~~~~~~~~~~~~~~~~~~~~~~~
159494961oke     TTAGGTTGTTTTTTACCTATATTAATTTTATTAATACAGATATTACCTTCTTTAATTTTTTTATAAAATTTACATTATATAGGTTAAGAA
159496557oke     TTAGGTTGTTTTTTACCTATATTAATTTTATTAATACAGATATTACCTTCTTTAATTTTTTTATAAAATTTACATTATATAGGTTAAGAA
159497093oke     ~~~~~~~~~~~~~~~~~~~~~~~~~~~~~~~~~~~~~~~~~~~~~~~~~~~~~~~~~~~~~~~~~~~~~~~~~~~~~~~~~~~~~~~~~~
159496622oke     ~~~~~~~~~~~~~~~~~~~~~~~~~~~~~~~~~~~~~~~~~~~~~~~~~~~~~~~~~~~~~~~~~~~~~~~~~~~~~~~~~~~~~~~~~~
159498117oke     ~~~~~~~~~~~~~~~~~~~~~~~~~~~~~~~~~~~~~~~~~~~~~~~~~~~~~~~~~~~~~~~~~~~~~~~~~~~~~~~~~~~~~~~~~~
159494290oke     ~~~~~~~~~~~~~~~~~~~~~~~~~~~~~~~~~~~~~~~~~~~~~~~~~~~~~~~~~~~~~~~~~~~~~~~~~~~~~~~~~~~~~~~~~~
159495636oke     ~~~~~~~~~~~~~~~~~~~~~~~~~~~~~~~~~~~~~~~~~~~~~~~~~~~~~~~~~~~~~~~~~~~~~~~~~~~~~~~~~~~~~~~~~~
159497178oke     ~~~~~~~~~~~~~~~~~~~~~~~~~~~~~~~~~~~~~~~~~~~~~~~~~~~~~~~~~~~~~~~~~~~~~~~~~~~~~~~~~~~~~~~~~~
159498304oke     ~~~~~~~~~~~~~~~~~~~~~~~~~~~~~~~~~~~~~~~~~~~~~~~~~~~~~~~~~~~~~~~~~~~~~~~~~~~~~~~~~~~~~~~~~~
159496326oke     ~~~~~~~~~~~~~~~~~~~~~~~~~~~~~~~~~~~~~~~~~~~~~~~~~~~~~~~~~~~~~~~~~~~~~~~~~~~~~~~~~~~~~~~~~~
159495404oke     ~~~~~~~~~~~~~~~~~~~~~~~~~~~~~~~~~~~~~~~~~~~~~~~~~~~~~~~~~~~~~~~~~~~~~~~~~~~~~~~~~~~~~~~~~~
159493141oke     ~~~~~~~~~~~~~~~~~~~~~~~~~~~~~~~~~~~~~~~~~~~~~~~~~~~~~~~~~~~~~~~~~~~~~~~~~~~~~~~~~~~~~~~~~~
159493570oke     ~~~~~~~~~~~~~~~~~~~~~~~~~~~~~~~~~~~~~~~~~~~~~~~~~~~~~~~~~~~~~~~~~~~~~~~~~~~~~~~~~~~~~~~~~~
159434499oke     ~~~~~~~~~~~~~~~~~~~~~~~~~~~~~~~~~~~~~~~~~~~~~~~~~~~~~~~~~~~~~~~~~~~~~~~~~~~~~~~~~~~~~~~~~~
159493217oke     ~~~~~~~~~~~~~~~~~~~~~~~~~~~~~~~~~~~~~~~~~~~~~~~~~~~~~~~~~~~~~~~~~~~~~~~~~~~~~~~~~~~~~~~~~~
159493216oke     ~~~~~~~~~~~~~~~~~~~~~~~~~~~~~~~~~~~~~~~~~~~~~~~~~~~~~~~~~~~~~~~~~~~~~~~~~~~~~~~~~~~~~~~~~~
159493234oke     ~~~~~~~~~~~~~~~~~~~~~~~~~~~~~~~~~~~~~~~~~~~~~~~~~~~~~~~~~~~~~~~~~~~~~~~~~~~~~~~~~~~~~~~~~~
159492908oke     ~~~~~~~~~~~~~~~~~~~~~~~~~~~~~~~~~~~~~~~~~~~~~~~~~~~~~~~~~~~~~~~~~~~~~~~~~~~~~~~~~~~~~~~~~~
159491934oke     ~~~~~~~~~~~~~~~~~~~~~~~~~~~~~~~~~~~~~~~~~~~~~~~~~~~~~~~~~~~~~~~~~~~~~~~~~~~~~~~~~~~~~~~~~~
159492538        ~~~~~~~~~~~~~~~~~~~~~~~~~~~~~~~~~~~~~~~~~~~~~~~~~~~~~~~~~~~~~~~~~~~~~~~~~~~~~~~~~~~~~~~~~~
159498161        ~~~~~~~~~~~~~~~~~~~~~~~~~~~~~~~~~~~~~~~~~~~~~~~~~~~~~~~~~~~~~~~~~~~~~~~~~~~~~~~~~~~~~~~~~~
159493124        ~~~~~~~~~~~~~~~~~~~~~~~~~~~~~~~~~~~~~~~~~~~~~~~~~~~~~~~~~~~~~~~~~~~~~~~~~~~~~~~~~~~~~~~~~~
159493661        ~~~~~~~~~~~~~~~~~~~~~~~~~~~~~~~~~~~~~~~~~~~~~~~~~~~~~~~~~~~~~~~~~~~~~~~~~~~~~~~~~~~~~~~~~~
159494727        ~~~~~~~~~~~~~~~~~~~~~~~~~~~~~~~~~~~~~~~~~~~~~~~~~~~~~~~~~~~~~~~~~~~~~~~~~~~~~~~~~~~~~~~~~~
159496944        ~~~~~~~~~~~~~~~~~~~~~~~~~~~~~~~~~~~~~~~~~~~~~~~~~~~~~~~~~~~~~~~~~~~~~~~~~~~~~~~~~~~~~~~~~~
159494492        ~~~~~~~~~~~~~~~~~~~~~~~~~~~~~~~~~~~~~~~~~~~~~~~~~~~~~~~~~~~~~~~~~~~~~~~~~~~~~~~~~~~~~~~~~~
159496140        ~~~~~~~~~~~~~~~~~~~~~~~~~~~~~~~~~~~~~~~~~~~~~~~~~~~~~~~~~~~~~~~~~~~~~~~~~~~~~~~~~~~~~~~~~~
159497160        ~~~~~~~~~~~~~~~~~~~~~~~~~~~~~~~~~~~~~~~~~~~~~~~~~~~~~~~~~~~~~~~~~~~~~~~~~~~~~~~~~~~~~~~~~~
aaa07c02.g1      ~~~~~~~~~~~~~~~~~~~~~~~~~~~~~~~~~~~~~~~~~~~~~~~~~~~~~~~~~~~~~~~~~~~~~~~~~~~~~~~~~~~~~~~~~~

                         280       290       300       310       320       330       340       350       360
                 ....|....|....|....|....|....|....|....|....|....|....|....|....|....|....|....|....|....|
rsmitogen        aatgatttatctgtaaaaattattggtcatcaatgatattgaacttaagaattaggtgataatttaggtatagaatttgattcataaata
rscox2           aatgatttatctgtaaaaattattggtcatcaatgatattgaacttaagaattaggtgataatttaggtatagaatttgattcataaata
rstrna-his       ~~~~~~~~~~~~~~~~~~~~~~~~~~~~~~~~~~~~~~~~~~~~~~~~~~~~~~~~~~~~~~~~~~~~~~~~~~~~~~~~~~~~~~~~~~
rs16s            ~~~~~~~~~~~~~~~~~~~~~~~~~~~~~~~~~~~~~~~~~~~~~~~~~~~~~~~~~~~~~~~~~~~~~~~~~~~~~~~~~~~~~~~~~~
rsnad3           ~~~~~~~~~~~~~~~~~~~~~~~~~~~~~~~~~~~~~~~~~~~~~~~~~~~~~~~~~~~~~~~~~~~~~~~~~~~~~~~~~~~~~~~~~~
rstrna-leu       ~~~~~~~~~~~~~~~~~~~~~~~~~~~~~~~~~~~~~~~~~~~~~~~~~~~~~~~~~~~~~~~~~~~~~~~~~~~~~~~~~~~~~~~~~~
rscox3           ~~~~~~~~~~~~~~~~~~~~~~~~~~~~~~~~~~~~~~~~~~~~~~~~~~~~~~~~~~~~~~~~~~~~~~~~~~~~~~~~~~~~~~~~~~
rstrna-pro       ~~~~~~~~~~~~~~~~~~~~~~~~~~~~~~~~~~~~~~~~~~~~~~~~~~~~~~~~~~~~~~~~~~~~~~~~~~~~~~~~~~~~~~~~~~
rstrna-arg       ~~~~~~~~~~~~~~~~~~~~~~~~~~~~~~~~~~~~~~~~~~~~~~~~~~~~~~~~~~~~~~~~~~~~~~~~~~~~~~~~~~~~~~~~~~
rsnad6           ~~~~~~~~~~~~~~~~~~~~~~~~~~~~~~~~~~~~~~~~~~~~~~~~~~~~~~~~~~~~~~~~~~~~~~~~~~~~~~~~~~~~~~~~~~
rsnad4L          ~~~~~~~~~~~~~~~~~~~~~~~~~~~~~~~~~~~~~~~~~~~~~~~~~~~~~~~~~~~~~~~~~~~~~~~~~~~~~~~~~~~~~~~~~~
rstrna-val       ~~~~~~~~~~~~~~~~~~~~~~~~~~~~~~~~~~~~~~~~~~~~~~~~~~~~~~~~~~~~~~~~~~~~~~~~~~~~~~~~~~~~~~~~~~
rstrna-leu       ~~~~~~~~~~~~~~~~~~~~~~~~~~~~~~~~~~~~~~~~~~~~~~~~~~~~~~~~~~~~~~~~~~~~~~~~~~~~~~~~~~~~~~~~~~
159494961oke     AATGATTTATCTGTAAAAATTATTGGTCATCAATGATATTGAACTTAAGAATTAGGTGATAATTTAGGTATAGAATTTGATTCATAAATA
159496557oke     AATGATTTATCTGTAAAAATTATTGGTCATCAATGATATTGAACTTAAGAATTAGGTGATAATTTAGGTATAGAATTTGATTCATAAATA
159497093oke     ~~~~~~~~~~~~~~~~~~~~~~~~~~~~~~~~~~~~~~~~~~~~~~~~~~~~~~~~~~~~~~~~~~~~~~~~~~~~~~~~~~~~~~~~~~
159496622oke     ~~~~~~~~~~~~~~~~~~~~~~~~~~~~~~~~~~~~~~~~~~~~~~~~~~~~~~~~~~~~~~~~~~~~~~~~~~~~~~~~~~~~~~~~~~
159498117oke     ~~~~~~~~~~~~~~~~~~~~~~~~~~~~~~~~~~~~~~~~~~~~~~~~~~~~~~~~~~~~~~~~~~~~~~~~~~~~~~~~~~~~~~~~~~
159494290oke     ~~~~~~~~~~~~~~~~~~~~~~~~~~~~~~~~~~~~~~~~~~~~~~~~~~~~~~~~~~~~~~~~~~~~~~~~~~~~~~~~~~~~~~~~~~
159495636oke     ~~~~~~~~~~~~~~~~~~~~~~~~~~~~~~~~~~~~~~~~~~~~~~~~~~~~~~~~~~~~~~~~~~~~~~~~~~~~~~~~~~~~~~~~~~
159497178oke     ~~~~~~~~~~~~~~~~~~~~~~~~~~~~~~~~~~~~~~~~~~~~~~~~~~~~~~~~~~~~~~~~~~~~~~~~~~~~~~~~~~~~~~~~~~
159498304oke     ~~~~~~~~~~~~~~~~~~~~~~~~~~~~~~~~~~~~~~~~~~~~~~~~~~~~~~~~~~~~~~~~~~~~~~~~~~~~~~~~~~~~~~~~~~
159496326oke     ~~~~~~~~~~~~~~~~~~~~~~~~~~~~~~~~~~~~~~~~~~~~~~~~~~~~~~~~~~~~~~~~~~~~~~~~~~~~~~~~~~~~~~~~~~
159495404oke     ~~~~~~~~~~~~~~~~~~~~~~~~~~~~~~~~~~~~~~~~~~~~~~~~~~~~~~~~~~~~~~~~~~~~~~~~~~~~~~~~~~~~~~~~~~
159493141oke     ~~~~~~~~~~~~~~~~~~~~~~~~~~~~~~~~~~~~~~~~~~~~~~~~~~~~~~~~~~~~~~~~~~~~~~~~~~~~~~~~~~~~~~~~~~
159493570oke     ~~~~~~~~~~~~~~~~~~~~~~~~~~~~~~~~~~~~~~~~~~~~~~~~~~~~~~~~~~~~~~~~~~~~~~~~~~~~~~~~~~~~~~~~~~
159434499oke     ~~~~~~~~~~~~~~~~~~~~~~~~~~~~~~~~~~~~~~~~~~~~~~~~~~~~~~~~~~~~~~~~~~~~~~~~~~~~~~~~~~~~~~~~~~
159493217oke     ~~~~~~~~~~~~~~~~~~~~~~~~~~~~~~~~~~~~~~~~~~~~~~~~~~~~~~~~~~~~~~~~~~~~~~~~~~~~~~~~~~~~~~~~~~
159493216oke     ~~~~~~~~~~~~~~~~~~~~~~~~~~~~~~~~~~~~~~~~~~~~~~~~~~~~~~~~~~~~~~~~~~~~~~~~~~~~~~~~~~~~~~~~~~
159493234oke     ~~~~~~~~~~~~~~~~~~~~~~~~~~~~~~~~~~~~~~~~~~~~~~~~~~~~~~~~~~~~~~~~~~~~~~~~~~~~~~~~~~~~~~~~~~
159492908oke     ~~~~~~~~~~~~~~~~~~~~~~~~~~~~~~~~~~~~~~~~~~~~~~~~~~~~~~~~~~~~~~~~~~~~~~~~~~~~~~~~~~~~~~~~~~
159491934oke     ~~~~~~~~~~~~~~~~~~~~~~~~~~~~~~~~~~~~~~~~~~~~~~~~~~~~~~~~~~~~~~~~~~~~~~~~~~~~~~~~~~~~~~~~~~
159492538        ~~~~~~~~~~~~~~~~~~~~~~~~~~~~~~~~~~~~~~~~~~~~~~~~~~~~~~~~~~~~~~~~~~~~~~~~~~~~~~~~~~~~~~~~~~
159498161        ~~~~~~~~~~~~~~~~~~~~~~~~~~~~~~~~~~~~~~~~~~~~~~~~~~~~~~~~~~~~~~~~~~~~~~~~~~~~~~~~~~~~~~~~~~
159493124        ~~~~~~~~~~~~~~~~~~~~~~~~~~~~~~~~~~~~~~~~~~~~~~~~~~~~~~~~~~~~~~~~~~~~~~~~~~~~~~~~~~~~~~~~~~
159493661        ~~~~~~~~~~~~~~~~~~~~~~~~~~~~~~~~~~~~~~~~~~~~~~~~~~~~~~~~~~~~~~~~~~~~~~~~~~~~~~~~~~~~~~~~~~
159494727        ~~~~~~~~~~~~~~~~~~~~~~~~~~~~~~~~~~~~~~~~~~~~~~~~~~~~~~~~~~~~~~~~~~~~~~~~~~~~~~~~~~~~~~~~~~
159496944        ~~~~~~~~~~~~~~~~~~~~~~~~~~~~~~~~~~~~~~~~~~~~~~~~~~~~~~~~~~~~~~~~~~~~~~~~~~~~~~~~~~~~~~~~~~
159494492        ~~~~~~~~~~~~~~~~~~~~~~~~~~~~~~~~~~~~~~~~~~~~~~~~~~~~~~~~~~~~~~~~~~~~~~~~~~~~~~~~~~~~~~~~~~
159496140        ~~~~~~~~~~~~~~~~~~~~~~~~~~~~~~~~~~~~~~~~~~~~~~~~~~~~~~~~~~~~~~~~~~~~~~~~~~~~~~~~~~~~~~~~~~
159497160        ~~~~~~~~~~~~~~~~~~~~~~~~~~~~~~~~~~~~~~~~~~~~~~~~~~~~~~~~~~~~~~~~~~~~~~~~~~~~~~~~~~~~~~~~~~
aaa07c02.g1      ~~~~~~~~~~~~~~~~~~~~~~~~~~~~~~~~~~~~~~~~~~~~~~~~~~~~~~~~~~~~~~~~~~~~~~~~~~~~~~~~~~~~~~~~~~

                         370       380       390       400       410       420       430       440       450
                 ....|....|....|....|....|....|....|....|....|....|....|....|....|....|....|....|....|....|
rsmitogen        aaatctaatgattaattgattttaggtgattaacgtttattagaggtagataatcgtttaattattccaaaggatatattgattcgtttt
rscox2           aaatctaatgattaattgattttaggtgattaacgtttattagaggtagataatcgtttaattattccaaaggatatattgattcgtttt
rstrna-his       ~~~~~~~~~~~~~~~~~~~~~~~~~~~~~~~~~~~~~~~~~~~~~~~~~~~~~~~~~~~~~~~~~~~~~~~~~~~~~~~~~~~~~~~~~~
rs16s            ~~~~~~~~~~~~~~~~~~~~~~~~~~~~~~~~~~~~~~~~~~~~~~~~~~~~~~~~~~~~~~~~~~~~~~~~~~~~~~~~~~~~~~~~~~
rsnad3           ~~~~~~~~~~~~~~~~~~~~~~~~~~~~~~~~~~~~~~~~~~~~~~~~~~~~~~~~~~~~~~~~~~~~~~~~~~~~~~~~~~~~~~~~~~
rstrna-leu       ~~~~~~~~~~~~~~~~~~~~~~~~~~~~~~~~~~~~~~~~~~~~~~~~~~~~~~~~~~~~~~~~~~~~~~~~~~~~~~~~~~~~~~~~~~
rscox3           ~~~~~~~~~~~~~~~~~~~~~~~~~~~~~~~~~~~~~~~~~~~~~~~~~~~~~~~~~~~~~~~~~~~~~~~~~~~~~~~~~~~~~~~~~~
rstrna-pro       ~~~~~~~~~~~~~~~~~~~~~~~~~~~~~~~~~~~~~~~~~~~~~~~~~~~~~~~~~~~~~~~~~~~~~~~~~~~~~~~~~~~~~~~~~~
rstrna-arg       ~~~~~~~~~~~~~~~~~~~~~~~~~~~~~~~~~~~~~~~~~~~~~~~~~~~~~~~~~~~~~~~~~~~~~~~~~~~~~~~~~~~~~~~~~~
rsnad6           ~~~~~~~~~~~~~~~~~~~~~~~~~~~~~~~~~~~~~~~~~~~~~~~~~~~~~~~~~~~~~~~~~~~~~~~~~~~~~~~~~~~~~~~~~~
rsnad4L          ~~~~~~~~~~~~~~~~~~~~~~~~~~~~~~~~~~~~~~~~~~~~~~~~~~~~~~~~~~~~~~~~~~~~~~~~~~~~~~~~~~~~~~~~~~
rstrna-val       ~~~~~~~~~~~~~~~~~~~~~~~~~~~~~~~~~~~~~~~~~~~~~~~~~~~~~~~~~~~~~~~~~~~~~~~~~~~~~~~~~~~~~~~~~~
rstrna-leu       ~~~~~~~~~~~~~~~~~~~~~~~~~~~~~~~~~~~~~~~~~~~~~~~~~~~~~~~~~~~~~~~~~~~~~~~~~~~~~~~~~~~~~~~~~~
159494961oke     AAATCTAATGATTAATTGATTTTAGGTGATTAACGTTTATTAGAGGTAGATAATCGTTTAATTATTCCAAAGGATATATTGATTCGTTTT
159496557oke     AAATCTAATGATTAATTGATTTTAGGTGATTAACGTTTATTAGAGGTAGATAATCGTTTAATTATTCCAAAGGATATATTGATTCGTTTT
159497093oke     ~~~~~~~~~~~~~~~~~~~~~~~~~~~~~~~~~~~~~~~~~~~~~~~~~~~~~~~~~~~~~~~~~~~~~~~~~~~~~~~~~~~~~~~~~~
159496622oke     ~~~~~~~~~~~~~~~~~~~~~~~~~~~~~~~~~~~~~~~~~~~~~~~~~~~~~~~~~~~~~~~~~~~~~~~~~~~~~~~~~~~~~~~~~~
159498117oke     ~~~~~~~~~~~~~~~~~~~~~~~~~~~~~~~~~~~~~~~~~~~~~~~~~~~~~~~~~~~~~~~~~~~~~~~~~~~~~~~~~~~~~~~~~~
159494290oke     ~~~~~~~~~~~~~~~~~~~~~~~~~~~~~~~~~~~~~~~~~~~~~~~~~~~~~~~~~~~~~~~~~~~~~~~~~~~~~~~~~~~~~~~~~~
159495636oke     ~~~~~~~~~~~~~~~~~~~~~~~~~~~~~~~~~~~~~~~~~~~~~~~~~~~~~~~~~~~~~~~~~~~~~~~~~~~~~~~~~~~~~~~~~~
159497178oke     ~~~~~~~~~~~~~~~~~~~~~~~~~~~~~~~~~~~~~~~~~~~~~~~~~~~~~~~~~~~~~~~~~~~~~~~~~~~~~~~~~~~~~~~~~~
159498304oke     ~~~~~~~~~~~~~~~~~~~~~~~~~~~~~~~~~~~~~~~~~~~~~~~~~~~~~~~~~~~~~~~~~~~~~~~~~~~~~~~~~~~~~~~~~~
159496326oke     ~~~~~~~~~~~~~~~~~~~~~~~~~~~~~~~~~~~~~~~~~~~~~~~~~~~~~~~~~~~~~~~~~~~~~~~~~~~~~~~~~~~~~~~~~~
159495404oke     ~~~~~~~~~~~~~~~~~~~~~~~~~~~~~~~~~~~~~~~~~~~~~~~~~~~~~~~~~~~~~~~~~~~~~~~~~~~~~~~~~~~~~~~~~~
159493141oke     ~~~~~~~~~~~~~~~~~~~~~~~~~~~~~~~~~~~~~~~~~~~~~~~~~~~~~~~~~~~~~~~~~~~~~~~~~~~~~~~~~~~~~~~~~~
159493570oke     ~~~~~~~~~~~~~~~~~~~~~~~~~~~~~~~~~~~~~~~~~~~~~~~~~~~~~~~~~~~~~~~~~~~~~~~~~~~~~~~~~~~~~~~~~~
159434499oke     ~~~~~~~~~~~~~~~~~~~~~~~~~~~~~~~~~~~~~~~~~~~~~~~~~~~~~~~~~~~~~~~~~~~~~~~~~~~~~~~~~~~~~~~~~~
159493217oke     ~~~~~~~~~~~~~~~~~~~~~~~~~~~~~~~~~~~~~~~~~~~~~~~~~~~~~~~~~~~~~~~~~~~~~~~~~~~~~~~~~~~~~~~~~~
159493216oke     ~~~~~~~~~~~~~~~~~~~~~~~~~~~~~~~~~~~~~~~~~~~~~~~~~~~~~~~~~~~~~~~~~~~~~~~~~~~~~~~~~~~~~~~~~~
159493234oke     ~~~~~~~~~~~~~~~~~~~~~~~~~~~~~~~~~~~~~~~~~~~~~~~~~~~~~~~~~~~~~~~~~~~~~~~~~~~~~~~~~~~~~~~~~~
159492908oke     ~~~~~~~~~~~~~~~~~~~~~~~~~~~~~~~~~~~~~~~~~~~~~~~~~~~~~~~~~~~~~~~~~~~~~~~~~~~~~~~~~~~~~~~~~~
159491934oke     ~~~~~~~~~~~~~~~~~~~~~~~~~~~~~~~~~~~~~~~~~~~~~~~~~~~~~~~~~~~~~~~~~~~~~~~~~~~~~~~~~~~~~~~~~~
159492538        ~~~~~~~~~~~~~~~~~~~~~~~~~~~~~~~~~~~~~~~~~~~~~~~~~~~~~~~~~~~~~~~~~~~~~~~~~~~~~~~~~~~~~~~~~~
159498161        ~~~~~~~~~~~~~~~~~~~~~~~~~~~~~~~~~~~~~~~~~~~~~~~~~~~~~~~~~~~~~~~~~~~~~~~~~~~~~~~~~~~~~~~~~~
159493124        ~~~~~~~~~~~~~~~~~~~~~~~~~~~~~~~~~~~~~~~~~~~~~~~~~~~~~~~~~~~~~~~~~~~~~~~~~~~~~~~~~~~~~~~~~~
159493661        ~~~~~~~~~~~~~~~~~~~~~~~~~~~~~~~~~~~~~~~~~~~~~~~~~~~~~~~~~~~~~~~~~~~~~~~~~~~~~~~~~~~~~~~~~~
159494727        ~~~~~~~~~~~~~~~~~~~~~~~~~~~~~~~~~~~~~~~~~~~~~~~~~~~~~~~~~~~~~~~~~~~~~~~~~~~~~~~~~~~~~~~~~~
159496944        ~~~~~~~~~~~~~~~~~~~~~~~~~~~~~~~~~~~~~~~~~~~~~~~~~~~~~~~~~~~~~~~~~~~~~~~~~~~~~~~~~~~~~~~~~~
159494492        ~~~~~~~~~~~~~~~~~~~~~~~~~~~~~~~~~~~~~~~~~~~~~~~~~~~~~~~~~~~~~~~~~~~~~~~~~~~~~~~~~~~~~~~~~~
159496140        ~~~~~~~~~~~~~~~~~~~~~~~~~~~~~~~~~~~~~~~~~~~~~~~~~~~~~~~~~~~~~~~~~~~~~~~~~~~~~~~~~~~~~~~~~~
159497160        ~~~~~~~~~~~~~~~~~~~~~~~~~~~~~~~~~~~~~~~~~~~~~~~~~~~~~~~~~~~~~~~~~~~~~~~~~~~~~~~~~~~~~~~~~~
aaa07c02.g1      ~~~~~~~~~~~~~~~~~~~~~~~~~~~~~~~~~~~~~~~~~~~~~~~~~~~~~~~~~~~~~~~~~~~~~~~~~~~~~~~~~~~~~~~~~~

                         460       470       480       490       500       510       520       530       540
                 ....|....|....|....|....|....|....|....|....|....|....|....|....|....|....|....|....|....|
rsmitogen        gttataacttctactgatgttattcattcttgggctttacctatatattttattaaattagatgtaatgagaggtataatatcagttttt
rscox2           gttataacttctactgatgttattcattcttgggctttacctatatattttattaaattagatgtaatgagaggtataatatcagttttt
rstrna-his       ~~~~~~~~~~~~~~~~~~~~~~~~~~~~~~~~~~~~~~~~~~~~~~~~~~~~~~~~~~~~~~~~~~~~~~~~~~~~~~~~~~~~~~~~~~
rs16s            ~~~~~~~~~~~~~~~~~~~~~~~~~~~~~~~~~~~~~~~~~~~~~~~~~~~~~~~~~~~~~~~~~~~~~~~~~~~~~~~~~~~~~~~~~~
rsnad3           ~~~~~~~~~~~~~~~~~~~~~~~~~~~~~~~~~~~~~~~~~~~~~~~~~~~~~~~~~~~~~~~~~~~~~~~~~~~~~~~~~~~~~~~~~~
rstrna-leu       ~~~~~~~~~~~~~~~~~~~~~~~~~~~~~~~~~~~~~~~~~~~~~~~~~~~~~~~~~~~~~~~~~~~~~~~~~~~~~~~~~~~~~~~~~~
rscox3           ~~~~~~~~~~~~~~~~~~~~~~~~~~~~~~~~~~~~~~~~~~~~~~~~~~~~~~~~~~~~~~~~~~~~~~~~~~~~~~~~~~~~~~~~~~
rstrna-pro       ~~~~~~~~~~~~~~~~~~~~~~~~~~~~~~~~~~~~~~~~~~~~~~~~~~~~~~~~~~~~~~~~~~~~~~~~~~~~~~~~~~~~~~~~~~
rstrna-arg       ~~~~~~~~~~~~~~~~~~~~~~~~~~~~~~~~~~~~~~~~~~~~~~~~~~~~~~~~~~~~~~~~~~~~~~~~~~~~~~~~~~~~~~~~~~
rsnad6           ~~~~~~~~~~~~~~~~~~~~~~~~~~~~~~~~~~~~~~~~~~~~~~~~~~~~~~~~~~~~~~~~~~~~~~~~~~~~~~~~~~~~~~~~~~
rsnad4L          ~~~~~~~~~~~~~~~~~~~~~~~~~~~~~~~~~~~~~~~~~~~~~~~~~~~~~~~~~~~~~~~~~~~~~~~~~~~~~~~~~~~~~~~~~~
rstrna-val       ~~~~~~~~~~~~~~~~~~~~~~~~~~~~~~~~~~~~~~~~~~~~~~~~~~~~~~~~~~~~~~~~~~~~~~~~~~~~~~~~~~~~~~~~~~
rstrna-leu       ~~~~~~~~~~~~~~~~~~~~~~~~~~~~~~~~~~~~~~~~~~~~~~~~~~~~~~~~~~~~~~~~~~~~~~~~~~~~~~~~~~~~~~~~~~
159494961oke     GTTATAACTTCTACTGATGTTATTCATTCTTGGGCTTTACCTATATATTTTATTAAATTAGATGTAATGAGAGGTATAATATCAGTTTTT
159496557oke     GTTATAACTTCTACTGATGTTATTCATTCTTGGGCTTTACCTATATATTTTATTAAATTAGATGTAATGAGAGGTATAATATCAGTTTTT
159497093oke     ~~~~~~~~~~~~~~~~~~~~~~~~~~~~~~~~~~~~~~~~~~~~~~~~~~~~~~~~~~~~~~~~~~~~~~~~~~~~~~~~~~~~~~~~~~
159496622oke     ~~~~~~~~~~~~~~~~~~~~~~~~~~~~~~~~~~~~~~~~~~~~~~~~~~~~~~~~~~~~~~~~~~~~~~~~~~~~~~~~~~~~~~~~~~
159498117oke     ~~~~~~~~~~~~~~~~~~~~~~~~~~~~~~~~~~~~~~~~~~~~~~~~~~~~~~~~~~~~~~~~~~~~~~~~~~~~~~~~~~~~~~~~~~
159494290oke     ~~~~~~~~~~~~~~~~~~~~~~~~~~~~~~~~~~~~~~~~~~~~~~~~~~~~~~~~~~~~~~~~~~~~~~~~~~~~~~~~~~~~~~~~~~
159495636oke     ~~~~~~~~~~~~~~~~~~~~~~~~~~~~~~~~~~~~~~~~~~~~~~~~~~~~~~~~~~~~~~~~~~~~~~~~~~~~~~~~~~~~~~~~~~
159497178oke     ~~~~~~~~~~~~~~~~~~~~~~~~~~~~~~~~~~~~~~~~~~~~~~~~~~~~~~~~~~~~~~~~~~~~~~~~~~~~~~~~~~~~~~~~~~
159498304oke     ~~~~~~~~~~~~~~~~~~~~~~~~~~~~~~~~~~~~~~~~~~~~~~~~~~~~~~~~~~~~~~~~~~~~~~~~~~~~~~~~~~~~~~~~~~
159496326oke     ~~~~~~~~~~~~~~~~~~~~~~~~~~~~~~~~~~~~~~~~~~~~~~~~~~~~~~~~~~~~~~~~~~~~~~~~~~~~~~~~~~~~~~~~~~
159495404oke     ~~~~~~~~~~~~~~~~~~~~~~~~~~~~~~~~~~~~~~~~~~~~~~~~~~~~~~~~~~~~~~~~~~~~~~~~~~~~~~~~~~~~~~~~~~
159493141oke     ~~~~~~~~~~~~~~~~~~~~~~~~~~~~~~~~~~~~~~~~~~~~~~~~~~~~~~~~~~~~~~~~~~~~~~~~~~~~~~~~~~~~~~~~~~
159493570oke     ~~~~~~~~~~~~~~~~~~~~~~~~~~~~~~~~~~~~~~~~~~~~~~~~~~~~~~~~~~~~~~~~~~~~~~~~~~~~~~~~~~~~~~~~~~
159434499oke     ~~~~~~~~~~~~~~~~~~~~~~~~~~~~~~~~~~~~~~~~~~~~~~~~~~~~~~~~~~~~~~~~~~~~~~~~~~~~~~~~~~~~~~~~~~
159493217oke     ~~~~~~~~~~~~~~~~~~~~~~~~~~~~~~~~~~~~~~~~~~~~~~~~~~~~~~~~~~~~~~~~~~~~~~~~~~~~~~~~~~~~~~~~~~
159493216oke     ~~~~~~~~~~~~~~~~~~~~~~~~~~~~~~~~~~~~~~~~~~~~~~~~~~~~~~~~~~~~~~~~~~~~~~~~~~~~~~~~~~~~~~~~~~
159493234oke     ~~~~~~~~~~~~~~~~~~~~~~~~~~~~~~~~~~~~~~~~~~~~~~~~~~~~~~~~~~~~~~~~~~~~~~~~~~~~~~~~~~~~~~~~~~
159492908oke     ~~~~~~~~~~~~~~~~~~~~~~~~~~~~~~~~~~~~~~~~~~~~~~~~~~~~~~~~~~~~~~~~~~~~~~~~~~~~~~~~~~~~~~~~~~
159491934oke     ~~~~~~~~~~~~~~~~~~~~~~~~~~~~~~~~~~~~~~~~~~~~~~~~~~~~~~~~~~~~~~~~~~~~~~~~~~~~~~~~~~~~~~~~~~
159492538        ~~~~~~~~~~~~~~~~~~~~~~~~~~~~~~~~~~~~~~~~~~~~~~~~~~~~~~~~~~~~~~~~~~~~~~~~~~~~~~~~~~~~~~~~~~
159498161        ~~~~~~~~~~~~~~~~~~~~~~~~~~~~~~~~~~~~~~~~~~~~~~~~~~~~~~~~~~~~~~~~~~~~~~~~~~~~~~~~~~~~~~~~~~
159493124        ~~~~~~~~~~~~~~~~~~~~~~~~~~~~~~~~~~~~~~~~~~~~~~~~~~~~~~~~~~~~~~~~~~~~~~~~~~~~~~~~~~~~~~~~~~
159493661        ~~~~~~~~~~~~~~~~~~~~~~~~~~~~~~~~~~~~~~~~~~~~~~~~~~~~~~~~~~~~~~~~~~~~~~~~~~~~~~~~~~~~~~~~~~
159494727        ~~~~~~~~~~~~~~~~~~~~~~~~~~~~~~~~~~~~~~~~~~~~~~~~~~~~~~~~~~~~~~~~~~~~~~~~~~~~~~~~~~~~~~~~~~
159496944        ~~~~~~~~~~~~~~~~~~~~~~~~~~~~~~~~~~~~~~~~~~~~~~~~~~~~~~~~~~~~~~~~~~~~~~~~~~~~~~~~~~~~~~~~~~
159494492        ~~~~~~~~~~~~~~~~~~~~~~~~~~~~~~~~~~~~~~~~~~~~~~~~~~~~~~~~~~~~~~~~~~~~~~~~~~~~~~~~~~~~~~~~~~
159496140        ~~~~~~~~~~~~~~~~~~~~~~~~~~~~~~~~~~~~~~~~~~~~~~~~~~~~~~~~~~~~~~~~~~~~~~~~~~~~~~~~~~~~~~~~~~
159497160        ~~~~~~~~~~~~~~~~~~~~~~~~~~~~~~~~~~~~~~~~~~~~~~~~~~~~~~~~~~~~~~~~~~~~~~~~~~~~~~~~~~~~~~~~~~
aaa07c02.g1      ~~~~~~~~~~~~~~~~~~~~~~~~~~~~~~~~~~~~~~~~~~~~~~~~~~~~~~~~~~~~~~~~~~~~~~~~~~~~~~~~~~~~~~~~~~

                         550       560       570       580       590       600       610       620       630
                 ....|....|....|....|....|....|....|....|....|....|....|....|....|....|....|....|....|....|
rsmitogen        aattatagttttaatattattggattattttaaggtcaatgttctgaaatttgtggaactaatcattcttttataccagttgtggttgag
rscox2           aattatagttttaatattattggattattttaaggtcaatgttctgaaatttgtggaactaatcattcttttataccagttgtggttgag
rstrna-his       ~~~~~~~~~~~~~~~~~~~~~~~~~~~~~~~~~~~~~~~~~~~~~~~~~~~~~~~~~~~~~~~~~~~~~~~~~~~~~~~~~~~~~~~~~~
rs16s            ~~~~~~~~~~~~~~~~~~~~~~~~~~~~~~~~~~~~~~~~~~~~~~~~~~~~~~~~~~~~~~~~~~~~~~~~~~~~~~~~~~~~~~~~~~
rsnad3           ~~~~~~~~~~~~~~~~~~~~~~~~~~~~~~~~~~~~~~~~~~~~~~~~~~~~~~~~~~~~~~~~~~~~~~~~~~~~~~~~~~~~~~~~~~
rstrna-leu       ~~~~~~~~~~~~~~~~~~~~~~~~~~~~~~~~~~~~~~~~~~~~~~~~~~~~~~~~~~~~~~~~~~~~~~~~~~~~~~~~~~~~~~~~~~
```

```
rscox3        ~~~~~~~~~~~~~~~~~~~~~~~~~~~~~~~~~~~~~~~~~~~~~~~~~~~~~~~~~~~~~~~~~~~~~~~~~~~~~~~~~~~~~~~~~~
rstrna-pro    ~~~~~~~~~~~~~~~~~~~~~~~~~~~~~~~~~~~~~~~~~~~~~~~~~~~~~~~~~~~~~~~~~~~~~~~~~~~~~~~~~~~~~~~~~~
rstrna-arg    ~~~~~~~~~~~~~~~~~~~~~~~~~~~~~~~~~~~~~~~~~~~~~~~~~~~~~~~~~~~~~~~~~~~~~~~~~~~~~~~~~~~~~~~~~~
rsnad6        ~~~~~~~~~~~~~~~~~~~~~~~~~~~~~~~~~~~~~~~~~~~~~~~~~~~~~~~~~~~~~~~~~~~~~~~~~~~~~~~~~~~~~~~~~~
rsnad4L       ~~~~~~~~~~~~~~~~~~~~~~~~~~~~~~~~~~~~~~~~~~~~~~~~~~~~~~~~~~~~~~~~~~~~~~~~~~~~~~~~~~~~~~~~~~
rstrna-val    ~~~~~~~~~~~~~~~~~~~~~~~~~~~~~~~~~~~~~~~~~~~~~~~~~~~~~~~~~~~~~~~~~~~~~~~~~~~~~~~~~~~~~~~~~~
rstrna-leu    ~~~~~~~~~~~~~~~~~~~~~~~~~~~~~~~~~~~~~~~~~~~~~~~~~~~~~~~~~~~~~~~~~~~~~~~~~~~~~~~~~~~~~~~~~~
159494961oke  AATTACCCGGCC~~~
159496557oke  AATTATAGTTTTAATATTATTGGATTATTTTAAGGTCAATGTTCTGAAATTTGTGGAACTCCCGGCC~~~~
159497093oke  ~~~TATAGTTTTAATATTATTGGATTATTTTAAGGTCAAGGTTTTGAAATTGGTGGAATTAATCATTCTTTTATACCAGTGGTGGTGGAG
159496622oke  ~~~~~~~~~~~~~~~~~~~~~~~~~~~~~~~~~~~~~~~~~~~~~~~~~~~~~~~~~~~~~~~~~~~~~~~~~~~~~~~~~~~~~~~~~~
159498117oke  ~~~~~~~~~~~~~~~~~~~~~~~~~~~~~~~~~~~~~~~~~~~~~~~~~~~~~~~~~~~~~~~~~~~~~~~~~~~~~~~~~~~~~~~~~~
159494290oke  ~~~~~~~~~~~~~~~~~~~~~~~~~~~~~~~~~~~~~~~~~~~~~~~~~~~~~~~~~~~~~~~~~~~~~~~~~~~~~~~~~~~~~~~~~~
159495636oke  ~~~~~~~~~~~~~~~~~~~~~~~~~~~~~~~~~~~~~~~~~~~~~~~~~~~~~~~~~~~~~~~~~~~~~~~~~~~~~~~~~~~~~~~~~~
159497178oke  ~~~~~~~~~~~~~~~~~~~~~~~~~~~~~~~~~~~~~~~~~~~~~~~~~~~~~~~~~~~~~~~~~~~~~~~~~~~~~~~~~~~~~~~~~~
159498304oke  ~~~~~~~~~~~~~~~~~~~~~~~~~~~~~~~~~~~~~~~~~~~~~~~~~~~~~~~~~~~~~~~~~~~~~~~~~~~~~~~~~~~~~~~~~~
159496326oke  ~~~~~~~~~~~~~~~~~~~~~~~~~~~~~~~~~~~~~~~~~~~~~~~~~~~~~~~~~~~~~~~~~~~~~~~~~~~~~~~~~~~~~~~~~~
159495404oke  ~~~~~~~~~~~~~~~~~~~~~~~~~~~~~~~~~~~~~~~~~~~~~~~~~~~~~~~~~~~~~~~~~~~~~~~~~~~~~~~~~~~~~~~~~~
159493141oke  ~~~~~~~~~~~~~~~~~~~~~~~~~~~~~~~~~~~~~~~~~~~~~~~~~~~~~~~~~~~~~~~~~~~~~~~~~~~~~~~~~~~~~~~~~~
159493570oke  ~~~~~~~~~~~~~~~~~~~~~~~~~~~~~~~~~~~~~~~~~~~~~~~~~~~~~~~~~~~~~~~~~~~~~~~~~~~~~~~~~~~~~~~~~~
159493499oke  ~~~~~~~~~~~~~~~~~~~~~~~~~~~~~~~~~~~~~~~~~~~~~~~~~~~~~~~~~~~~~~~~~~~~~~~~~~~~~~~~~~~~~~~~~~
159493217oke  ~~~~~~~~~~~~~~~~~~~~~~~~~~~~~~~~~~~~~~~~~~~~~~~~~~~~~~~~~~~~~~~~~~~~~~~~~~~~~~~~~~~~~~~~~~
159493216oke  ~~~~~~~~~~~~~~~~~~~~~~~~~~~~~~~~~~~~~~~~~~~~~~~~~~~~~~~~~~~~~~~~~~~~~~~~~~~~~~~~~~~~~~~~~~
159493234oke  ~~~~~~~~~~~~~~~~~~~~~~~~~~~~~~~~~~~~~~~~~~~~~~~~~~~~~~~~~~~~~~~~~~~~~~~~~~~~~~~~~~~~~~~~~~
159492908oke  ~~~~~~~~~~~~~~~~~~~~~~~~~~~~~~~~~~~~~~~~~~~~~~~~~~~~~~~~~~~~~~~~~~~~~~~~~~~~~~~~~~~~~~~~~~
159491934oke  ~~~~~~~~~~~~~~~~~~~~~~~~~~~~~~~~~~~~~~~~~~~~~~~~~~~~~~~~~~~~~~~~~~~~~~~~~~~~~~~~~~~~~~~~~~
159492538     ~~~~~~~~~~~~~~~~~~~~~~~~~~~~~~~~~~~~~~~~~~~~~~~~~~~~~~~~~~~~~~~~~~~~~~~~~~~~~~~~~~~~~~~~~~
159498161     ~~~~~~~~~~~~~~~~~~~~~~~~~~~~~~~~~~~~~~~~~~~~~~~~~~~~~~~~~~~~~~~~~~~~~~~~~~~~~~~~~~~~~~~~~~
159493124     ~~~~~~~~~~~~~~~~~~~~~~~~~~~~~~~~~~~~~~~~~~~~~~~~~~~~~~~~~~~~~~~~~~~~~~~~~~~~~~~~~~~~~~~~~~
159493661     ~~~~~~~~~~~~~~~~~~~~~~~~~~~~~~~~~~~~~~~~~~~~~~~~~~~~~~~~~~~~~~~~~~~~~~~~~~~~~~~~~~~~~~~~~~
159494727     ~~~~~~~~~~~~~~~~~~~~~~~~~~~~~~~~~~~~~~~~~~~~~~~~~~~~~~~~~~~~~~~~~~~~~~~~~~~~~~~~~~~~~~~~~~
159496944     ~~~~~~~~~~~~~~~~~~~~~~~~~~~~~~~~~~~~~~~~~~~~~~~~~~~~~~~~~~~~~~~~~~~~~~~~~~~~~~~~~~~~~~~~~~
159494492     ~~~~~~~~~~~~~~~~~~~~~~~~~~~~~~~~~~~~~~~~~~~~~~~~~~~~~~~~~~~~~~~~~~~~~~~~~~~~~~~~~~~~~~~~~~
159496140     ~~~~~~~~~~~~~~~~~~~~~~~~~~~~~~~~~~~~~~~~~~~~~~~~~~~~~~~~~~~~~~~~~~~~~~~~~~~~~~~~~~~~~~~~~~
159497160     ~~~~~~~~~~~~~~~~~~~~~~~~~~~~~~~~~~~~~~~~~~~~~~~~~~~~~~~~~~~~~~~~~~~~~~~~~~~~~~~~~~~~~~~~~~
aaa07c02.g1   ~~~~~~~~~~~~~~~~~~~~~~~~~~~~~~~~~~~~~~~~~~~~~~~~~~~~~~~~~~~~~~~~~~~~~~~~~~~~~~~~~~~~~~~~~~

                      640       650       660       670       680       690       700       710       720
              ....|....|....|....|....|....|....|....|....|....|....|....|....|....|....|....|....|....|
rsmitogen     gttactttatttaatttttttaaaaattaaatatttttattaaagcttttttatttttataaatttttgtttgtggaacataaagttaaa
rscox2        gttactttatttaatttttttaaaaattaaatatttttatta~~~~~~
rstrna-his    ~~~~~~~~~~~~~~~~~~~~~~~~~~~~~~~~~~~~~~~~~~aagcttttttatttttataaatttttgtttgtggaacataaagttaaa
rs16s         ~~~~~~~~~~~~~~~~~~~~~~~~~~~~~~~~~~~~~~~~~~~~~~~~~~~~~~~~~~~~~~~~~~~~~~~~~~~~~~~~~~~~~~~~~~
rsnad3        ~~~~~~~~~~~~~~~~~~~~~~~~~~~~~~~~~~~~~~~~~~~~~~~~~~~~~~~~~~~~~~~~~~~~~~~~~~~~~~~~~~~~~~~~~~
rstrna-leu    ~~~~~~~~~~~~~~~~~~~~~~~~~~~~~~~~~~~~~~~~~~~~~~~~~~~~~~~~~~~~~~~~~~~~~~~~~~~~~~~~~~~~~~~~~~
rscox3        ~~~~~~~~~~~~~~~~~~~~~~~~~~~~~~~~~~~~~~~~~~~~~~~~~~~~~~~~~~~~~~~~~~~~~~~~~~~~~~~~~~~~~~~~~~
rstrna-pro    ~~~~~~~~~~~~~~~~~~~~~~~~~~~~~~~~~~~~~~~~~~~~~~~~~~~~~~~~~~~~~~~~~~~~~~~~~~~~~~~~~~~~~~~~~~
rstrna-arg    ~~~~~~~~~~~~~~~~~~~~~~~~~~~~~~~~~~~~~~~~~~~~~~~~~~~~~~~~~~~~~~~~~~~~~~~~~~~~~~~~~~~~~~~~~~
rsnad6        ~~~~~~~~~~~~~~~~~~~~~~~~~~~~~~~~~~~~~~~~~~~~~~~~~~~~~~~~~~~~~~~~~~~~~~~~~~~~~~~~~~~~~~~~~~
rsnad4L       ~~~~~~~~~~~~~~~~~~~~~~~~~~~~~~~~~~~~~~~~~~~~~~~~~~~~~~~~~~~~~~~~~~~~~~~~~~~~~~~~~~~~~~~~~~
rstrna-val    ~~~~~~~~~~~~~~~~~~~~~~~~~~~~~~~~~~~~~~~~~~~~~~~~~~~~~~~~~~~~~~~~~~~~~~~~~~~~~~~~~~~~~~~~~~
rstrna-leu    ~~~~~~~~~~~~~~~~~~~~~~~~~~~~~~~~~~~~~~~~~~~~~~~~~~~~~~~~~~~~~~~~~~~~~~~~~~~~~~~~~~~~~~~~~~
159494961oke
159496557oke
159497093oke  GTTACTTTATTTAATTTTTTTAAAAATAAAATATTTTTATTAAAGCTTTTTTATTTTTATAAATTTTTGTTTGTGGAACATAAAGTTAAA
159496622oke  ~~~~~~~~~~~~~~~~~~~~~~~~~ATTAAATATTTTTATTAAAGCTTTTTTATTTTTATAAAATTTTGTTTGTGGAACATAAAGTTAAA
159498117oke  ~~~~~~~~~~~~~~~~~~~~~~~~~~~~~~~~~~~~~~~~~~~~~~~~~~~~~~~~~~~~~~~~~~~~~~~~~~~~~~~~~~~~~~~~GG
159494290oke  ~~~~~~~~~~~~~~~~~~~~~~~~~~~~~~~~~~~~~~~~~~~~~~~~~~~~~~~~~~~~~~~~~~~~~~~~~~~~~~~~~~~~~~~~GG
159495636oke  ~~~~~~~~~~~~~~~~~~~~~~~~~~~~~~~~~~~~~~~~~~~~~~~~~~~~~~~~~~~~~~~~~~~~~~~~~~~~~~~~~~~~~~~~~~
159497178oke  ~~~~~~~~~~~~~~~~~~~~~~~~~~~~~~~~~~~~~~~~~~~~~~~~~~~~~~~~~~~~~~~~~~~~~~~~~~~~~~~~~~~~~~~~~~
159498304oke  ~~~~~~~~~~~~~~~~~~~~~~~~~~~~~~~~~~~~~~~~~~~~~~~~~~~~~~~~~~~~~~~~~~~~~~~~~~~~~~~~~~~~~~~~~~
159496326oke  ~~~~~~~~~~~~~~~~~~~~~~~~~~~~~~~~~~~~~~~~~~~~~~~~~~~~~~~~~~~~~~~~~~~~~~~~~~~~~~~~~~~~~~~~~~
159495404oke  ~~~~~~~~~~~~~~~~~~~~~~~~~~~~~~~~~~~~~~~~~~~~~~~~~~~~~~~~~~~~~~~~~~~~~~~~~~~~~~~~~~~~~~~~~~
159493141oke  ~~~~~~~~~~~~~~~~~~~~~~~~~~~~~~~~~~~~~~~~~~~~~~~~~~~~~~~~~~~~~~~~~~~~~~~~~~~~~~~~~~~~~~~~~~
159493570oke  ~~~~~~~~~~~~~~~~~~~~~~~~~~~~~~~~~~~~~~~~~~~~~~~~~~~~~~~~~~~~~~~~~~~~~~~~~~~~~~~~~~~~~~~~~~
159493499oke  ~~~~~~~~~~~~~~~~~~~~~~~~~~~~~~~~~~~~~~~~~~~~~~~~~~~~~~~~~~~~~~~~~~~~~~~~~~~~~~~~~~~~~~~~~~
159493217oke  ~~~~~~~~~~~~~~~~~~~~~~~~~~~~~~~~~~~~~~~~~~~~~~~~~~~~~~~~~~~~~~~~~~~~~~~~~~~~~~~~~~~~~~~~~~
159493216oke  ~~~~~~~~~~~~~~~~~~~~~~~~~~~~~~~~~~~~~~~~~~~~~~~~~~~~~~~~~~~~~~~~~~~~~~~~~~~~~~~~~~~~~~~~~~
159493234oke  ~~~~~~~~~~~~~~~~~~~~~~~~~~~~~~~~~~~~~~~~~~~~~~~~~~~~~~~~~~~~~~~~~~~~~~~~~~~~~~~~~~~~~~~~~~
159492908oke  ~~~~~~~~~~~~~~~~~~~~~~~~~~~~~~~~~~~~~~~~~~~~~~~~~~~~~~~~~~~~~~~~~~~~~~~~~~~~~~~~~~~~~~~~~~
159491934oke  ~~~~~~~~~~~~~~~~~~~~~~~~~~~~~~~~~~~~~~~~~~~~~~~~~~~~~~~~~~~~~~~~~~~~~~~~~~~~~~~~~~~~~~~~~~
159492538     ~~~~~~~~~~~~~~~~~~~~~~~~~~~~~~~~~~~~~~~~~~~~~~~~~~~~~~~~~~~~~~~~~~~~~~~~~~~~~~~~~~~~~~~~~~
159498161     ~~~~~~~~~~~~~~~~~~~~~~~~~~~~~~~~~~~~~~~~~~~~~~~~~~~~~~~~~~~~~~~~~~~~~~~~~~~~~~~~~~~~~~~~~~
159493124     ~~~~~~~~~~~~~~~~~~~~~~~~~~~~~~~~~~~~~~~~~~~~~~~~~~~~~~~~~~~~~~~~~~~~~~~~~~~~~~~~~~~~~~~~~~
159493661     ~~~~~~~~~~~~~~~~~~~~~~~~~~~~~~~~~~~~~~~~~~~~~~~~~~~~~~~~~~~~~~~~~~~~~~~~~~~~~~~~~~~~~~~~~~
159494727     ~~~~~~~~~~~~~~~~~~~~~~~~~~~~~~~~~~~~~~~~~~~~~~~~~~~~~~~~~~~~~~~~~~~~~~~~~~~~~~~~~~~~~~~~~~
159496944     ~~~~~~~~~~~~~~~~~~~~~~~~~~~~~~~~~~~~~~~~~~~~~~~~~~~~~~~~~~~~~~~~~~~~~~~~~~~~~~~~~~~~~~~~~~
159494492     ~~~~~~~~~~~~~~~~~~~~~~~~~~~~~~~~~~~~~~~~~~~~~~~~~~~~~~~~~~~~~~~~~~~~~~~~~~~~~~~~~~~~~~~~~~
159496140     ~~~~~~~~~~~~~~~~~~~~~~~~~~~~~~~~~~~~~~~~~~~~~~~~~~~~~~~~~~~~~~~~~~~~~~~~~~~~~~~~~~~~~~~~~~
159497160     ~~~~~~~~~~~~~~~~~~~~~~~~~~~~~~~~~~~~~~~~~~~~~~~~~~~~~~~~~~~~~~~~~~~~~~~~~~~~~~~~~~~~~~~~~~
aaa07c02.g1   ~~~~~~~~~~~~~~~~~~~~~~~~~~~~~~~~~~~~~~~~~~~~~~~~~~~~~~~~~~~~~~~~~~~~~~~~~~~~~~~~~~~~~~~~~~

                      730       740       750       760       770       780       790       800       810
              ....|....|....|....|....|....|....|....|....|....|....|....|....|....|....|....|....|....|
rsmitogen     agctttaaatattttattttgcatataactatagtaaaatttaattattattaaaaatttaatttagaaaaaaaaa~ttatttatttttt
rscox2
rstrna-his    agctt~~~~~~
rs16s         ~~~~~~~~~~~~~~~~~~~~~~~~~~~~~~~~~~~~~~~~~~~~~~~~~~~~~~~~~~~~~~~~~~~~~~~~~~~~~~~~~~~~~~~~~~
rsnad3        ~~~~~~~~~~~~~~~~~~~~~~~~~~~~~~~~~~~~~~~~~~~~~~~~~~~~~~~~~~~~~~~~~~~~~~~~~~~~~~~~~~~~~~~~~~
rstrna-leu    ~~~~~~~~~~~~~~~~~~~~~~~~~~~~~~~~~~~~~~~~~~~~~~~~~~~~~~~~~~~~~~~~~~~~~~~~~~~~~~~~~~~~~~~~~~
rscox3        ~~~~~~~~~~~~~~~~~~~~~~~~~~~~~~~~~~~~~~~~~~~~~~~~~~~~~~~~~~~~~~~~~~~~~~~~~~~~~~~~~~~~~~~~~~
rstrna-pro    ~~~~~~~~~~~~~~~~~~~~~~~~~~~~~~~~~~~~~~~~~~~~~~~~~~~~~~~~~~~~~~~~~~~~~~~~~~~~~~~~~~~~~~~~~~
rstrna-arg    ~~~~~~~~~~~~~~~~~~~~~~~~~~~~~~~~~~~~~~~~~~~~~~~~~~~~~~~~~~~~~~~~~~~~~~~~~~~~~~~~~~~~~~~~~~
rsnad6        ~~~~~~~~~~~~~~~~~~~~~~~~~~~~~~~~~~~~~~~~~~~~~~~~~~~~~~~~~~~~~~~~~~~~~~~~~~~~~~~~~~~~~~~~~~
rsnad4L       ~~~~~~~~~~~~~~~~~~~~~~~~~~~~~~~~~~~~~~~~~~~~~~~~~~~~~~~~~~~~~~~~~~~~~~~~~~~~~~~~~~~~~~~~~~
rstrna-val    ~~~~~~~~~~~~~~~~~~~~~~~~~~~~~~~~~~~~~~~~~~~~~~~~~~~~~~~~~~~~~~~~~~~~~~~~~~~~~~~~~~~~~~~~~~
rstrna-leu    ~~~~~~~~~~~~~~~~~~~~~~~~~~~~~~~~~~~~~~~~~~~~~~~~~~~~~~~~~~~~~~~~~~~~~~~~~~~~~~~~~~~~~~~~~~
159494961oke
159496557oke
159497093oke  AGCTTTAAATATTTTATTTTGCATATAACTATAGTAAAATTTAATTATTATTAAAAATTTAATTTAGAAAAAAAAA~TTATTTTTTTTTT
159496622oke  AGCTTTAAATATTTTATTTTGCATATAACTATAGTAAAATTTAATTATTATTAAAAATTTAATTTAGAAAAAAAAA~TTATTTATTTTTT
159498117oke  CCGGGTAAATATTTTATTTTGCATATAACTATAGTAAAATTTAATTATTATTAAAAATTTAATTTAAAAAAAAAAA~TTATTTATTTTTT
159494290oke  CCGGGTAAATATTTTATTTTGCATATAACTATAGTAAAATTTAATTATTATTAAAAATTTAATTTAAAAAAAAAAATTATTTATTTTTT
159495636oke  ~~~~~~~~~~~~~~~~~~~~~~~GGCCGGGATAGTAAAATTTAATTATTATTAAAAATTTAATTTAGAAAAAAAAAATTATTTATTTTTT
159497178oke  ~~~~~~~~~~~~~~~~~~~~~~~~~~~~~~~~~~~~~~~~~~~~~~~~~~~~~~~~~~~~~~~~~~~~~~~~~~~~~~~~~~~~~~~~~~
159498304oke  ~~~~~~~~~~~~~~~~~~~~~~~~~~~~~~~~~~~~~~~~~~~~~~~~~~~~~~~~~~~~~~~~~~~~~~~~~~~~~~~~~~~~~~~~~~
159496326oke  ~~~~~~~~~~~~~~~~~~~~~~~~~~~~~~~~~~~~~~~~~~~~~~~~~~~~~~~~~~~~~~~~~~~~~~~~~~~~~~~~~~~~~~~~~~
159495404oke  ~~~~~~~~~~~~~~~~~~~~~~~~~~~~~~~~~~~~~~~~~~~~~~~~~~~~~~~~~~~~~~~~~~~~~~~~~~~~~~~~~~~~~~~~~~
159493141oke  ~~~~~~~~~~~~~~~~~~~~~~~~~~~~~~~~~~~~~~~~~~~~~~~~~~~~~~~~~~~~~~~~~~~~~~~~~~~~~~~~~~~~~~~~~~
159493570oke  ~~~~~~~~~~~~~~~~~~~~~~~~~~~~~~~~~~~~~~~~~~~~~~~~~~~~~~~~~~~~~~~~~~~~~~~~~~~~~~~~~~~~~~~~~~
159493499oke  ~~~~~~~~~~~~~~~~~~~~~~~~~~~~~~~~~~~~~~~~~~~~~~~~~~~~~~~~~~~~~~~~~~~~~~~~~~~~~~~~~~~~~~~~~~
159493217oke  ~~~~~~~~~~~~~~~~~~~~~~~~~~~~~~~~~~~~~~~~~~~~~~~~~~~~~~~~~~~~~~~~~~~~~~~~~~~~~~~~~~~~~~~~~~
159493216oke  ~~~~~~~~~~~~~~~~~~~~~~~~~~~~~~~~~~~~~~~~~~~~~~~~~~~~~~~~~~~~~~~~~~~~~~~~~~~~~~~~~~~~~~~~~~
159493234oke  ~~~~~~~~~~~~~~~~~~~~~~~~~~~~~~~~~~~~~~~~~~~~~~~~~~~~~~~~~~~~~~~~~~~~~~~~~~~~~~~~~~~~~~~~~~
159492908oke  ~~~~~~~~~~~~~~~~~~~~~~~~~~~~~~~~~~~~~~~~~~~~~~~~~~~~~~~~~~~~~~~~~~~~~~~~~~~~~~~~~~~~~~~~~~
159491934oke  ~~~~~~~~~~~~~~~~~~~~~~~~~~~~~~~~~~~~~~~~~~~~~~~~~~~~~~~~~~~~~~~~~~~~~~~~~~~~~~~~~~~~~~~~~~
159492538     ~~~~~~~~~~~~~~~~~~~~~~~~~~~~~~~~~~~~~~~~~~~~~~~~~~~~~~~~~~~~~~~~~~~~~~~~~~~~~~~~~~~~~~~~~~
159498161     ~~~~~~~~~~~~~~~~~~~~~~~~~~~~~~~~~~~~~~~~~~~~~~~~~~~~~~~~~~~~~~~~~~~~~~~~~~~~~~~~~~~~~~~~~~
159493124     ~~~~~~~~~~~~~~~~~~~~~~~~~~~~~~~~~~~~~~~~~~~~~~~~~~~~~~~~~~~~~~~~~~~~~~~~~~~~~~~~~~~~~~~~~~
159493661     ~~~~~~~~~~~~~~~~~~~~~~~~~~~~~~~~~~~~~~~~~~~~~~~~~~~~~~~~~~~~~~~~~~~~~~~~~~~~~~~~~~~~~~~~~~
159494727     ~~~~~~~~~~~~~~~~~~~~~~~~~~~~~~~~~~~~~~~~~~~~~~~~~~~~~~~~~~~~~~~~~~~~~~~~~~~~~~~~~~~~~~~~~~
159496944     ~~~~~~~~~~~~~~~~~~~~~~~~~~~~~~~~~~~~~~~~~~~~~~~~~~~~~~~~~~~~~~~~~~~~~~~~~~~~~~~~~~~~~~~~~~
159494492     ~~~~~~~~~~~~~~~~~~~~~~~~~~~~~~~~~~~~~~~~~~~~~~~~~~~~~~~~~~~~~~~~~~~~~~~~~~~~~~~~~~~~~~~~~~
159496140     ~~~~~~~~~~~~~~~~~~~~~~~~~~~~~~~~~~~~~~~~~~~~~~~~~~~~~~~~~~~~~~~~~~~~~~~~~~~~~~~~~~~~~~~~~~
159497160     ~~~~~~~~~~~~~~~~~~~~~~~~~~~~~~~~~~~~~~~~~~~~~~~~~~~~~~~~~~~~~~~~~~~~~~~~~~~~~~~~~~~~~~~~~~
aaa07c02.g1   ~~~~~~~~~~~~~~~~~~~~~~~~~~~~~~~~~~~~~~~~~~~~~~~~~~~~~~~~~~~~~~~~~~~~~~~~~~~~~~~~~~~~~~~~~~

                      820       830       840       850       860       870       880       890       900
              ....|....|....|....|....|....|....|....|....|....|....|....|....|....|....|....|....|....|
rsmitogen     ttagtaacaataaaaaatatatttaaattttatattgatataatgattctaagaattaaagtttgtttataaaatattaaataatattag
rscox2
rstrna-his
rs16s         ~~~~~~~~aataaaaaatatatttaaattttatattgatataatgattctaagaattaaagtttgtttataaaatattaaataatattag
rsnad3        ~~~~~~~~~~~~~~~~~~~~~~~~~~~~~~~~~~~~~~~~~~~~~~~~~~~~~~~~~~~~~~~~~~~~~~~~~~~~~~~~~~~~~~~~~~
rstrna-leu    ~~~~~~~~~~~~~~~~~~~~~~~~~~~~~~~~~~~~~~~~~~~~~~~~~~~~~~~~~~~~~~~~~~~~~~~~~~~~~~~~~~~~~~~~~~
rscox3        ~~~~~~~~~~~~~~~~~~~~~~~~~~~~~~~~~~~~~~~~~~~~~~~~~~~~~~~~~~~~~~~~~~~~~~~~~~~~~~~~~~~~~~~~~~
rstrna-pro    ~~~~~~~~~~~~~~~~~~~~~~~~~~~~~~~~~~~~~~~~~~~~~~~~~~~~~~~~~~~~~~~~~~~~~~~~~~~~~~~~~~~~~~~~~~
rstrna-arg    ~~~~~~~~~~~~~~~~~~~~~~~~~~~~~~~~~~~~~~~~~~~~~~~~~~~~~~~~~~~~~~~~~~~~~~~~~~~~~~~~~~~~~~~~~~
rsnad6        ~~~~~~~~~~~~~~~~~~~~~~~~~~~~~~~~~~~~~~~~~~~~~~~~~~~~~~~~~~~~~~~~~~~~~~~~~~~~~~~~~~~~~~~~~~
rsnad4L       ~~~~~~~~~~~~~~~~~~~~~~~~~~~~~~~~~~~~~~~~~~~~~~~~~~~~~~~~~~~~~~~~~~~~~~~~~~~~~~~~~~~~~~~~~~
rstrna-val    ~~~~~~~~~~~~~~~~~~~~~~~~~~~~~~~~~~~~~~~~~~~~~~~~~~~~~~~~~~~~~~~~~~~~~~~~~~~~~~~~~~~~~~~~~~
rstrna-leu    ~~~~~~~~~~~~~~~~~~~~~~~~~~~~~~~~~~~~~~~~~~~~~~~~~~~~~~~~~~~~~~~~~~~~~~~~~~~~~~~~~~~~~~~~~~
159494961oke
159496557oke
159497093oke  TTAGTACCAATAAAAAAAATATTTAAATTTTATATTGATATAAGGATTTTAAGAATTAAAGTTTGTTTATAAAATATTAAATAATATTAG
159496622oke  TTAGTAACAATAAAAAATATATTTAAATTTTATATTGATATAATGATTCTAAGAATTAAAGTTTGTTTATAAAATATTAAATAATATTAG
159498117oke  TTAGTAACAATAAAAAATATATTTAAATTTTATATTGATATAATGATTCTAAGAATTAAAGTTTGTTTATAAAATATTAAATAATATTAG
159494290oke  TTAGTAACAATAAAAAATATATTTAAATTTTATATTGATATAATGATTCTAAGAATTAAAGTTTGTTTATAAAATATTAAATAATATTAG
159495636oke  TTAGTAACAATAAAAAATATATTTAAATTT~ATATTGATATAATGATTCTAAGAATTAAAGTTTGTTTATAAAATATTAAATAATATTAG
159497178oke  ~~~~~~~~~~~~~~~~~~~~~~~~~~~~~~~~~~~~~~~~~~~~~~~~~~~~~~~~~~~~~~~~~~~~~~~~~~~~GGCCGGGATATTAG
159498304oke  ~~~~~~~~~~~~~~~~~~~~~~~~~~~~~~~~~~~~~~~~~~~~~~~~~~~~~~~~~~~~~~~~~~~~~~~~~~~~~~~~~~~~~~~~~~
159496326oke  ~~~~~~~~~~~~~~~~~~~~~~~~~~~~~~~~~~~~~~~~~~~~~~~~~~~~~~~~~~~~~~~~~~~~~~~~~~~~~~~~~~~~~~~~~~
159495404oke  ~~~~~~~~~~~~~~~~~~~~~~~~~~~~~~~~~~~~~~~~~~~~~~~~~~~~~~~~~~~~~~~~~~~~~~~~~~~~~~~~~~~~~~~~~~
159493141oke  ~~~~~~~~~~~~~~~~~~~~~~~~~~~~~~~~~~~~~~~~~~~~~~~~~~~~~~~~~~~~~~~~~~~~~~~~~~~~~~~~~~~~~~~~~~
159493570oke  ~~~~~~~~~~~~~~~~~~~~~~~~~~~~~~~~~~~~~~~~~~~~~~~~~~~~~~~~~~~~~~~~~~~~~~~~~~~~~~~~~~~~~~~~~~
159493499oke  ~~~~~~~~~~~~~~~~~~~~~~~~~~~~~~~~~~~~~~~~~~~~~~~~~~~~~~~~~~~~~~~~~~~~~~~~~~~~~~~~~~~~~~~~~~
159493217oke  ~~~~~~~~~~~~~~~~~~~~~~~~~~~~~~~~~~~~~~~~~~~~~~~~~~~~~~~~~~~~~~~~~~~~~~~~~~~~~~~~~~~~~~~~~~
159493216oke  ~~~~~~~~~~~~~~~~~~~~~~~~~~~~~~~~~~~~~~~~~~~~~~~~~~~~~~~~~~~~~~~~~~~~~~~~~~~~~~~~~~~~~~~~~~
159493234oke  ~~~~~~~~~~~~~~~~~~~~~~~~~~~~~~~~~~~~~~~~~~~~~~~~~~~~~~~~~~~~~~~~~~~~~~~~~~~~~~~~~~~~~~~~~~
159492908oke  ~~~~~~~~~~~~~~~~~~~~~~~~~~~~~~~~~~~~~~~~~~~~~~~~~~~~~~~~~~~~~~~~~~~~~~~~~~~~~~~~~~~~~~~~~~
159491934oke  ~~~~~~~~~~~~~~~~~~~~~~~~~~~~~~~~~~~~~~~~~~~~~~~~~~~~~~~~~~~~~~~~~~~~~~~~~~~~~~~~~~~~~~~~~~
159492538     ~~~~~~~~~~~~~~~~~~~~~~~~~~~~~~~~~~~~~~~~~~~~~~~~~~~~~~~~~~~~~~~~~~~~~~~~~~~~~~~~~~~~~~~~~~
159498161     ~~~~~~~~~~~~~~~~~~~~~~~~~~~~~~~~~~~~~~~~~~~~~~~~~~~~~~~~~~~~~~~~~~~~~~~~~~~~~~~~~~~~~~~~~~
159493124     ~~~~~~~~~~~~~~~~~~~~~~~~~~~~~~~~~~~~~~~~~~~~~~~~~~~~~~~~~~~~~~~~~~~~~~~~~~~~~~~~~~~~~~~~~~
159493661     ~~~~~~~~~~~~~~~~~~~~~~~~~~~~~~~~~~~~~~~~~~~~~~~~~~~~~~~~~~~~~~~~~~~~~~~~~~~~~~~~~~~~~~~~~~
159494727     ~~~~~~~~~~~~~~~~~~~~~~~~~~~~~~~~~~~~~~~~~~~~~~~~~~~~~~~~~~~~~~~~~~~~~~~~~~~~~~~~~~~~~~~~~~
159496944     ~~~~~~~~~~~~~~~~~~~~~~~~~~~~~~~~~~~~~~~~~~~~~~~~~~~~~~~~~~~~~~~~~~~~~~~~~~~~~~~~~~~~~~~~~~
159494492     ~~~~~~~~~~~~~~~~~~~~~~~~~~~~~~~~~~~~~~~~~~~~~~~~~~~~~~~~~~~~~~~~~~~~~~~~~~~~~~~~~~~~~~~~~~
159496140     ~~~~~~~~~~~~~~~~~~~~~~~~~~~~~~~~~~~~~~~~~~~~~~~~~~~~~~~~~~~~~~~~~~~~~~~~~~~~~~~~~~~~~~~~~~
159497160     ~~~~~~~~~~~~~~~~~~~~~~~~~~~~~~~~~~~~~~~~~~~~~~~~~~~~~~~~~~~~~~~~~~~~~~~~~~~~~~~~~~~~~~~~~~
aaa07c02.g1   ~~~~~~~~~~~~~~~~~~~~~~~~~~~~~~~~~~~~~~~~~~~~~~~~~~~~~~~~~~~~~~~~~~~~~~~~~~~~~~~~~~~~~~~~~~

                      910       920       930       940       950       960       970       980       990
              ....|....|....|....|....|....|....|....|....|....|....|....|....|....|....|....|....|....|
rsmitogen     ttattttatttttttaaaatttattaaaaattaataaatttaaaatcttttagattattatataattatattttataatttatttttaaa
rscox2
rstrna-his
rs16s         ttattttatttttttaaaatttattaaaaattaataaatttaaaatcttttagattattatataattatattttataatttatttttaaa
rsnad3        ~~~~~~~~~~~~~~~~~~~~~~~~~~~~~~~~~~~~~~~~~~~~~~~~~~~~~~~~~~~~~~~~~~~~~~~~~~~~~~~~~~~~~~~~~~
rstrna-leu    ~~~~~~~~~~~~~~~~~~~~~~~~~~~~~~~~~~~~~~~~~~~~~~~~~~~~~~~~~~~~~~~~~~~~~~~~~~~~~~~~~~~~~~~~~~
rscox3        ~~~~~~~~~~~~~~~~~~~~~~~~~~~~~~~~~~~~~~~~~~~~~~~~~~~~~~~~~~~~~~~~~~~~~~~~~~~~~~~~~~~~~~~~~~
rstrna-pro    ~~~~~~~~~~~~~~~~~~~~~~~~~~~~~~~~~~~~~~~~~~~~~~~~~~~~~~~~~~~~~~~~~~~~~~~~~~~~~~~~~~~~~~~~~~
```

```
rstrna-arg    ~~~~~~~~~~~~~~~~~~~~~~~~~~~~~~~~~~~~~~~~~~~~~~~~~~~~~~~~~~~~~~~~~~~~~~~~~~~~~~~~~~~~~~~~~~
rsnad6        ~~~~~~~~~~~~~~~~~~~~~~~~~~~~~~~~~~~~~~~~~~~~~~~~~~~~~~~~~~~~~~~~~~~~~~~~~~~~~~~~~~~~~~~~~~
rsnad4L       ~~~~~~~~~~~~~~~~~~~~~~~~~~~~~~~~~~~~~~~~~~~~~~~~~~~~~~~~~~~~~~~~~~~~~~~~~~~~~~~~~~~~~~~~~~
rstrna-val    ~~~~~~~~~~~~~~~~~~~~~~~~~~~~~~~~~~~~~~~~~~~~~~~~~~~~~~~~~~~~~~~~~~~~~~~~~~~~~~~~~~~~~~~~~~
rstrna-leu    ~~~~~~~~~~~~~~~~~~~~~~~~~~~~~~~~~~~~~~~~~~~~~~~~~~~~~~~~~~~~~~~~~~~~~~~~~~~~~~~~~~~~~~~~~~
159494961oke
159496557oke
159497093oke  TTATTTTATTTTTT-AAAATTTATTAAAAATTAATAAATTTAAAATCTTTTAGATTATTATATAATTATATTTTATAATTTATTTTTAAA
159496622oke  TTATTTTATTTTTTTAAAATTTATTAAAAATTAATAAATTTAAAATCTTTTAGATTATTATATAATTATATTTTATAATTTATTTTTAAA
159498117oke  TTATTTTATTTTTTTAAAATTTATTAAAAATTAATAAATTTAAAATCTTTTAGATTATTATATAATTATATTTTATAATTTATTTTTAAA
159494290oke  TTATTTTATTTTTTTAAAATTTATTAAAAATTAATAAATTTAAAATCTTTTAGATTATTATATAATTATATTTTATAATTTATTTTTAAA
159495636oke  TTATTTTATTTTTTTAAAATTTATTAAAAATTAATAAATTTAAAATCTTTTAGATTATTATATAATTATATTTTATAATTTATTTTTAAA
159497178oke  TTATTTTATTTTTTTAAAATTTATTAAAAATTAATAAATTTAAAATCTTTTAGATTATTATATAATTATATTTTATAATTTATTTTTAAA
159498304oke  ~~~~~~~~~~~~~~~~~~~~~~~~~~~~~~~~~~~~~~~~~~~~~~~~~~~~~~~~~~~~~~~~~~~~~~~~~~~~~~~~~~~~~~~~~~
159496326oke  ~~~~~~~~~~~~~~~~~~~~~~~~~~~~~~~~~~~~~~~~~~~~~~~~~~~~~~~~~~~~~~~~~~~~~~~~~~~~~~~~~~~~~~~~~~
159495404oke  ~~~~~~~~~~~~~~~~~~~~~~~~~~~~~~~~~~~~~~~~~~~~~~~~~~~~~~~~~~~~~~~~~~~~~~~~~~~~~~~~~~~~~~~~~~
159493141oke  ~~~~~~~~~~~~~~~~~~~~~~~~~~~~~~~~~~~~~~~~~~~~~~~~~~~~~~~~~~~~~~~~~~~~~~~~~~~~~~~~~~~~~~~~~~
159493570oke  ~~~~~~~~~~~~~~~~~~~~~~~~~~~~~~~~~~~~~~~~~~~~~~~~~~~~~~~~~~~~~~~~~~~~~~~~~~~~~~~~~~~~~~~~~~
159493499oke  ~~~~~~~~~~~~~~~~~~~~~~~~~~~~~~~~~~~~~~~~~~~~~~~~~~~~~~~~~~~~~~~~~~~~~~~~~~~~~~~~~~~~~~~~~~
159493217oke  ~~~~~~~~~~~~~~~~~~~~~~~~~~~~~~~~~~~~~~~~~~~~~~~~~~~~~~~~~~~~~~~~~~~~~~~~~~~~~~~~~~~~~~~~~~
159493216oke  ~~~~~~~~~~~~~~~~~~~~~~~~~~~~~~~~~~~~~~~~~~~~~~~~~~~~~~~~~~~~~~~~~~~~~~~~~~~~~~~~~~~~~~~~~~
159493234oke  ~~~~~~~~~~~~~~~~~~~~~~~~~~~~~~~~~~~~~~~~~~~~~~~~~~~~~~~~~~~~~~~~~~~~~~~~~~~~~~~~~~~~~~~~~~
159492908oke  ~~~~~~~~~~~~~~~~~~~~~~~~~~~~~~~~~~~~~~~~~~~~~~~~~~~~~~~~~~~~~~~~~~~~~~~~~~~~~~~~~~~~~~~~~~
159491934oke  ~~~~~~~~~~~~~~~~~~~~~~~~~~~~~~~~~~~~~~~~~~~~~~~~~~~~~~~~~~~~~~~~~~~~~~~~~~~~~~~~~~~~~~~~~~
159492538     ~~~~~~~~~~~~~~~~~~~~~~~~~~~~~~~~~~~~~~~~~~~~~~~~~~~~~~~~~~~~~~~~~~~~~~~~~~~~~~~~~~~~~~~~~~
159498161     ~~~~~~~~~~~~~~~~~~~~~~~~~~~~~~~~~~~~~~~~~~~~~~~~~~~~~~~~~~~~~~~~~~~~~~~~~~~~~~~~~~~~~~~~~~
159493124     ~~~~~~~~~~~~~~~~~~~~~~~~~~~~~~~~~~~~~~~~~~~~~~~~~~~~~~~~~~~~~~~~~~~~~~~~~~~~~~~~~~~~~~~~~~
159493661     ~~~~~~~~~~~~~~~~~~~~~~~~~~~~~~~~~~~~~~~~~~~~~~~~~~~~~~~~~~~~~~~~~~~~~~~~~~~~~~~~~~~~~~~~~~
159494727     ~~~~~~~~~~~~~~~~~~~~~~~~~~~~~~~~~~~~~~~~~~~~~~~~~~~~~~~~~~~~~~~~~~~~~~~~~~~~~~~~~~~~~~~~~~
159496944     ~~~~~~~~~~~~~~~~~~~~~~~~~~~~~~~~~~~~~~~~~~~~~~~~~~~~~~~~~~~~~~~~~~~~~~~~~~~~~~~~~~~~~~~~~~
159494492     ~~~~~~~~~~~~~~~~~~~~~~~~~~~~~~~~~~~~~~~~~~~~~~~~~~~~~~~~~~~~~~~~~~~~~~~~~~~~~~~~~~~~~~~~~~
159496140     ~~~~~~~~~~~~~~~~~~~~~~~~~~~~~~~~~~~~~~~~~~~~~~~~~~~~~~~~~~~~~~~~~~~~~~~~~~~~~~~~~~~~~~~~~~
159497160     ~~~~~~~~~~~~~~~~~~~~~~~~~~~~~~~~~~~~~~~~~~~~~~~~~~~~~~~~~~~~~~~~~~~~~~~~~~~~~~~~~~~~~~~~~~
aaa07c02.g1   ~~~~~~~~~~~~~~~~~~~~~~~~~~~~~~~~~~~~~~~~~~~~~~~~~~~~~~~~~~~~~~~~~~~~~~~~~~~~~~~~~~~~~~~~~~

                   1000      1010      1020      1030      1040      1050      1060      1070      1080
              ....|....|....|....|....|....|....|....|....|....|....|....|....|....|....|....|....|....|
rsmitogen     atttatttttttaaaataagtaaaatttgagatattattttgattttatgatgttttttt~aaatgtttttttctgcccaatgttagtat
rscox2
rstrna-his
rs16s         atttatttttttaaaataagtaaaatttgagatattattttgattttatgatgttttttt~aaatgtttttttctgcccaatgttagtat
rsnad3        ~~~~~~~~~~~~~~~~~~~~~~~~~~~~~~~~~~~~~~~~~~~~~~~~~~~~~~~~~~~~~~~~~~~~~~~~~~~~~~~~~~~~~~~~~~
rstrna-leu    ~~~~~~~~~~~~~~~~~~~~~~~~~~~~~~~~~~~~~~~~~~~~~~~~~~~~~~~~~~~~~~~~~~~~~~~~~~~~~~~~~~~~~~~~~~
rscox3        ~~~~~~~~~~~~~~~~~~~~~~~~~~~~~~~~~~~~~~~~~~~~~~~~~~~~~~~~~~~~~~~~~~~~~~~~~~~~~~~~~~~~~~~~~~
rstrna-pro    ~~~~~~~~~~~~~~~~~~~~~~~~~~~~~~~~~~~~~~~~~~~~~~~~~~~~~~~~~~~~~~~~~~~~~~~~~~~~~~~~~~~~~~~~~~
rstrna-arg    ~~~~~~~~~~~~~~~~~~~~~~~~~~~~~~~~~~~~~~~~~~~~~~~~~~~~~~~~~~~~~~~~~~~~~~~~~~~~~~~~~~~~~~~~~~
rsnad6        ~~~~~~~~~~~~~~~~~~~~~~~~~~~~~~~~~~~~~~~~~~~~~~~~~~~~~~~~~~~~~~~~~~~~~~~~~~~~~~~~~~~~~~~~~~
rsnad4L       ~~~~~~~~~~~~~~~~~~~~~~~~~~~~~~~~~~~~~~~~~~~~~~~~~~~~~~~~~~~~~~~~~~~~~~~~~~~~~~~~~~~~~~~~~~
rstrna-val    ~~~~~~~~~~~~~~~~~~~~~~~~~~~~~~~~~~~~~~~~~~~~~~~~~~~~~~~~~~~~~~~~~~~~~~~~~~~~~~~~~~~~~~~~~~
rstrna-leu    ~~~~~~~~~~~~~~~~~~~~~~~~~~~~~~~~~~~~~~~~~~~~~~~~~~~~~~~~~~~~~~~~~~~~~~~~~~~~~~~~~~~~~~~~~~
159494961oke
159496557oke
159497093oke  ATTTATTTTTTTAAAATAAGTAAAATTTGAGATATTATTTTGATTTTATGATGTTTTTTT~AAATGTTTTTTTCTGCCCAATGTTAGTAC
159496622oke  ATTTATTTTTTTAAAATAAGTAAAATTTGAGATATTATTTTGATTTTATGATGTTTTTTT~AAATGTTTTTTTCTGCCCAATGTTAGTAC
159498117oke  ATTTATTTTTTTAAAATAAGTAAAATTTGAGATATTATTTTGATTTTATGATGTTTTTTT~AAATGTTTTTTTCTGCCCAATGTTAGTAT
159494290oke  ATTTATTTTTTTAAAATAAGTAAAATTTGAGATATTATTTTGATTTTATGATGTTTTTTT-AAATGTTTTTTTCTGCCCAATGTTAGTAT
159495636oke  ATTTATTTTTTTAAAATAAGTAAAATTTGAGATATTATTTTGATTTTATGATGTTTTTTT-AAATGTTTTTTTCTGCCCAATGTTAGTAT
159497178oke  ATTTATTTTTTTAAAATAAGTAAAATTTGAGATATTATTTTGATTTTATGATGTTTTTTTTAAATGTTTTTTTCTGCCCAATGTTAGTAT
159498304oke  ~~~~~~~~~~~~~~~~~~~~~~~~~~~~~~~~~~~~~~~~~~~~~~~~~~~~~~~~~~~~~~~~~~~~~~~~~~~~~~~~~~~~~~~GGC
159496326oke  ~~~~~~~~~~~~~~~~~~~~~~~~~~~~~~~~~~~~~~~~~~~~~~~~~~~~~~~~~~~~~~~~~~~~~~~~~~~~~~~~~~~~~~~~~~
159495404oke  ~~~~~~~~~~~~~~~~~~~~~~~~~~~~~~~~~~~~~~~~~~~~~~~~~~~~~~~~~~~~~~~~~~~~~~~~~~~~~~~~~~~~~~~~~~
159493141oke  ~~~~~~~~~~~~~~~~~~~~~~~~~~~~~~~~~~~~~~~~~~~~~~~~~~~~~~~~~~~~~~~~~~~~~~~~~~~~~~~~~~~~~~~~~~
159493570oke  ~~~~~~~~~~~~~~~~~~~~~~~~~~~~~~~~~~~~~~~~~~~~~~~~~~~~~~~~~~~~~~~~~~~~~~~~~~~~~~~~~~~~~~~~~~
159493499oke  ~~~~~~~~~~~~~~~~~~~~~~~~~~~~~~~~~~~~~~~~~~~~~~~~~~~~~~~~~~~~~~~~~~~~~~~~~~~~~~~~~~~~~~~~~~
159493217oke  ~~~~~~~~~~~~~~~~~~~~~~~~~~~~~~~~~~~~~~~~~~~~~~~~~~~~~~~~~~~~~~~~~~~~~~~~~~~~~~~~~~~~~~~~~~
159493216oke  ~~~~~~~~~~~~~~~~~~~~~~~~~~~~~~~~~~~~~~~~~~~~~~~~~~~~~~~~~~~~~~~~~~~~~~~~~~~~~~~~~~~~~~~~~~
159493234oke  ~~~~~~~~~~~~~~~~~~~~~~~~~~~~~~~~~~~~~~~~~~~~~~~~~~~~~~~~~~~~~~~~~~~~~~~~~~~~~~~~~~~~~~~~~~
159492908oke  ~~~~~~~~~~~~~~~~~~~~~~~~~~~~~~~~~~~~~~~~~~~~~~~~~~~~~~~~~~~~~~~~~~~~~~~~~~~~~~~~~~~~~~~~~~
159491934oke  ~~~~~~~~~~~~~~~~~~~~~~~~~~~~~~~~~~~~~~~~~~~~~~~~~~~~~~~~~~~~~~~~~~~~~~~~~~~~~~~~~~~~~~~~~~
159492538     ~~~~~~~~~~~~~~~~~~~~~~~~~~~~~~~~~~~~~~~~~~~~~~~~~~~~~~~~~~~~~~~~~~~~~~~~~~~~~~~~~~~~~~~~~~
159498161     ~~~~~~~~~~~~~~~~~~~~~~~~~~~~~~~~~~~~~~~~~~~~~~~~~~~~~~~~~~~~~~~~~~~~~~~~~~~~~~~~~~~~~~~~~~
159493124     ~~~~~~~~~~~~~~~~~~~~~~~~~~~~~~~~~~~~~~~~~~~~~~~~~~~~~~~~~~~~~~~~~~~~~~~~~~~~~~~~~~~~~~~~~~
159493661     ~~~~~~~~~~~~~~~~~~~~~~~~~~~~~~~~~~~~~~~~~~~~~~~~~~~~~~~~~~~~~~~~~~~~~~~~~~~~~~~~~~~~~~~~~~
159494727     ~~~~~~~~~~~~~~~~~~~~~~~~~~~~~~~~~~~~~~~~~~~~~~~~~~~~~~~~~~~~~~~~~~~~~~~~~~~~~~~~~~~~~~~~~~
159496944     ~~~~~~~~~~~~~~~~~~~~~~~~~~~~~~~~~~~~~~~~~~~~~~~~~~~~~~~~~~~~~~~~~~~~~~~~~~~~~~~~~~~~~~~~~~
159494492     ~~~~~~~~~~~~~~~~~~~~~~~~~~~~~~~~~~~~~~~~~~~~~~~~~~~~~~~~~~~~~~~~~~~~~~~~~~~~~~~~~~~~~~~~~~
159496140     ~~~~~~~~~~~~~~~~~~~~~~~~~~~~~~~~~~~~~~~~~~~~~~~~~~~~~~~~~~~~~~~~~~~~~~~~~~~~~~~~~~~~~~~~~~
159497160     ~~~~~~~~~~~~~~~~~~~~~~~~~~~~~~~~~~~~~~~~~~~~~~~~~~~~~~~~~~~~~~~~~~~~~~~~~~~~~~~~~~~~~~~~~~
aaa07c02.g1   ~~~~~~~~~~~~~~~~~~~~~~~~~~~~~~~~~~~~~~~~~~~~~~~~~~~~~~~~~~~~~~~~~~~~~~~~~~~~~~~~~~~~~~~~~~

                   1090      1100      1110      1120      1130      1140      1150      1160      1170
              ....|....|....|....|....|....|....|....|....|....|....|....|....|....|....|....|....|....|
rsmitogen     taatggcgcttttagcgtgaggagcttatagtagcacatgttacttgttttttt~attaaatccggggatgaatgaaagattcaaatttg
rscox2
rstrna-his
rs16s         taatggcgcttttagcgtgaggagcttatagtagcacatgttacttgttttttt~attaaatccggggatgaatgaaagattcaaatttg
rsnad3        ~~~~~~~~~~~~~~~~~~~~~~~~~~~~~~~~~~~~~~~~~~~~~~~~~~~~~~~~~~~~~~~~~~~~~~~~~~~~~~~~~~~~~~~~~~
rstrna-leu    ~~~~~~~~~~~~~~~~~~~~~~~~~~~~~~~~~~~~~~~~~~~~~~~~~~~~~~~~~~~~~~~~~~~~~~~~~~~~~~~~~~~~~~~~~~
rscox3        ~~~~~~~~~~~~~~~~~~~~~~~~~~~~~~~~~~~~~~~~~~~~~~~~~~~~~~~~~~~~~~~~~~~~~~~~~~~~~~~~~~~~~~~~~~
rstrna-pro    ~~~~~~~~~~~~~~~~~~~~~~~~~~~~~~~~~~~~~~~~~~~~~~~~~~~~~~~~~~~~~~~~~~~~~~~~~~~~~~~~~~~~~~~~~~
rstrna-arg    ~~~~~~~~~~~~~~~~~~~~~~~~~~~~~~~~~~~~~~~~~~~~~~~~~~~~~~~~~~~~~~~~~~~~~~~~~~~~~~~~~~~~~~~~~~
rsnad6        ~~~~~~~~~~~~~~~~~~~~~~~~~~~~~~~~~~~~~~~~~~~~~~~~~~~~~~~~~~~~~~~~~~~~~~~~~~~~~~~~~~~~~~~~~~
rsnad4L       ~~~~~~~~~~~~~~~~~~~~~~~~~~~~~~~~~~~~~~~~~~~~~~~~~~~~~~~~~~~~~~~~~~~~~~~~~~~~~~~~~~~~~~~~~~
rstrna-val    ~~~~~~~~~~~~~~~~~~~~~~~~~~~~~~~~~~~~~~~~~~~~~~~~~~~~~~~~~~~~~~~~~~~~~~~~~~~~~~~~~~~~~~~~~~
rstrna-leu    ~~~~~~~~~~~~~~~~~~~~~~~~~~~~~~~~~~~~~~~~~~~~~~~~~~~~~~~~~~~~~~~~~~~~~~~~~~~~~~~~~~~~~~~~~~
159494961oke
159496557oke
159497093oke  CCGGCC~~~
159496622oke  CCGGCC~~~
159498117oke  TAATGGCGCTTTTAGCGTGAGGAGCTTATAGTAGCACATGTTACTTGTTTTTTT~ATTAAATCCGGGGATGAATGAAAGATTCAAATTTG
159494290oke  TAATGGCGCTTTTAGCGTGAGGAGCTTATAGTAGCACATGTTACTTGTTTTTTTTATTAAATCCGGGGATGAATGAAAGATTCAAATTTG
159495636oke  TAATGGCGCTTTTAGCGTGAGGAGCTTATAGTAGCACATGTTACTTGTTTTTTT~ATTAAATCCGGGGATAGTAAAATTTAATTATTATT
159497178oke  TAATGGCGCTTTTAGCGTGAGGAGCTTATAGTAGCACATGTTACTTGTTTTTTT~ATTAAATCCGGGGATGAATGAAAGATTCAAATTTG
159498304oke  CGGGGGCGCTTTTAGCGTGAGGAGCTTATAGTAGCACATGTTACTTGTTTTTTT~ATTAAATCCGGGGATGAATGAAAGATTCAAATTTG
159496326oke  ~~~~~~~~~~~~GGCCGGGAGGAGCTTATAGTAGCACATGTTACTTGTTTTTTT~ATTAAATCCGGGGATGAATGAAAGATTCAAATTTG
159495404oke  ~~~~~~~~~GGCCGGGGTGAGGAGCTTATAGTAGCACATGTTACTTGCTTTTTT~ATTAAATCCGGGGATGAATGAAAGATTCAAATTTG
159493141oke  ~~~~~~~~~~~~~~~~~~~~~~~~~~~~~~~~~~~~~~~~~~~~~~~~~~~~~~~~~~~~~~~~~~~~~~~~~~~~~~~~~~~~~~~~~~
159493570oke  ~~~~~~~~~~~~~~~~~~~~~~~~~~~~~~~~~~~~~~~~~~~~~~~~~~~~~~~~~~~~~~~~~~~~~~~~~~~~~~~~~~~~~~~~~~
159493499oke  ~~~~~~~~~~~~~~~~~~~~~~~~~~~~~~~~~~~~~~~~~~~~~~~~~~~~~~~~~~~~~~~~~~~~~~~~~~~~~~~~~~~~~~~~~~
159493217oke  ~~~~~~~~~~~~~~~~~~~~~~~~~~~~~~~~~~~~~~~~~~~~~~~~~~~~~~~~~~~~~~~~~~~~~~~~~~~~~~~~~~~~~~~~~~
159493216oke  ~~~~~~~~~~~~~~~~~~~~~~~~~~~~~~~~~~~~~~~~~~~~~~~~~~~~~~~~~~~~~~~~~~~~~~~~~~~~~~~~~~~~~~~~~~
159493234oke  ~~~~~~~~~~~~~~~~~~~~~~~~~~~~~~~~~~~~~~~~~~~~~~~~~~~~~~~~~~~~~~~~~~~~~~~~~~~~~~~~~~~~~~~~~~
159492908oke  ~~~~~~~~~~~~~~~~~~~~~~~~~~~~~~~~~~~~~~~~~~~~~~~~~~~~~~~~~~~~~~~~~~~~~~~~~~~~~~~~~~~~~~~~~~
159491934oke  ~~~~~~~~~~~~~~~~~~~~~~~~~~~~~~~~~~~~~~~~~~~~~~~~~~~~~~~~~~~~~~~~~~~~~~~~~~~~~~~~~~~~~~~~~~
159492538     ~~~~~~~~~~~~~~~~~~~~~~~~~~~~~~~~~~~~~~~~~~~~~~~~~~~~~~~~~~~~~~~~~~~~~~~~~~~~~~~~~~~~~~~~~~
159498161     ~~~~~~~~~~~~~~~~~~~~~~~~~~~~~~~~~~~~~~~~~~~~~~~~~~~~~~~~~~~~~~~~~~~~~~~~~~~~~~~~~~~~~~~~~~
159493124     ~~~~~~~~~~~~~~~~~~~~~~~~~~~~~~~~~~~~~~~~~~~~~~~~~~~~~~~~~~~~~~~~~~~~~~~~~~~~~~~~~~~~~~~~~~
159493661     ~~~~~~~~~~~~~~~~~~~~~~~~~~~~~~~~~~~~~~~~~~~~~~~~~~~~~~~~~~~~~~~~~~~~~~~~~~~~~~~~~~~~~~~~~~
159494727     ~~~~~~~~~~~~~~~~~~~~~~~~~~~~~~~~~~~~~~~~~~~~~~~~~~~~~~~~~~~~~~~~~~~~~~~~~~~~~~~~~~~~~~~~~~
159496944     ~~~~~~~~~~~~~~~~~~~~~~~~~~~~~~~~~~~~~~~~~~~~~~~~~~~~~~~~~~~~~~~~~~~~~~~~~~~~~~~~~~~~~~~~~~
159494492     ~~~~~~~~~~~~~~~~~~~~~~~~~~~~~~~~~~~~~~~~~~~~~~~~~~~~~~~~~~~~~~~~~~~~~~~~~~~~~~~~~~~~~~~~~~
159496140     ~~~~~~~~~~~~~~~~~~~~~~~~~~~~~~~~~~~~~~~~~~~~~~~~~~~~~~~~~~~~~~~~~~~~~~~~~~~~~~~~~~~~~~~~~~
159497160     ~~~~~~~~~~~~~~~~~~~~~~~~~~~~~~~~~~~~~~~~~~~~~~~~~~~~~~~~~~~~~~~~~~~~~~~~~~~~~~~~~~~~~~~~~~
aaa07c02.g1   ~~~~~~~~~~~~~~~~~~~~~~~~~~~~~~~~~~~~~~~~~~~~~~~~~~~~~~~~~~~~~~~~~~~~~~~~~~~~~~~~~~~~~~~~~~

                   1180      1190      1200      1210      1220      1230      1240      1250      1260
              ....|....|....|....|....|....|....|....|....|....|....|....|....|....|....|....|....|....|
rsmitogen     atgtaaattatatttttagaatttaataaaaatttaataattaatttttttttt~~aaaactaagaaaagtctttggaaatttatttttt
rscox2
rstrna-his
rs16s         atgtaaattatatttttagaatttaataaaaatttaataattaatttttttttt~~aaaactaagaaaagtctttggaaatttatttttt
rsnad3        ~~~~~~~~~~~~~~~~~~~~~~~~~~~~~~~~~~~~~~~~~~~~~~~~~~~~~~~~~~~~~~~~~~~~~~~~~~~~~~~~~~~~~~~~~~
rstrna-leu    ~~~~~~~~~~~~~~~~~~~~~~~~~~~~~~~~~~~~~~~~~~~~~~~~~~~~~~~~~~~~~~~~~~~~~~~~~~~~~~~~~~~~~~~~~~
rscox3        ~~~~~~~~~~~~~~~~~~~~~~~~~~~~~~~~~~~~~~~~~~~~~~~~~~~~~~~~~~~~~~~~~~~~~~~~~~~~~~~~~~~~~~~~~~
rstrna-pro    ~~~~~~~~~~~~~~~~~~~~~~~~~~~~~~~~~~~~~~~~~~~~~~~~~~~~~~~~~~~~~~~~~~~~~~~~~~~~~~~~~~~~~~~~~~
rstrna-arg    ~~~~~~~~~~~~~~~~~~~~~~~~~~~~~~~~~~~~~~~~~~~~~~~~~~~~~~~~~~~~~~~~~~~~~~~~~~~~~~~~~~~~~~~~~~
rsnad6        ~~~~~~~~~~~~~~~~~~~~~~~~~~~~~~~~~~~~~~~~~~~~~~~~~~~~~~~~~~~~~~~~~~~~~~~~~~~~~~~~~~~~~~~~~~
rsnad4L       ~~~~~~~~~~~~~~~~~~~~~~~~~~~~~~~~~~~~~~~~~~~~~~~~~~~~~~~~~~~~~~~~~~~~~~~~~~~~~~~~~~~~~~~~~~
rstrna-val    ~~~~~~~~~~~~~~~~~~~~~~~~~~~~~~~~~~~~~~~~~~~~~~~~~~~~~~~~~~~~~~~~~~~~~~~~~~~~~~~~~~~~~~~~~~
rstrna-leu    ~~~~~~~~~~~~~~~~~~~~~~~~~~~~~~~~~~~~~~~~~~~~~~~~~~~~~~~~~~~~~~~~~~~~~~~~~~~~~~~~~~~~~~~~~~
159494961oke
159496557oke
159497093oke
159496622oke
159498117oke  ATGTAAATTATATTTTTAGAATTTAATAAAAATTTAATAATTAATTTTTTTTTT~~AAAACTAAGAAAAGTCTTTGGAAATTTATTTTTT
159494290oke  ATGTAAATTATATTTTTAGAATTTAATAAAAATTTAATAATTAATTTTTTTTTT~~AAAACTAAGAAAAGTCTTTGGAAATTTATTTTTT
159495636oke  AAAAATTTAATTTAGA~~
159497178oke  ATGTAAATTATATTTTTAGAATTTAATAAAAATTTAATAATTAATTTTTTTT~~~~AAAACTAAGAAAAGTCTTTGGAAATTTATTTTTT
159498304oke  ATGTAAATTATATTTTTAGAATTTAATAAAAATTTAATAATTAATTTTTTTTTTTTAAAACTAAGAAAAGTCTTTGGAAATTTATTTTTT
159496326oke  ATGTAAATTATATTTTTAGAATTTAATAAAAATTTAATAATTAATTTTTTTTTTT~AAA-CTAAGAAAAGTCTTTGGAAATTTATTTTTT
159495404oke  ATGTAAATTATATTTTTAGAATTTAATAAAAATTTAATAATTAATTTTTTTTTT~~AAAACTAAGAAAAGTCTTTGGAAATTTATTTTTT
159493141oke  ~~~~~~~~~~~~~~~~~~~~~~~~~~~TACGGCCGGGTAATTAATTTTTTTTTTTA~AAACTAAGAAAAGTCTTTGGAAATTTATTTTTT
159493570oke  ~~~~~~~~~~~~~~~~~~~~~~~~~~~~~~~~~~~~~~~~~~~~~~~~~~~~~~~~~~~~~~~~~~~~~~~~~~~~~~~~~~~~~~~~~~
159493499oke  ~~~~~~~~~~~~~~~~~~~~~~~~~~~~~~~~~~~~~~~~~~~~~~~~~~~~~~~~~~~~~~~~~~~~~~~~~~~~~~~~~~~~~~~~~~
159493217oke  ~~~~~~~~~~~~~~~~~~~~~~~~~~~~~~~~~~~~~~~~~~~~~~~~~~~~~~~~~~~~~~~~~~~~~~~~~~~~~~~~~~~~~~~~~~
159493216oke  ~~~~~~~~~~~~~~~~~~~~~~~~~~~~~~~~~~~~~~~~~~~~~~~~~~~~~~~~~~~~~~~~~~~~~~~~~~~~~~~~~~~~~~~~~~
159493234oke  ~~~~~~~~~~~~~~~~~~~~~~~~~~~~~~~~~~~~~~~~~~~~~~~~~~~~~~~~~~~~~~~~~~~~~~~~~~~~~~~~~~~~~~~~~~
159492908oke  ~~~~~~~~~~~~~~~~~~~~~~~~~~~~~~~~~~~~~~~~~~~~~~~~~~~~~~~~~~~~~~~~~~~~~~~~~~~~~~~~~~~~~~~~~~
159491934oke  ~~~~~~~~~~~~~~~~~~~~~~~~~~~~~~~~~~~~~~~~~~~~~~~~~~~~~~~~~~~~~~~~~~~~~~~~~~~~~~~~~~~~~~~~~~
159492538     ~~~~~~~~~~~~~~~~~~~~~~~~~~~~~~~~~~~~~~~~~~~~~~~~~~~~~~~~~~~~~~~~~~~~~~~~~~~~~~~~~~~~~~~~~~
159498161     ~~~~~~~~~~~~~~~~~~~~~~~~~~~~~~~~~~~~~~~~~~~~~~~~~~~~~~~~~~~~~~~~~~~~~~~~~~~~~~~~~~~~~~~~~~
159493124     ~~~~~~~~~~~~~~~~~~~~~~~~~~~~~~~~~~~~~~~~~~~~~~~~~~~~~~~~~~~~~~~~~~~~~~~~~~~~~~~~~~~~~~~~~~
159493661     ~~~~~~~~~~~~~~~~~~~~~~~~~~~~~~~~~~~~~~~~~~~~~~~~~~~~~~~~~~~~~~~~~~~~~~~~~~~~~~~~~~~~~~~~~~
159494727     ~~~~~~~~~~~~~~~~~~~~~~~~~~~~~~~~~~~~~~~~~~~~~~~~~~~~~~~~~~~~~~~~~~~~~~~~~~~~~~~~~~~~~~~~~~
159496944     ~~~~~~~~~~~~~~~~~~~~~~~~~~~~~~~~~~~~~~~~~~~~~~~~~~~~~~~~~~~~~~~~~~~~~~~~~~~~~~~~~~~~~~~~~~
159494492     ~~~~~~~~~~~~~~~~~~~~~~~~~~~~~~~~~~~~~~~~~~~~~~~~~~~~~~~~~~~~~~~~~~~~~~~~~~~~~~~~~~~~~~~~~~
159496140     ~~~~~~~~~~~~~~~~~~~~~~~~~~~~~~~~~~~~~~~~~~~~~~~~~~~~~~~~~~~~~~~~~~~~~~~~~~~~~~~~~~~~~~~~~~
159497160     ~~~~~~~~~~~~~~~~~~~~~~~~~~~~~~~~~~~~~~~~~~~~~~~~~~~~~~~~~~~~~~~~~~~~~~~~~~~~~~~~~~~~~~~~~~
aaa07c02.g1   ~~~~~~~~~~~~~~~~~~~~~~~~~~~~~~~~~~~~~~~~~~~~~~~~~~~~~~~~~~~~~~~~~~~~~~~~~~~~~~~~~~~~~~~~~~

                   1270      1280      1290      1300      1310      1320      1330      1340      1350
              ....|....|....|....|....|....|....|....|....|....|....|....|....|....|....|....|....|....|
rsmitogen     ataaatattataaaaattttttttggaataagatttaataaaaaaattaaactttgaaatattaattaaaatttcttcagagttaacagg
rscox2
rstrna-his
rs16s         ataaatattataaaaattttttttggaataagatttaataaaaaaattaaactttgaaatattaattaaaatttcttcagagttaacagg
rsnad3        ~~~~~~~~~~~~~~~~~~~~~~~~~~~~~~~~~~~~~~~~~~~~~~~~~~~~~~~~~~~~~~~~~~~~~~~~~~~~~~~~~~~~~~~~~~
rstrna-leu    ~~~~~~~~~~~~~~~~~~~~~~~~~~~~~~~~~~~~~~~~~~~~~~~~~~~~~~~~~~~~~~~~~~~~~~~~~~~~~~~~~~~~~~~~~~
rscox3        ~~~~~~~~~~~~~~~~~~~~~~~~~~~~~~~~~~~~~~~~~~~~~~~~~~~~~~~~~~~~~~~~~~~~~~~~~~~~~~~~~~~~~~~~~~
rstrna-pro    ~~~~~~~~~~~~~~~~~~~~~~~~~~~~~~~~~~~~~~~~~~~~~~~~~~~~~~~~~~~~~~~~~~~~~~~~~~~~~~~~~~~~~~~~~~
rstrna-arg    ~~~~~~~~~~~~~~~~~~~~~~~~~~~~~~~~~~~~~~~~~~~~~~~~~~~~~~~~~~~~~~~~~~~~~~~~~~~~~~~~~~~~~~~~~~
rsnad6        ~~~~~~~~~~~~~~~~~~~~~~~~~~~~~~~~~~~~~~~~~~~~~~~~~~~~~~~~~~~~~~~~~~~~~~~~~~~~~~~~~~~~~~~~~~
```

```
rsnad4L       ~~~~~~~~~~~~~~~~~~~~~~~~~~~~~~~~~~~~~~~~~~~~~~~~~~~~~~~~~~~~~~~~~~~~~~~~~~~~~~~~~~~~~~~~~~
rstrna-val    ~~~~~~~~~~~~~~~~~~~~~~~~~~~~~~~~~~~~~~~~~~~~~~~~~~~~~~~~~~~~~~~~~~~~~~~~~~~~~~~~~~~~~~~~~~
rstrna-leu    ~~~~~~~~~~~~~~~~~~~~~~~~~~~~~~~~~~~~~~~~~~~~~~~~~~~~~~~~~~~~~~~~~~~~~~~~~~~~~~~~~~~~~~~~~~
159494961oke
159496557oke
159497093oke
159496622oke
159498117oke  ATAAATATTATAAAAATTTTTTT~GGAATAAGATTTAATAAAAAAATTAAACTTTGAAATA~~
159494290oke  ATAAATATTATAAAAATTTTTTTTGGAATAAGATTTAATGAAAAAAAAAAAAAAAAAA~~
159495636oke
159497178oke  ATAAATATTATAAAAATTTTTTTTGGAATAAGATTTAACA~~
159498304oke  ATAAATATTATAAAAATTTTTTT~GGAATAAGATTTAATA~
159496326oke  ATAAATATTATAAAAATTTTTTTTGGAATAAGATTTAATAAAAAAATTAAACTTTGAAATATTAATTAAAATTTCTTCAGAGTTAACAGG
159495404oke  ATAAATATTATAAAAATTTTTTTTGGAATAAGATTTAATAAAAAAATTAAACTTTGAAATATTAATTAAAATTTCTTCAGAGTTAACAGG
159493141oke  ATAAATATTGTAAAAATTTTTTTTGGAATAAGATTTAATAAAAAAATTAAACTTTGAAATATTAATTAAAATTTCTTCGGAGTTAACAGG
159493570oke  ~~~~~~~~~~~~~~~~~~~~~~~~~~~~~~~~~~~~~~~~~GGGCGGGAAACTTTGAAATATTACTTAAAATTTCTTCAGAGTTAACAGG
159493499oke  ~~~~~~~~~~~~~~~~~~~~~~~~~~~~~~~~~~~~~~~~~GGCCGGGAAACTTTGAAATATTAATTAAAATTTCTTCAGAGTTAACAGG
159493217oke  ~~~~~~~~~~~~~~~~~~~~~~~~~~~~~~~~~~~~~~~~~GGCCGGGAAACTTTGAAATATTAATTAAAATTTCTTCAGAGTTAACAGG
159493216oke  ~~~~~~~~~~~~~~~~~~~~~~~~~~~~~~~~~~~~~~~~~GGGCGGGAGAGTTTGAAATATTAATTAGAATTTCTTCAGAGTTAACAGG
159493234oke  ~~~~~~~~~~~~~~~~~~~~~~~~~~~~~~~~~~~~~~~~~~GGCCGGGCGGGAGCTTCTCGTGTTGTACGTCTCATCGGAACTAGACAG
159492908oke  ~~~~~~~~~~~~~~~~~~~~~~~~~~~~~~~~~~~~~~~~~~~~~~~~~~~~~~~~~~~~~~~~~~~~~~~~~~~~~~~~~~~~~~~~~~
159491934oke  ~~~~~~~~~~~~~~~~~~~~~~~~~~~~~~~~~~~~~~~~~~~~~~~~~~~~~~~~~~~~~~~~~~~~~~~~~~~~~~~~~~~~~~~~~~
159492538     ~~~~~~~~~~~~~~~~~~~~~~~~~~~~~~~~~~~~~~~~~~~~~~~~~~~~~~~~~~~~~~~~~~~~~~~~~~~~~~~~~~~~~~~~~~
159498161     ~~~~~~~~~~~~~~~~~~~~~~~~~~~~~~~~~~~~~~~~~~~~~~~~~~~~~~~~~~~~~~~~~~~~~~~~~~~~~~~~~~~~~~~~~~
159493124     ~~~~~~~~~~~~~~~~~~~~~~~~~~~~~~~~~~~~~~~~~~~~~~~~~~~~~~~~~~~~~~~~~~~~~~~~~~~~~~~~~~~~~~~~~~
159493661     ~~~~~~~~~~~~~~~~~~~~~~~~~~~~~~~~~~~~~~~~~~~~~~~~~~~~~~~~~~~~~~~~~~~~~~~~~~~~~~~~~~~~~~~~~~
159494727     ~~~~~~~~~~~~~~~~~~~~~~~~~~~~~~~~~~~~~~~~~~~~~~~~~~~~~~~~~~~~~~~~~~~~~~~~~~~~~~~~~~~~~~~~~~
159496944     ~~~~~~~~~~~~~~~~~~~~~~~~~~~~~~~~~~~~~~~~~~~~~~~~~~~~~~~~~~~~~~~~~~~~~~~~~~~~~~~~~~~~~~~~~~
159494492     ~~~~~~~~~~~~~~~~~~~~~~~~~~~~~~~~~~~~~~~~~~~~~~~~~~~~~~~~~~~~~~~~~~~~~~~~~~~~~~~~~~~~~~~~~~
159496140     ~~~~~~~~~~~~~~~~~~~~~~~~~~~~~~~~~~~~~~~~~~~~~~~~~~~~~~~~~~~~~~~~~~~~~~~~~~~~~~~~~~~~~~~~~~
159497160     ~~~~~~~~~~~~~~~~~~~~~~~~~~~~~~~~~~~~~~~~~~~~~~~~~~~~~~~~~~~~~~~~~~~~~~~~~~~~~~~~~~~~~~~~~~
aaa07c02.g1   ~~~~~~~~~~~~~~~~~~~~~~~~~~~~~~~~~~~~~~~~~~GGCCGGGCGGGAGCTTCTCGTGTTGTACGTCTCATCGGAACTAGACAG

                        1360      1370      1380      1390      1400      1410      1420      1430      1440
              ....|....|....|....|....|....|....|....|....|....|....|....|....|....|....|....|....|....|
rsmitogen     atttgaatattattatttaatatttttttacttttacatcgatgttgtttttaatttaattatattaaaagaagattatatttaaaaaag
rscox2
rstrna-his
rs16s         atttgaatattattatttaatatttttttacttttacatcgatgttgtttttaatttaattatattaaaagaagattatatttaaaaaag
rsnad3        ~~~~~~~~~~~~~~~~~~~~~~~~~~~~~~~~~~~~~~~~~~~~~~~~~~~~~~~~~~~~~~~~~~~~~~~~~~~~~~~~~~~~~~~~~~
rstrna-leu    ~~~~~~~~~~~~~~~~~~~~~~~~~~~~~~~~~~~~~~~~~~~~~~~~~~~~~~~~~~~~~~~~~~~~~~~~~~~~~~~~~~~~~~~~~~
rscox3        ~~~~~~~~~~~~~~~~~~~~~~~~~~~~~~~~~~~~~~~~~~~~~~~~~~~~~~~~~~~~~~~~~~~~~~~~~~~~~~~~~~~~~~~~~~
rstrna-pro    ~~~~~~~~~~~~~~~~~~~~~~~~~~~~~~~~~~~~~~~~~~~~~~~~~~~~~~~~~~~~~~~~~~~~~~~~~~~~~~~~~~~~~~~~~~
rstrna-arg    ~~~~~~~~~~~~~~~~~~~~~~~~~~~~~~~~~~~~~~~~~~~~~~~~~~~~~~~~~~~~~~~~~~~~~~~~~~~~~~~~~~~~~~~~~~
rsnad6        ~~~~~~~~~~~~~~~~~~~~~~~~~~~~~~~~~~~~~~~~~~~~~~~~~~~~~~~~~~~~~~~~~~~~~~~~~~~~~~~~~~~~~~~~~~
rsnad4L       ~~~~~~~~~~~~~~~~~~~~~~~~~~~~~~~~~~~~~~~~~~~~~~~~~~~~~~~~~~~~~~~~~~~~~~~~~~~~~~~~~~~~~~~~~~
rstrna-val    ~~~~~~~~~~~~~~~~~~~~~~~~~~~~~~~~~~~~~~~~~~~~~~~~~~~~~~~~~~~~~~~~~~~~~~~~~~~~~~~~~~~~~~~~~~
rstrna-leu    ~~~~~~~~~~~~~~~~~~~~~~~~~~~~~~~~~~~~~~~~~~~~~~~~~~~~~~~~~~~~~~~~~~~~~~~~~~~~~~~~~~~~~~~~~~
159494961oke
159496557oke
159497093oke
159496622oke
159498117oke
159494290oke
159495636oke
159497178oke
159498304oke
159496326oke  ATTTGAATATTATTATTTAATATTTTTTTACTTTTACATCGATGTTGTTTTTAATTTAATTATATTAAAAGAAGATTATATTTAAAAAGG
159495404oke  ATTTGAATATTATTATTTAATATTTTTTTACTTTTACATCGATGTTGTTTTTAATTTAATTATATTAAAAGAAGATTATATTTAAAAAAG
159493141oke  ATTTGAATATTATTATTTAATATTTTTTTACTTTTACATCGATGTTGTTTTTAATTTAATTATATTAAAAGAAGATTATATTTAAAAAAG
159493570oke  ATTTAAATATTATTATTTAATATTTTTTTACTTTTACATCCCTGTTGTTTTTAATTTAATTATATTAAAAGAAAATTATATTTAAAAAAG
159493499oke  ATTTGAATATTATTATTTAATATTTTTTTACTTTTACATCGCTGTTGTTTTTAATTTAATTATATTAAAAGAAGATTATATTTAAAAAAG
159493217oke  ATTTGAATATTATTATTTAATATTTTTTTACTTTTACATCCCTATTGTTTTTAATTTAATTATATTAAAAAAAGATTATATTTAAAAAAG
159493216oke  ATTTGAATATTATTATTTAATATTTTTTTACTTTTACATCCCTGTTGTTTTGAATTGGATTATATTAAAAGAAGATTATATTTAAAAAAG
159493234oke  ATCAAAAAACTATTATAAACTCTTTTTTTACTTCTACGTCCTGGTTAATTTTGTGTTGACTATGTTAAAAGAAGATTATATTTAGAAGAG
159492908oke  ~~~~GAATATTATTATTTAATATTTTTTTGCTTTTACATGGAGATTGTTTTTAATTTTATTTTGTTGAAAGGAGCATTATGTTTAAAAAA
159491934oke  ~~~~~~~~~~~~~~~~~~~~~~~~~~~~~~~~~~~~~~~~~~~~~~~~~~~~~~~~~~~~~~~~~~~~~~~~~~~~~~~~~~~~~~~~~~
159492538     ~~~~~~~~~~~~~~~~~~~~~~~~~~~~~~~~~~~~~~~~~~~~~~~~~~~~~~~~~~~~~~~~~~~~~~~~~~~~~~~~~~~~~~~~~~
159498161     ~~~~~~~~~~~~~~~~~~~~~~~~~~~~~~~~~~~~~~~~~~~~~~~~~~~~~~~~~~~~~~~~~~~~~~~~~~~~~~~~~~~~~~~~~~
159493124     ~~~~~~~~~~~~~~~~~~~~~~~~~~~~~~~~~~~~~~~~~~~~~~~~~~~~~~~~~~~~~~~~~~~~~~~~~~~~~~~~~~~~~~~~~~
159493661     ~~~~~~~~~~~~~~~~~~~~~~~~~~~~~~~~~~~~~~~~~~~~~~~~~~~~~~~~~~~~~~~~~~~~~~~~~~~~~~~~~~~~~~~~~~
159494727     ~~~~~~~~~~~~~~~~~~~~~~~~~~~~~~~~~~~~~~~~~~~~~~~~~~~~~~~~~~~~~~~~~~~~~~~~~~~~~~~~~~~~~~~~~~
159496944     ~~~~~~~~~~~~~~~~~~~~~~~~~~~~~~~~~~~~~~~~~~~~~~~~~~~~~~~~~~~~~~~~~~~~~~~~~~~~~~~~~~~~~~~~~~
159494492     ~~~~~~~~~~~~~~~~~~~~~~~~~~~~~~~~~~~~~~~~~~~~~~~~~~~~~~~~~~~~~~~~~~~~~~~~~~~~~~~~~~~~~~~~~~
159496140     ~~~~~~~~~~~~~~~~~~~~~~~~~~~~~~~~~~~~~~~~~~~~~~~~~~~~~~~~~~~~~~~~~~~~~~~~~~~~~~~~~~~~~~~~~~
159497160     ~~~~~~~~~~~~~~~~~~~~~~~~~~~~~~~~~~~~~~~~~~~~~~~~~~~~~~~~~~~~~~~~~~~~~~~~~~~~~~~~~~~~~~~~~~
aaa07c02.g1   ATCAAAAAACTATTATAAACTCTTTTTTTACTTCTACGTCCTGGTTAATTTTGTGTTGACTATGTTAAAAGAAGATTATATTTAGAAGAG

                        1450      1460      1470      1480      1490      1500      1510      1520      1530
              ....|....|....|....|....|....|....|....|....|....|....|....|....|....|....|....|....|....|
rsmitogen     actgttcttctattatgattaacttgaaattagtttaattcgtcgtaaggtagagttgtttatctagtttataaattttaaaaataattt
rscox2
rstrna-his
rs16s         actgttcttctattatgattaacttgaaattagtttaattcgtcgtaaggtagagttgtttatctagtttataaattttaaaaataattt
rsnad3        ~~~~~~~~~~~~~~~~~~~~~~~~~~~~~~~~~~~~~~~~~~~~~~~~~~~~~~~~~~~~~~~~~~~~~~~~~~~~~~~~~~~~~~~~~~
rstrna-leu    ~~~~~~~~~~~~~~~~~~~~~~~~~~~~~~~~~~~~~~~~~~~~~~~~~~~~~~~~~~~~~~~~~~~~~~~~~~~~~~~~~~~~~~~~~~
rscox3        ~~~~~~~~~~~~~~~~~~~~~~~~~~~~~~~~~~~~~~~~~~~~~~~~~~~~~~~~~~~~~~~~~~~~~~~~~~~~~~~~~~~~~~~~~~
rstrna-pro    ~~~~~~~~~~~~~~~~~~~~~~~~~~~~~~~~~~~~~~~~~~~~~~~~~~~~~~~~~~~~~~~~~~~~~~~~~~~~~~~~~~~~~~~~~~
rstrna-arg    ~~~~~~~~~~~~~~~~~~~~~~~~~~~~~~~~~~~~~~~~~~~~~~~~~~~~~~~~~~~~~~~~~~~~~~~~~~~~~~~~~~~~~~~~~~
rsnad6        ~~~~~~~~~~~~~~~~~~~~~~~~~~~~~~~~~~~~~~~~~~~~~~~~~~~~~~~~~~~~~~~~~~~~~~~~~~~~~~~~~~~~~~~~~~
rsnad4L       ~~~~~~~~~~~~~~~~~~~~~~~~~~~~~~~~~~~~~~~~~~~~~~~~~~~~~~~~~~~~~~~~~~~~~~~~~~~~~~~~~~~~~~~~~~
rstrna-val    ~~~~~~~~~~~~~~~~~~~~~~~~~~~~~~~~~~~~~~~~~~~~~~~~~~~~~~~~~~~~~~~~~~~~~~~~~~~~~~~~~~~~~~~~~~
rstrna-leu    ~~~~~~~~~~~~~~~~~~~~~~~~~~~~~~~~~~~~~~~~~~~~~~~~~~~~~~~~~~~~~~~~~~~~~~~~~~~~~~~~~~~~~~~~~~
159494961oke
159496557oke
159497093oke
159496622oke
159498117oke
159494290oke
159495636oke
159497178oke
159498304oke
159496326oke  ACTGTTCTTCTATTATGATTAACCTGAAATTAGTTTAATTCGTCGTAAGGTAGAGTTGTTTATCTAGTTTATAAATTTTAAAAATAATTT
159495404oke  ACTGTTCTTCTATTATGATTAACTTGAAATTAGTTTAATTCGTCGTAAGGTAGAGTTGTTTATCTAGTTTATAAATTTTAAAAATAATTT
159493141oke  ACTGTTCTTCTATTATGATTAGCCCGAAATTAGTTTAATTCGTCGTAAGGTAGAGTTGTTTATCTAGTTTATAAATTTTAAAAATAATCT
159493570oke  ACTGTTCTTCTATTATGATTAACTTGAAATTACTTTAATTCGTCGTAAGGTAGAGTTGTTTATCTAGTTTATAAATTTTAAAAATAATTT
159493499oke  ACTGTTCTTCTATTATGATTAACTTGAAATTAGTTTAATTCGTCGTAAGGTAGAGTTGTTTATCTAGTTTATAAATTTTAAAAATAATTT
159493217oke  ACTGTTCTTCTATTATGATTAACTTGAAATTAGTTTAATTCGTCGTAAGGTAGAGTTGTTTATCTAGTTTATAAATTTTAAAAATAATTT
159493216oke  ACTGTTCTTCTATTATGATTAACTTGAAATTAGTTTAATTCGTCGTAAGGTAGAGTTGTTTATCTAGTTTATAAATTTTAAAAATAATTT
159493234oke  ACTGTTCTTCTATTATCATTTACTTGAGATTAGTTTTATTCGTCGTAACGTGGAGTTGTTTATCTAGTTTATAAATTTTAAAAATAATTT
159492908oke  GGCTGTTCTTAAAATGAGATTAACCCGTAATTAGTTTAATTGGTGGTAAGGTAGAGTGGTTTA~~
159491934oke  ~~~~~~~~~~~~~~~~~~~~~~~~~~~~~~~~~~~~~~~~~~~~~~~~~~~~~~~~~~~~~~~~~~~~~~~~~~~~~~~~~~~~~~~~~~
159492538     ~~~~~~~~~~~~~~~~~~~~~~~~~~~~~~~~~~~~~~~~~~~~~~~~~~~~~~~~~~~~~~~~~~~~~~~~~~~~~~~~~~~~~~~~~~
159498161     ~~~~~~~~~~~~~~~~~~~~~~~~~~~~~~~~~~~~~~~~~~~~~~~~~~~~~~~~~~~~~~~~~~~~~~~~~~~~~~~~~~~~~~~~~~
159493124     ~~~~~~~~~~~~~~~~~~~~~~~~~~~~~~~~~~~~~~~~~~~~~~~~~~~~~~~~~~~~~~~~~~~~~~~~~~~~~~~~~~~~~~~~~~
159493661     ~~~~~~~~~~~~~~~~~~~~~~~~~~~~~~~~~~~~~~~~~~~~~~~~~~~~~~~~~~~~~~~~~~~~~~~~~~~~~~~~~~~~~~~~~~
159494727     ~~~~~~~~~~~~~~~~~~~~~~~~~~~~~~~~~~~~~~~~~~~~~~~~~~~~~~~~~~~~~~~~~~~~~~~~~~~~~~~~~~~~~~~~~~
159496944     ~~~~~~~~~~~~~~~~~~~~~~~~~~~~~~~~~~~~~~~~~~~~~~~~~~~~~~~~~~~~~~~~~~~~~~~~~~~~~~~~~~~~~~~~~~
159494492     ~~~~~~~~~~~~~~~~~~~~~~~~~~~~~~~~~~~~~~~~~~~~~~~~~~~~~~~~~~~~~~~~~~~~~~~~~~~~~~~~~~~~~~~~~~
159496140     ~~~~~~~~~~~~~~~~~~~~~~~~~~~~~~~~~~~~~~~~~~~~~~~~~~~~~~~~~~~~~~~~~~~~~~~~~~~~~~~~~~~~~~~~~~
159497160     ~~~~~~~~~~~~~~~~~~~~~~~~~~~~~~~~~~~~~~~~~~~~~~~~~~~~~~~~~~~~~~~~~~~~~~~~~~~~~~~~~~~~~~~~~~
aaa07c02.g1   ACTGTTCTTCTATTATCATTTACTTGAGATTAGTTTTATTCGTCGTAACGTGGAGTTGTTTATCTAGTTTATAAATTTTAAAAATAATTT

                        1540      1550      1560      1570      1580      1590      1600      1610      1620
              ....|....|....|....|....|....|....|....|....|....|....|....|....|....|....|....|....|....|
rsmitogen     cctgtacgaaaggaaagaaattaagattaaatttttattattatagttttatttatttaattattaataattttaattattttattatta
rscox2
rstrna-his
rs16s         cctgtacgaaaggaaagaaattaagattaaatttttattatt~
rsnad3        ~~~~~~~~~~~~~~~~~~~~~~~~~~~~~~~~~~~~~~~~~~atagttttatttatttaattattaataattttaattattttattatta
rstrna-leu    ~~~~~~~~~~~~~~~~~~~~~~~~~~~~~~~~~~~~~~~~~~~~~~~~~~~~~~~~~~~~~~~~~~~~~~~~~~~~~~~~~~~~~~~~~~
rscox3        ~~~~~~~~~~~~~~~~~~~~~~~~~~~~~~~~~~~~~~~~~~~~~~~~~~~~~~~~~~~~~~~~~~~~~~~~~~~~~~~~~~~~~~~~~~
rstrna-pro    ~~~~~~~~~~~~~~~~~~~~~~~~~~~~~~~~~~~~~~~~~~~~~~~~~~~~~~~~~~~~~~~~~~~~~~~~~~~~~~~~~~~~~~~~~~
rstrna-arg    ~~~~~~~~~~~~~~~~~~~~~~~~~~~~~~~~~~~~~~~~~~~~~~~~~~~~~~~~~~~~~~~~~~~~~~~~~~~~~~~~~~~~~~~~~~
rsnad6        ~~~~~~~~~~~~~~~~~~~~~~~~~~~~~~~~~~~~~~~~~~~~~~~~~~~~~~~~~~~~~~~~~~~~~~~~~~~~~~~~~~~~~~~~~~
rsnad4L       ~~~~~~~~~~~~~~~~~~~~~~~~~~~~~~~~~~~~~~~~~~~~~~~~~~~~~~~~~~~~~~~~~~~~~~~~~~~~~~~~~~~~~~~~~~
rstrna-val    ~~~~~~~~~~~~~~~~~~~~~~~~~~~~~~~~~~~~~~~~~~~~~~~~~~~~~~~~~~~~~~~~~~~~~~~~~~~~~~~~~~~~~~~~~~
rstrna-leu    ~~~~~~~~~~~~~~~~~~~~~~~~~~~~~~~~~~~~~~~~~~~~~~~~~~~~~~~~~~~~~~~~~~~~~~~~~~~~~~~~~~~~~~~~~~
159494961oke
159496557oke
159497093oke
159496622oke
159498117oke
159494290oke
159495636oke
159497178oke
159498304oke
159496326oke  CCTGTACAAAGA~~
159495404oke  CCTGTACGA~~
159493141oke  CCTGTACAAAAAAAAAAAAAAAAAAACCCAAAAAGAGCATGTCGGCCGCCT~~
159493570oke  CCTGTACGAAAGGAAAGAAATTAAGATTAAATTTTTATTATTATAGTTTTATTTATTTAATTATTAATAATTTTAATTATTTTATTATTA
159493499oke  CCTGTACGAAAGGAAAGAAATTAAGATTAAATTTTTATTATTATAGTTTTATTTATTTAATTATTAATAATTTTAATTATTTTATTATTA
159493217oke  CCTGTACGAAAGGAAAGAAATTAAGATTAAATTTTTATTATTATAGTTTTATTTATTTAATTATTAATAATTTTAATTATTTTATTATTA
159493216oke  CCTGTACGAAAGGAAAGAAATTAAGATTAAATTTTTATTATTATAGTTTTATTTATTTAATTATTAATAATTTTAATTATTTTATTATTA
159493234oke  CCTGTACGAAAGGAAAGAAATTAATATTATTTTTTTATTATTATAGTTTTATTTATTTAATTATTAATAAT~~
159492908oke
159491934oke  ~~~~~~~~~~~~~~~~~~~~~~~~~~~~~~~~~~~~~~~~~~~~~~~~~~~~~~~~~~~~~~~~~~~~~~~~~~~~~~~~~~~~~~~~~~
159492538     ~~~~~~~~~~~~~~~~~~~~~~~~~~~~~~~~~~~~~~~~~~~~~~~~~~~~~~~~~~~~~~~~~~~~~~~~~~~~~~~~~~~~~~~~~~
159498161     ~~~~~~~~~~~~~~~~~~~~~~~~~~~~~~~~~~~~~~~~~~~~~~~~~~~~~~~~~~~~~~~~~~~~~~~~~~~~~~~~~~~~~~~~~~
159493124     ~~~~~~~~~~~~~~~~~~~~~~~~~~~~~~~~~~~~~~~~~~~~~~~~~~~~~~~~~~~~~~~~~~~~~~~~~~~~~~~~~~~~~~~~~~
159493661     ~~~~~~~~~~~~~~~~~~~~~~~~~~~~~~~~~~~~~~~~~~~~~~~~~~~~~~~~~~~~~~~~~~~~~~~~~~~~~~~~~~~~~~~~~~
159494727     ~~~~~~~~~~~~~~~~~~~~~~~~~~~~~~~~~~~~~~~~~~~~~~~~~~~~~~~~~~~~~~~~~~~~~~~~~~~~~~~~~~~~~~~~~~
159496944     ~~~~~~~~~~~~~~~~~~~~~~~~~~~~~~~~~~~~~~~~~~~~~~~~~~~~~~~~~~~~~~~~~~~~~~~~~~~~~~~~~~~~~~~~~~
159494492     ~~~~~~~~~~~~~~~~~~~~~~~~~~~~~~~~~~~~~~~~~~~~~~~~~~~~~~~~~~~~~~~~~~~~~~~~~~~~~~~~~~~~~~~~~~
159496140     ~~~~~~~~~~~~~~~~~~~~~~~~~~~~~~~~~~~~~~~~~~~~~~~~~~~~~~~~~~~~~~~~~~~~~~~~~~~~~~~~~~~~~~~~~~
159497160     ~~~~~~~~~~~~~~~~~~~~~~~~~~~~~~~~~~~~~~~~~~~~~~~~~~~~~~~~~~~~~~~~~~~~~~~~~~~~~~~~~~~~~~~~~~
aaa07c02.g1   CCTGTACGAAAGGAAAGAAATTAATATTATTTTTTTATTATTATAGTTTTATTTATTTAATTATTAATAAT

                        1630      1640      1650      1660      1670      1680      1690      1700      1710
              ....|....|....|....|....|....|....|....|....|....|....|....|....|....|....|....|....|....|
rsmitogen     ttattattttttttgaattttttttttattgattaattttttaagtttaaataaattaatgatttttgaaagtggatttatgtctttagga
rscox2
rstrna-his
rs16s
rsnad3        ttattattttttttgaattttttttttattgattaattttttaagtttaaataaattaatgatttttgaaagtggatttatgtctttagga
rstrna-leu    ~~~~~~~~~~~~~~~~~~~~~~~~~~~~~~~~~~~~~~~~~~~~~~~~~~~~~~~~~~~~~~~~~~~~~~~~~~~~~~~~~~~~~~~~~~
rscox3        ~~~~~~~~~~~~~~~~~~~~~~~~~~~~~~~~~~~~~~~~~~~~~~~~~~~~~~~~~~~~~~~~~~~~~~~~~~~~~~~~~~~~~~~~~~
rstrna-pro    ~~~~~~~~~~~~~~~~~~~~~~~~~~~~~~~~~~~~~~~~~~~~~~~~~~~~~~~~~~~~~~~~~~~~~~~~~~~~~~~~~~~~~~~~~~
rstrna-arg    ~~~~~~~~~~~~~~~~~~~~~~~~~~~~~~~~~~~~~~~~~~~~~~~~~~~~~~~~~~~~~~~~~~~~~~~~~~~~~~~~~~~~~~~~~~
rsnad6        ~~~~~~~~~~~~~~~~~~~~~~~~~~~~~~~~~~~~~~~~~~~~~~~~~~~~~~~~~~~~~~~~~~~~~~~~~~~~~~~~~~~~~~~~~~
rsnad4L       ~~~~~~~~~~~~~~~~~~~~~~~~~~~~~~~~~~~~~~~~~~~~~~~~~~~~~~~~~~~~~~~~~~~~~~~~~~~~~~~~~~~~~~~~~~
rstrna-val    ~~~~~~~~~~~~~~~~~~~~~~~~~~~~~~~~~~~~~~~~~~~~~~~~~~~~~~~~~~~~~~~~~~~~~~~~~~~~~~~~~~~~~~~~~~
```

```
rstrna-leu       ~~~~~~~~~~~~~~~~~~~~~~~~~~~~~~~~~~~~~~~~~~~~~~~~~~~~~~~~~~~~~~~~~~~~~~~~~~~~~~~~~~~~~~~~~~
159494961oke
159496557oke
159497093oke
159496622oke
159498117oke
159494290oke
159495636oke
159497178oke
159498304oke
159496326oke
159495404oke
159493141oke
159493570oke     TTATTATTTTTTTTGAATTTTTTTTTTATTGATT~~
159493499oke     TTATTATTTTTTTTGAATTTTTTTTTTATTGATTAATTTTTTAAGTTTAAATAAATTAATGATTTTTGAAAGTGGATTTATGTCTTTAACA
159493217oke     TTATTATTTTTTTTGAATTTTTTTTTTATTGATTAATTTTTTAAGTTTAAATAAATTAATGATTTTTGAAAGTGGATTTATGT~~
159493216oke     TTATTATTTTTTTTGAATTTTTTTTTTATTGATTAATTTTTTAAGTTTAAATAAATTAATGATTTTTGAAAGTGGATTTATGTCTTTAACA
159493234oke
159492908oke
159491934oke     ~~~~~~~~~~~~~~~~~~~~~~~~~~~~~~~~~~~~~~~~~~~~~~~~~~~~~~~~~~~~~~~~~~~~~~~~~~~~~~~~~~~~~~~~~~
159492538        ~~~~~~~~~~~~~~~~~~~~~~~~~~~~~~~~~~~~~~~~~~~~~~~~~~~~~~~~~~~~~~~~~~~~~~~~~~~~~~~~~~~~~~~~~~
159498161        ~~~~~~~~~~~~~~~~~~~~~~~~~~~~~~~~~~~~~~~~~~~~~~~~~~~~~~~~~~~~~~~~~~~~~~~~~~~~~~~~~~~~~~~~~~
159493124        ~~~~~~~~~~~~~~~~~~~~~~~~~~~~~~~~~~~~~~~~~~~~~~~~~~~~~~~~~~~~~~~~~~~~~~~~~~~~~~~~~~~~~~~~~~
159493661        ~~~~~~~~~~~~~~~~~~~~~~~~~~~~~~~~~~~~~~~~~~~~~~~~~~~~~~~~~~~~~~~~~~~~~~~~~~~~~~~~~~~~~~~~~~
159494727        ~~~~~~~~~~~~~~~~~~~~~~~~~~~~~~~~~~~~~~~~~~~~~~~~~~~~~~~~~~~~~~~~~~~~~~~~~~~~~~~~~~~~~~~~~~
159496944        ~~~~~~~~~~~~~~~~~~~~~~~~~~~~~~~~~~~~~~~~~~~~~~~~~~~~~~~~~~~~~~~~~~~~~~~~~~~~~~~~~~~~~~~~~~
159494492        ~~~~~~~~~~~~~~~~~~~~~~~~~~~~~~~~~~~~~~~~~~~~~~~~~~~~~~~~~~~~~~~~~~~~~~~~~~~~~~~~~~~~~~~~~~
159496140        ~~~~~~~~~~~~~~~~~~~~~~~~~~~~~~~~~~~~~~~~~~~~~~~~~~~~~~~~~~~~~~~~~~~~~~~~~~~~~~~~~~~~~~~~~~
159497160        ~~~~~~~~~~~~~~~~~~~~~~~~~~~~~~~~~~~~~~~~~~~~~~~~~~~~~~~~~~~~~~~~~~~~~~~~~~~~~~~~~~~~~~~~~~
aaa07c02.g1

                         1720      1730      1740      1750      1760      1770      1780      1790      1800
                 ....|....|....|....|....|....|....|....|....|....|....|....|....|....|....|....|....|....|
rsmitogen        aaaaattaaaattctttttctttacattttttttttatttttattatttttgttttatttgatttagaaggtttattagtattttttttt
rscox2
rstrna-his
rs16s
rsnad3           aaaaattaaaattctttttctttacattttttttttatttttattatttttgttttatttgatttagaaggtttattagtattttttttt
rstrna-leu       ~~~~~~~~~~~~~~~~~~~~~~~~~~~~~~~~~~~~~~~~~~~~~~~~~~~~~~~~~~~~~~~~~~~~~~~~~~~~~~~~~~~~~~~~~~
rscox3           ~~~~~~~~~~~~~~~~~~~~~~~~~~~~~~~~~~~~~~~~~~~~~~~~~~~~~~~~~~~~~~~~~~~~~~~~~~~~~~~~~~~~~~~~~~
rstrna-pro       ~~~~~~~~~~~~~~~~~~~~~~~~~~~~~~~~~~~~~~~~~~~~~~~~~~~~~~~~~~~~~~~~~~~~~~~~~~~~~~~~~~~~~~~~~~
rstrna-arg       ~~~~~~~~~~~~~~~~~~~~~~~~~~~~~~~~~~~~~~~~~~~~~~~~~~~~~~~~~~~~~~~~~~~~~~~~~~~~~~~~~~~~~~~~~~
rsnad6           ~~~~~~~~~~~~~~~~~~~~~~~~~~~~~~~~~~~~~~~~~~~~~~~~~~~~~~~~~~~~~~~~~~~~~~~~~~~~~~~~~~~~~~~~~~
rsnad4L          ~~~~~~~~~~~~~~~~~~~~~~~~~~~~~~~~~~~~~~~~~~~~~~~~~~~~~~~~~~~~~~~~~~~~~~~~~~~~~~~~~~~~~~~~~~
rstrna-val       ~~~~~~~~~~~~~~~~~~~~~~~~~~~~~~~~~~~~~~~~~~~~~~~~~~~~~~~~~~~~~~~~~~~~~~~~~~~~~~~~~~~~~~~~~~
rstrna-leu       ~~~~~~~~~~~~~~~~~~~~~~~~~~~~~~~~~~~~~~~~~~~~~~~~~~~~~~~~~~~~~~~~~~~~~~~~~~~~~~~~~~~~~~~~~~
159494961oke
159496557oke
159497093oke
159496622oke
159498117oke
159494290oke
159495636oke
159497178oke
159498304oke
159496326oke
159495404oke
159493141oke
159493570oke
159493499oke     ~~
159493217oke
159493216oke     ~~
159493234oke
159492908oke
159491934oke     ~~~~~~~~~~~~~~~AGGCGGCCGACATGTCCCATAAAGGGTTTTTTTTTTTTTTTTTTCGATTTAGAAGGTCTTTTAGAGTTTTTTTTT
159492538        ~~~~~~~~~~~~~~~~~~~~~~~~~~~~~~~~~~~~~~~~~~~~~~~~~~~~~~~~~~~~~~~~~~~~~~~~~~~~~~~~~~~~~~~~~~
159498161        ~~~~~~~~~~~~~~~~~~~~~~~~~~~~~~~~~~~~~~~~~~~~~~~~~~~~~~~~~~~~~~~~~~~~~~~~~~~~~~~~~~~~~~~~~~
159493124        ~~~~~~~~~~~~~~~~~~~~~~~~~~~~~~~~~~~~~~~~~~~~~~~~~~~~~~~~~~~~~~~~~~~~~~~~~~~~~~~~~~~~~~~~~~
159493661        ~~~~~~~~~~~~~~~~~~~~~~~~~~~~~~~~~~~~~~~~~~~~~~~~~~~~~~~~~~~~~~~~~~~~~~~~~~~~~~~~~~~~~~~~~~
159494727        ~~~~~~~~~~~~~~~~~~~~~~~~~~~~~~~~~~~~~~~~~~~~~~~~~~~~~~~~~~~~~~~~~~~~~~~~~~~~~~~~~~~~~~~~~~
159496944        ~~~~~~~~~~~~~~~~~~~~~~~~~~~~~~~~~~~~~~~~~~~~~~~~~~~~~~~~~~~~~~~~~~~~~~~~~~~~~~~~~~~~~~~~~~
159494492        ~~~~~~~~~~~~~~~~~~~~~~~~~~~~~~~~~~~~~~~~~~~~~~~~~~~~~~~~~~~~~~~~~~~~~~~~~~~~~~~~~~~~~~~~~~
159496140        ~~~~~~~~~~~~~~~~~~~~~~~~~~~~~~~~~~~~~~~~~~~~~~~~~~~~~~~~~~~~~~~~~~~~~~~~~~~~~~~~~~~~~~~~~~
159497160        ~~~~~~~~~~~~~~~~~~~~~~~~~~~~~~~~~~~~~~~~~~~~~~~~~~~~~~~~~~~~~~~~~~~~~~~~~~~~~~~~~~~~~~~~~~
aaa07c02.g1

                         1810      1820      1830      1840      1850      1860      1870      1880      1890
                 ....|....|....|....|....|....|....|....|....|....|....|....|....|....|....|....|....|....|
rsmitogen        atataaactttataaaatttttttataatttttattgtagttatattaattattttttatggttaaataattgaatgaaagtttaataaa
rscox2
rstrna-his
rs16s
rsnad3           atataaactttataaaatttttttataatttttattgtagttatattaattattttttatggttaaataattgaatgaaagtttaataaa
rstrna-leu       ~~~~~~~~~~~~~~~~~~~~~~~~~~~~~~~~~~~~~~~~~~~~~~~~~~~~~~~~~~~~~~~~~~~~~~~~~~~~~~~~~~~~~~~~~~
rscox3           ~~~~~~~~~~~~~~~~~~~~~~~~~~~~~~~~~~~~~~~~~~~~~~~~~~~~~~~~~~~~~~~~~~~~~~~~~~~~~~~~~~~~~~~~~~
rstrna-pro       ~~~~~~~~~~~~~~~~~~~~~~~~~~~~~~~~~~~~~~~~~~~~~~~~~~~~~~~~~~~~~~~~~~~~~~~~~~~~~~~~~~~~~~~~~~
rstrna-arg       ~~~~~~~~~~~~~~~~~~~~~~~~~~~~~~~~~~~~~~~~~~~~~~~~~~~~~~~~~~~~~~~~~~~~~~~~~~~~~~~~~~~~~~~~~~
rsnad6           ~~~~~~~~~~~~~~~~~~~~~~~~~~~~~~~~~~~~~~~~~~~~~~~~~~~~~~~~~~~~~~~~~~~~~~~~~~~~~~~~~~~~~~~~~~
rsnad4L          ~~~~~~~~~~~~~~~~~~~~~~~~~~~~~~~~~~~~~~~~~~~~~~~~~~~~~~~~~~~~~~~~~~~~~~~~~~~~~~~~~~~~~~~~~~
rstrna-val       ~~~~~~~~~~~~~~~~~~~~~~~~~~~~~~~~~~~~~~~~~~~~~~~~~~~~~~~~~~~~~~~~~~~~~~~~~~~~~~~~~~~~~~~~~~
rstrna-leu       ~~~~~~~~~~~~~~~~~~~~~~~~~~~~~~~~~~~~~~~~~~~~~~~~~~~~~~~~~~~~~~~~~~~~~~~~~~~~~~~~~~~~~~~~~~
159494961oke
159496557oke
159497093oke
159496622oke
159498117oke
159494290oke
159495636oke
159497178oke
159498304oke
159496326oke
159495404oke
159493141oke
159493570oke
159493499oke
159493217oke
159493216oke
159493234oke
159492908oke
159491934oke     ATATAAACTTTATAAAATTTTTTTATAATTTTTATTGTAGTTATATTAATTATTTTTTATGGTTAAATAATTGAATGAAAGTTTAATAAA
159492538        ~~~~~~~~~~~~~~~~~~~~~~~~~~~~~~~~~~~~~~~~~~~~~~~~~~~~~~~~~~~~~~~~~~~~~~~~~~~~~~~~~~~~~~~~~~
159498161        ~~~~~~~~~~~~~~~~~~~~~~~~~~~~~~~~~~~~~~~~~~~~~~~~~~~~~~~~~~~~~~~~~~~~~~~~~~~~~~~~~~~~~~~~~~
159493124        ~~~~~~~~~~~~~~~~~~~~~~~~~~~~~~~~~~~~~~~~~~~~~~~~~~~~~~~~~~~~~~~~~~~~~~~~~~~~~~~~~~~~~~~~~~
159493661        ~~~~~~~~~~~~~~~~~~~~~~~~~~~~~~~~~~~~~~~~~~~~~~~~~~~~~~~~~~~~~~~~~~~~~~~~~~~~~~~~~~~~~~~~~~
159494727        ~~~~~~~~~~~~~~~~~~~~~~~~~~~~~~~~~~~~~~~~~~~~~~~~~~~~~~~~~~~~~~~~~~~~~~~~~~~~~~~~~~~~~~~~~~
159496944        ~~~~~~~~~~~~~~~~~~~~~~~~~~~~~~~~~~~~~~~~~~~~~~~~~~~~~~~~~~~~~~~~~~~~~~~~~~~~~~~~~~~~~~~~~~
159494492        ~~~~~~~~~~~~~~~~~~~~~~~~~~~~~~~~~~~~~~~~~~~~~~~~~~~~~~~~~~~~~~~~~~~~~~~~~~~~~~~~~~~~~~~~~~
159496140        ~~~~~~~~~~~~~~~~~~~~~~~~~~~~~~~~~~~~~~~~~~~~~~~~~~~~~~~~~~~~~~~~~~~~~~~~~~~~~~~~~~~~~~~~~~
159497160        ~~~~~~~~~~~~~~~~~~~~~~~~~~~~~~~~~~~~~~~~~~~~~~~~~~~~~~~~~~~~~~~~~~~~~~~~~~~~~~~~~~~~~~~~~~
aaa07c02.g1

                         1900      1910      1920      1930      1940      1950      1960      1970      1980
                 ....|....|....|....|....|....|....|....|....|....|....|....|....|....|....|....|....|....|
rsmitogen        ttattatgaattattaaagattattattataaataaatatttatattttaggaatataagatttttatctaatattttaaaataattatt
rscox2
rstrna-his
rs16s
rsnad3           ttattatgaattatt~
rstrna-leu       ~~~~~~~~~~~~~~~~~~~~~~~ttattataaataaatatttatattttaggaatataagatttttatctaatattttaaaa~
rscox3           ~~~~~~~~~~~~~~~~~~~~~~~~~~~~~~~~~~~~~~~~~~~~~~~~~~~~~~~~~~~~~~~~~~~~~~~~~~~~~~~~~~~~~~~~~~
rstrna-pro       ~~~~~~~~~~~~~~~~~~~~~~~~~~~~~~~~~~~~~~~~~~~~~~~~~~~~~~~~~~~~~~~~~~~~~~~~~~~~~~~~~~~~~~~~~~
rstrna-arg       ~~~~~~~~~~~~~~~~~~~~~~~~~~~~~~~~~~~~~~~~~~~~~~~~~~~~~~~~~~~~~~~~~~~~~~~~~~~~~~~~~~~~~~~~~~
rsnad6           ~~~~~~~~~~~~~~~~~~~~~~~~~~~~~~~~~~~~~~~~~~~~~~~~~~~~~~~~~~~~~~~~~~~~~~~~~~~~~~~~~~~~~~~~~~
rsnad4L          ~~~~~~~~~~~~~~~~~~~~~~~~~~~~~~~~~~~~~~~~~~~~~~~~~~~~~~~~~~~~~~~~~~~~~~~~~~~~~~~~~~~~~~~~~~
rstrna-val       ~~~~~~~~~~~~~~~~~~~~~~~~~~~~~~~~~~~~~~~~~~~~~~~~~~~~~~~~~~~~~~~~~~~~~~~~~~~~~~~~~~~~~~~~~~
rstrna-leu       ~~~~~~~~~~~~~~~~~~~~~~~~~~~~~~~~~~~~~~~~~~~~~~~~~~~~~~~~~~~~~~~~~~~~~~~~~~~~~~~~~~~~~~~~~~
159494961oke
159496557oke
159497093oke
159496622oke
159498117oke
159494290oke
159495636oke
159497178oke
159498304oke
159496326oke
159495404oke
159493141oke
159493570oke
159493499oke
159493217oke
159493216oke
159493234oke
159492908oke
159491934oke     TTATTATGAATTATTAAAGATTATTATTATAAATAAATATTTATATTTTAGGAATATAAGATTTTTATCTAATATTTTAAAATAATTATT
159492538        ~~~~~~~~~~~~~~~~~~~~~~~~~~~~~~~~~~~~~~~~~~~~~~~~~~~~~~~~~~~~~~~~~~~~~~~~~~~~~~~~~~~~~~~~~~
159498161        ~~~~~~~~~~~~~~~~~~~~~~~~~~~~~~~~~~~~~~~~~~~~~~~~~~~~~~~~~~~~~~~~~~~~~~~~~~~~~~~~~~~~~~~~~~
159493124        ~~~~~~~~~~~~~~~~~~~~~~~~~~~~~~~~~~~~~~~~~~~~~~~~~~~~~~~~~~~~~~~~~~~~~~~~~~~~~~~~~~~~~~~~~~
159493661        ~~~~~~~~~~~~~~~~~~~~~~~~~~~~~~~~~~~~~~~~~~~~~~~~~~~~~~~~~~~~~~~~~~~~~~~~~~~~~~~~~~~~~~~~~~
159494727        ~~~~~~~~~~~~~~~~~~~~~~~~~~~~~~~~~~~~~~~~~~~~~~~~~~~~~~~~~~~~~~~~~~~~~~~~~~~~~~~~~~~~~~~~~~
159496944        ~~~~~~~~~~~~~~~~~~~~~~~~~~~~~~~~~~~~~~~~~~~~~~~~~~~~~~~~~~~~~~~~~~~~~~~~~~~~~~~~~~~~~~~~~~
159494492        ~~~~~~~~~~~~~~~~~~~~~~~~~~~~~~~~~~~~~~~~~~~~~~~~~~~~~~~~~~~~~~~~~~~~~~~~~~~~~~~~~~~~~~~~~~
159496140        ~~~~~~~~~~~~~~~~~~~~~~~~~~~~~~~~~~~~~~~~~~~~~~~~~~~~~~~~~~~~~~~~~~~~~~~~~~~~~~~~~~~~~~~~~~
159497160        ~~~~~~~~~~~~~~~~~~~~~~~~~~~~~~~~~~~~~~~~~~~~~~~~~~~~~~~~~~~~~~~~~~~~~~~~~~~~~~~~~~~~~~~~~~
aaa07c02.g1

                         1990      2000      2010      2020      2030      2040      2050      2060      2070
                 ....|....|....|....|....|....|....|....|....|....|....|....|....|....|....|....|....|....|
rsmitogen        aacaataaaatgctgtaaaccataaatatattggttctatataatttgtttttgcttttttttagaataataatgggcgtttctttaagtt
rscox2
rstrna-his
rs16s
rsnad3
rstrna-leu
rscox3           ~~~~~~~~~~~~~~~~~~~~~~~~~~~~~attggttctatataatttgtttttgcttttttttagaataataatgggcgtttctttaagtt
rstrna-pro       ~~~~~~~~~~~~~~~~~~~~~~~~~~~~~~~~~~~~~~~~~~~~~~~~~~~~~~~~~~~~~~~~~~~~~~~~~~~~~~~~~~~~~~~~~~
rstrna-arg       ~~~~~~~~~~~~~~~~~~~~~~~~~~~~~~~~~~~~~~~~~~~~~~~~~~~~~~~~~~~~~~~~~~~~~~~~~~~~~~~~~~~~~~~~~~
rsnad6           ~~~~~~~~~~~~~~~~~~~~~~~~~~~~~~~~~~~~~~~~~~~~~~~~~~~~~~~~~~~~~~~~~~~~~~~~~~~~~~~~~~~~~~~~~~
rsnad4L          ~~~~~~~~~~~~~~~~~~~~~~~~~~~~~~~~~~~~~~~~~~~~~~~~~~~~~~~~~~~~~~~~~~~~~~~~~~~~~~~~~~~~~~~~~~
rstrna-val       ~~~~~~~~~~~~~~~~~~~~~~~~~~~~~~~~~~~~~~~~~~~~~~~~~~~~~~~~~~~~~~~~~~~~~~~~~~~~~~~~~~~~~~~~~~
rstrna-leu       ~~~~~~~~~~~~~~~~~~~~~~~~~~~~~~~~~~~~~~~~~~~~~~~~~~~~~~~~~~~~~~~~~~~~~~~~~~~~~~~~~~~~~~~~~~
159494961oke
```

```
159496557oke
159497093oke
159496622oke
159498117oke
159494290oke
159495636oke
159497178oke
159498304oke
159496326oke
159495404oke
159493141oke
159493570oke
159493499oke
159493217oke
159493216oke
159493234oke
159492908oke
159491934oke  AACAATAAAATGCTGTAAACCATAAATATATTGGTTCTATATAATTTGTTTTTGCTTTTTTTAGAATAATAATGGGCGTTTCTTTAAGTT
159492538     ~~~~~~~~~~~~~~~~~~~~~~~~~~~~~~~~~~~~~~~~~~~~~~~~~~~~~~~~~~~~~~~~~~~~~~~~~~~~~~~~~~~~~~~~~~
159498161     ~~~~~~~~~~~~~~~~~~~~~~~~~~~~~~~~~~~~~~~~~~~~~~~~~~~~~~~~~~~~~~~~~~~~~~~~~~~~~~~~~~~~~~~~~~
159493124     ~~~~~~~~~~~~~~~~~~~~~~~~~~~~~~~~~~~~~~~~~~~~~~~~~~~~~~~~~~~~~~~~~~~~~~~~~~~~~~~~~~~~~~~~~~
159493661     ~~~~~~~~~~~~~~~~~~~~~~~~~~~~~~~~~~~~~~~~~~~~~~~~~~~~~~~~~~~~~~~~~~~~~~~~~~~~~~~~~~~~~~~~~~
159494727     ~~~~~~~~~~~~~~~~~~~~~~~~~~~~~~~~~~~~~~~~~~~~~~~~~~~~~~~~~~~~~~~~~~~~~~~~~~~~~~~~~~~~~~~~~~
159496944     ~~~~~~~~~~~~~~~~~~~~~~~~~~~~~~~~~~~~~~~~~~~~~~~~~~~~~~~~~~~~~~~~~~~~~~~~~~~~~~~~~~~~~~~~~~
159494492     ~~~~~~~~~~~~~~~~~~~~~~~~~~~~~~~~~~~~~~~~~~~~~~~~~~~~~~~~~~~~~~~~~~~~~~~~~~~~~~~~~~~~~~~~~~
159496140     ~~~~~~~~~~~~~~~~~~~~~~~~~~~~~~~~~~~~~~~~~~~~~~~~~~~~~~~~~~~~~~~~~~~~~~~~~~~~~~~~~~~~~~~~~~
159497160     ~~~~~~~~~~~~~~~~~~~~~~~~~~~~~~~~~~~~~~~~~~~~~~~~~~~~~~~~~~~~~~~~~~~~~~~~~~~~~~~~~~~~~~~~~~
aaa07c02.g1

                      2080      2090      2100      2110      2120      2130      2140      2150      2160
              ....|....|....|....|....|....|....|....|....|....|....|....|....|....|....|....|....|....|
rsmitogen     tattaattcgtttagaattaggtaatcctggttttttattgggtaatggacagttatttaatagtattattactggacatgcattaatta
rscox2
rstrna-his
rs16s
rsnad3
rstrna-leu
rscox3        tattaattcgtttagaattaggtaatcctggttttttattgggtaatggacagttatttaatagtattattactggacatgcattaatta
rstrna-pro    ~~~~~~~~~~~~~~~~~~~~~~~~~~~~~~~~~~~~~~~~~~~~~~~~~~~~~~~~~~~~~~~~~~~~~~~~~~~~~~~~~~~~~~~~~~
rstrna-arg    ~~~~~~~~~~~~~~~~~~~~~~~~~~~~~~~~~~~~~~~~~~~~~~~~~~~~~~~~~~~~~~~~~~~~~~~~~~~~~~~~~~~~~~~~~~
rsnad6        ~~~~~~~~~~~~~~~~~~~~~~~~~~~~~~~~~~~~~~~~~~~~~~~~~~~~~~~~~~~~~~~~~~~~~~~~~~~~~~~~~~~~~~~~~~
rsnad4L       ~~~~~~~~~~~~~~~~~~~~~~~~~~~~~~~~~~~~~~~~~~~~~~~~~~~~~~~~~~~~~~~~~~~~~~~~~~~~~~~~~~~~~~~~~~
rstrna-val    ~~~~~~~~~~~~~~~~~~~~~~~~~~~~~~~~~~~~~~~~~~~~~~~~~~~~~~~~~~~~~~~~~~~~~~~~~~~~~~~~~~~~~~~~~~
rstrna-leu    ~~~~~~~~~~~~~~~~~~~~~~~~~~~~~~~~~~~~~~~~~~~~~~~~~~~~~~~~~~~~~~~~~~~~~~~~~~~~~~~~~~~~~~~~~~
159494961oke
159496557oke
159497093oke
159496622oke
159498117oke
159494290oke
159495636oke
159497178oke
159498304oke
159496326oke
159495404oke
159493141oke
159493570oke
159493499oke
159493217oke
159493216oke
159493234oke
159492908oke
159491934oke  TATTAATTCGTTTAAAATTAGGTAATCCTGGTTTTTTATTGGGTAATGGACAGTTATTTAATAGTATTATTACTGGACATGCATTAATTA
159492538     ~~~~~~~~~~~~~~~~~~~~~~~~~~~~~~~~~~~~~~~~~~~~~~~~~~~~~~~~~~~~~~~~~~~~~~~~CTGGACATGCATTAATTA
159498161     ~~~~~~~~~~~~~~~~~~~~~~~~~~~~~~~~~~~~~~~~~~~~~~~~~~~~~~~~~~~~~~~~~~~~~~~~~~~~~~~~~~~~~~~~~~
159493124     ~~~~~~~~~~~~~~~~~~~~~~~~~~~~~~~~~~~~~~~~~~~~~~~~~~~~~~~~~~~~~~~~~~~~~~~~~~~~~~~~~~~~~~~~~~
159493661     ~~~~~~~~~~~~~~~~~~~~~~~~~~~~~~~~~~~~~~~~~~~~~~~~~~~~~~~~~~~~~~~~~~~~~~~~~~~~~~~~~~~~~~~~~~
159494727     ~~~~~~~~~~~~~~~~~~~~~~~~~~~~~~~~~~~~~~~~~~~~~~~~~~~~~~~~~~~~~~~~~~~~~~~~~~~~~~~~~~~~~~~~~~
159496944     ~~~~~~~~~~~~~~~~~~~~~~~~~~~~~~~~~~~~~~~~~~~~~~~~~~~~~~~~~~~~~~~~~~~~~~~~~~~~~~~~~~~~~~~~~~
159494492     ~~~~~~~~~~~~~~~~~~~~~~~~~~~~~~~~~~~~~~~~~~~~~~~~~~~~~~~~~~~~~~~~~~~~~~~~~~~~~~~~~~~~~~~~~~
159496140     ~~~~~~~~~~~~~~~~~~~~~~~~~~~~~~~~~~~~~~~~~~~~~~~~~~~~~~~~~~~~~~~~~~~~~~~~~~~~~~~~~~~~~~~~~~
159497160     ~~~~~~~~~~~~~~~~~~~~~~~~~~~~~~~~~~~~~~~~~~~~~~~~~~~~~~~~~~~~~~~~~~~~~~~~~~~~~~~~~~~~~~~~~~
aaa07c02.g1

                      2170      2180      2190      2200      2210      2220      2230      2240      2250
              ....|....|....|....|....|....|....|....|....|....|....|....|....|....|....|....|....|....|
rsmitogen     taattttttttttttgttataccaatattaataggtttttttggtaatatattaattccattattattaatatctgctgatatagcttgac
rscox2
rstrna-his
rs16s
rsnad3
rstrna-leu
rscox3        taattttttttttttgttataccaatattaataggtttttttggtaatatattaattccattattattaatatctgctgatatagcttgac
rstrna-pro    ~~~~~~~~~~~~~~~~~~~~~~~~~~~~~~~~~~~~~~~~~~~~~~~~~~~~~~~~~~~~~~~~~~~~~~~~~~~~~~~~~~~~~~~~~~
rstrna-arg    ~~~~~~~~~~~~~~~~~~~~~~~~~~~~~~~~~~~~~~~~~~~~~~~~~~~~~~~~~~~~~~~~~~~~~~~~~~~~~~~~~~~~~~~~~~
rsnad6        ~~~~~~~~~~~~~~~~~~~~~~~~~~~~~~~~~~~~~~~~~~~~~~~~~~~~~~~~~~~~~~~~~~~~~~~~~~~~~~~~~~~~~~~~~~
rsnad4L       ~~~~~~~~~~~~~~~~~~~~~~~~~~~~~~~~~~~~~~~~~~~~~~~~~~~~~~~~~~~~~~~~~~~~~~~~~~~~~~~~~~~~~~~~~~
rstrna-val    ~~~~~~~~~~~~~~~~~~~~~~~~~~~~~~~~~~~~~~~~~~~~~~~~~~~~~~~~~~~~~~~~~~~~~~~~~~~~~~~~~~~~~~~~~~
rstrna-leu    ~~~~~~~~~~~~~~~~~~~~~~~~~~~~~~~~~~~~~~~~~~~~~~~~~~~~~~~~~~~~~~~~~~~~~~~~~~~~~~~~~~~~~~~~~~
159494961oke
159496557oke
159497093oke
159496622oke
159498117oke
159494290oke
159495636oke
159497178oke
159498304oke
159496326oke
159495404oke
159493141oke
159493570oke
159493499oke
159493217oke
159493216oke
159493234oke
159492908oke
159491934oke  TAATTTTTTTTTTTGTTATACCAATATTAATAGGTTTTTTTGGTAATATATTAATTCCATTATTA~~
159492538     TAA~TTTTTTTTTTGTTATACCAATATTAATAGGTTTTTTTGGTAATATATTAATTCCATTATTATTAATATCTGCTGATATAGCTTGAC
159498161     ~~~TTTTTTTTTTTGTTATACCAATATTAATAGGTTTTTTGGGTAATATATTAATTCCATTATTATTAATATCTGCTGATATAGCTTGAC
159493124     ~~~~~~~~~~~~~~~~~~~~~~~~~~~~~~~~~~~~~~~~~~~~~~~~~~~~~~~~~~~~~~~~~~~~~~~~~~~~~~~~~~~~~~~~~~
159493661     ~~~~~~~~~~~~~~~~~~~~~~~~~~~~~~~~~~~~~~~~~~~~~~~~~~~~~~~~~~~~~~~~~~~~~~~~~~~~~~~~~~~~~~~~~~
159494727     ~~~~~~~~~~~~~~~~~~~~~~~~~~~~~~~~~~~~~~~~~~~~~~~~~~~~~~~~~~~~~~~~~~~~~~~~~~~~~~~~~~~~~~~~~~
159496944     ~~~~~~~~~~~~~~~~~~~~~~~~~~~~~~~~~~~~~~~~~~~~~~~~~~~~~~~~~~~~~~~~~~~~~~~~~~~~~~~~~~~~~~~~~~
159494492     ~~~~~~~~~~~~~~~~~~~~~~~~~~~~~~~~~~~~~~~~~~~~~~~~~~~~~~~~~~~~~~~~~~~~~~~~~~~~~~~~~~~~~~~~~~
159496140     ~~~~~~~~~~~~~~~~~~~~~~~~~~~~~~~~~~~~~~~~~~~~~~~~~~~~~~~~~~~~~~~~~~~~~~~~~~~~~~~~~~~~~~~~~~
159497160     ~~~~~~~~~~~~~~~~~~~~~~~~~~~~~~~~~~~~~~~~~~~~~~~~~~~~~~~~~~~~~~~~~~~~~~~~~~~~~~~~~~~~~~~~~~
aaa07c02.g1

                      2260      2270      2280      2290      2300      2310      2320      2330      2340
              ....|....|....|....|....|....|....|....|....|....|....|....|....|....|....|....|....|....|
rsmitogen     ctcgtgtaaataatcaaagtttttggtttttacctgctgctataattttaatgataataagtataattattgatcaaggttctggaacta
rscox2
rstrna-his
rs16s
rsnad3
rstrna-leu
rscox3        ctcgtgtaaataatcaaagtttttggtttttacctgctgctataattttaatgataataagtataattattgatcaaggttctggaacta
rstrna-pro    ~~~~~~~~~~~~~~~~~~~~~~~~~~~~~~~~~~~~~~~~~~~~~~~~~~~~~~~~~~~~~~~~~~~~~~~~~~~~~~~~~~~~~~~~~~
rstrna-arg    ~~~~~~~~~~~~~~~~~~~~~~~~~~~~~~~~~~~~~~~~~~~~~~~~~~~~~~~~~~~~~~~~~~~~~~~~~~~~~~~~~~~~~~~~~~
rsnad6        ~~~~~~~~~~~~~~~~~~~~~~~~~~~~~~~~~~~~~~~~~~~~~~~~~~~~~~~~~~~~~~~~~~~~~~~~~~~~~~~~~~~~~~~~~~
rsnad4L       ~~~~~~~~~~~~~~~~~~~~~~~~~~~~~~~~~~~~~~~~~~~~~~~~~~~~~~~~~~~~~~~~~~~~~~~~~~~~~~~~~~~~~~~~~~
rstrna-val    ~~~~~~~~~~~~~~~~~~~~~~~~~~~~~~~~~~~~~~~~~~~~~~~~~~~~~~~~~~~~~~~~~~~~~~~~~~~~~~~~~~~~~~~~~~
rstrna-leu    ~~~~~~~~~~~~~~~~~~~~~~~~~~~~~~~~~~~~~~~~~~~~~~~~~~~~~~~~~~~~~~~~~~~~~~~~~~~~~~~~~~~~~~~~~~
159494961oke
159496557oke
159497093oke
159496622oke
159498117oke
159494290oke
159495636oke
159497178oke
159498304oke
159496326oke
159495404oke
159493141oke
159493570oke
159493499oke
159493217oke
159493216oke
159493234oke
159492908oke
159491934oke
159492538     CTCGTGTAAATAATCAAAGTTTTTGGTTTTTACCTGCTGCTATAATTTTAATGATAATAAGTATAATTATTGATCAAGGTTCTGGAACTA
159498161     CTCGTGTAAATAATCAAAGTTTTTGGTTTTTACCTGCTGCTATAATTTTAATGATAATAAGTATAATTATTGATCAAGGTTCTGGAACTA
159493124     ~~~~~~~~~~~~~~~~~~~~~~~~~~~~~~~~~~~~~~~~~~~~~~~~~~~~~~~~~~~~~~~~~~~~~~~~~~~~~~~~~~~~~~~~~~
159493661     ~~~~~~~~~~~~~~~~~~~~~~~~~~~~~~~~~~~~~~~~~~~~~~~~~~~~~~~~~~~~~~~~~~~~~~~~~~~~~~~~~~~~~~~~~~
159494727     ~~~~~~~~~~~~~~~~~~~~~~~~~~~~~~~~~~~~~~~~~~~~~~~~~~~~~~~~~~~~~~~~~~~~~~~~~~~~~~~~~~~~~~~~~~
159496944     ~~~~~~~~~~~~~~~~~~~~~~~~~~~~~~~~~~~~~~~~~~~~~~~~~~~~~~~~~~~~~~~~~~~~~~~~~~~~~~~~~~~~~~~~~~
159494492     ~~~~~~~~~~~~~~~~~~~~~~~~~~~~~~~~~~~~~~~~~~~~~~~~~~~~~~~~~~~~~~~~~~~~~~~~~~~~~~~~~~~~~~~~~~
159496140     ~~~~~~~~~~~~~~~~~~~~~~~~~~~~~~~~~~~~~~~~~~~~~~~~~~~~~~~~~~~~~~~~~~~~~~~~~~~~~~~~~~~~~~~~~~
159497160     ~~~~~~~~~~~~~~~~~~~~~~~~~~~~~~~~~~~~~~~~~~~~~~~~~~~~~~~~~~~~~~~~~~~~~~~~~~~~~~~~~~~~~~~~~~
aaa07c02.g1

                      2350      2360      2370      2380      2390      2400      2410      2420      2430
              ....|....|....|....|....|....|....|....|....|....|....|....|....|....|....|....|....|....|
rsmitogen     gttgaactttataaccgcctttaagatcatctggtcatttggggggttctgtcgattttagtatttttagtttacatgtagccgggttta
rscox2
rstrna-his
rs16s
rsnad3
rstrna-leu
rscox3        gttgaactttataaccgcctttaagatcatctggtcatttggggggttctgtcgattttagtatttttagtttacatgtagccgggttta
rstrna-pro    ~~~~~~~~~~~~~~~~~~~~~~~~~~~~~~~~~~~~~~~~~~~~~~~~~~~~~~~~~~~~~~~~~~~~~~~~~~~~~~~~~~~~~~~~~~
rstrna-arg    ~~~~~~~~~~~~~~~~~~~~~~~~~~~~~~~~~~~~~~~~~~~~~~~~~~~~~~~~~~~~~~~~~~~~~~~~~~~~~~~~~~~~~~~~~~
rsnad6        ~~~~~~~~~~~~~~~~~~~~~~~~~~~~~~~~~~~~~~~~~~~~~~~~~~~~~~~~~~~~~~~~~~~~~~~~~~~~~~~~~~~~~~~~~~
rsnad4L       ~~~~~~~~~~~~~~~~~~~~~~~~~~~~~~~~~~~~~~~~~~~~~~~~~~~~~~~~~~~~~~~~~~~~~~~~~~~~~~~~~~~~~~~~~~
rstrna-val    ~~~~~~~~~~~~~~~~~~~~~~~~~~~~~~~~~~~~~~~~~~~~~~~~~~~~~~~~~~~~~~~~~~~~~~~~~~~~~~~~~~~~~~~~~~
rstrna-leu    ~~~~~~~~~~~~~~~~~~~~~~~~~~~~~~~~~~~~~~~~~~~~~~~~~~~~~~~~~~~~~~~~~~~~~~~~~~~~~~~~~~~~~~~~~~
159494961oke
159496557oke
159497093oke
```

```
159496622oke
159498117oke
159494290oke
159495636oke
159497178oke
159498304oke
159496326oke
159495404oke
159493141oke
159493570oke
159434499oke
159493217oke
159493216oke
159493234oke
159492908oke
159491934oke
159492538    GTTGAACTTTATAACCGCCTTTAAGATCATCTGGTCATTTGGGGGGTTCTGTCGATTTTAGTATTTTTAGTTTACATGTAGCCGGGTTTA
159498161    GTTGAACTTTATAACCGCCTTTAAGATCATCTGGTCATTTGGGGGGTTCTGTCGATTTTAGTATTTTTAGTTTACATGTAGCCGGGTTTA
159493124    ~~~~~~~~~~~~~~~~~~~~~~~~~~~~~~~~~~~~~~~~~~~~~~~~~~~~~~~~~~~~~~~~~~~~~~~~~~~~~~~~~~~~~~~~~~
159493661    ~~~~~~~~~~~~~~~~~~~~~~~~~~~~~~~~~~~~~~~~~~~~~~~~~~~~~~~~~~~~~~~~~~~~~~~~~~~~~~~~~~~~~~~~~~
159494727    ~~~~~~~~~~~~~~~~~~~~~~~~~~~~~~~~~~~~~~~~~~~~~~~~~~~~~~~~~~~~~~~~~~~~~~~~~~~~~~~~~~~~~~~~~~
159496944    ~~~~~~~~~~~~~~~~~~~~~~~~~~~~~~~~~~~~~~~~~~~~~~~~~~~~~~~~~~~~~~~~~~~~~~~~~~~~~~~~~~~~~~~~~~
159494492    ~~~~~~~~~~~~~~~~~~~~~~~~~~~~~~~~~~~~~~~~~~~~~~~~~~~~~~~~~~~~~~~~~~~~~~~~~~~~~~~~~~~~~~~~~~
159496140    ~~~~~~~~~~~~~~~~~~~~~~~~~~~~~~~~~~~~~~~~~~~~~~~~~~~~~~~~~~~~~~~~~~~~~~~~~~~~~~~~~~~~~~~~~~
159497160    ~~~~~~~~~~~~~~~~~~~~~~~~~~~~~~~~~~~~~~~~~~~~~~~~~~~~~~~~~~~~~~~~~~~~~~~~~~~~~~~~~~~~~~~~~~
aaa07c02.g1

                     2440      2450      2460      2470      2480      2490      2500      2510      2520
             ....|....|....|....|....|....|....|....|....|....|....|....|....|....|....|....|....|....|
rsmitogen    gtagattagctggttctataaatttttaagctcaataaaatctttacgtagtataacaattagtttagaaataatagatttatttttat
rscox2
rstrna-his
rs16s
rsnad3
rstrna-leu
rscox3       gtagattagctggttctataaatttttaagctcaataaaatctttacgtagtataacaattagtttagaaataatagatttatttttat
rstrna-pro   ~~~~~~~~~~~~~~~~~~~~~~~~~~~~~~~~~~~~~~~~~~~~~~~~~~~~~~~~~~~~~~~~~~~~~~~~~~~~~~~~~~~~~~~~~~
rstrna-arg   ~~~~~~~~~~~~~~~~~~~~~~~~~~~~~~~~~~~~~~~~~~~~~~~~~~~~~~~~~~~~~~~~~~~~~~~~~~~~~~~~~~~~~~~~~~
rsnad6       ~~~~~~~~~~~~~~~~~~~~~~~~~~~~~~~~~~~~~~~~~~~~~~~~~~~~~~~~~~~~~~~~~~~~~~~~~~~~~~~~~~~~~~~~~~
rsnad4L      ~~~~~~~~~~~~~~~~~~~~~~~~~~~~~~~~~~~~~~~~~~~~~~~~~~~~~~~~~~~~~~~~~~~~~~~~~~~~~~~~~~~~~~~~~~
rstrna-val   ~~~~~~~~~~~~~~~~~~~~~~~~~~~~~~~~~~~~~~~~~~~~~~~~~~~~~~~~~~~~~~~~~~~~~~~~~~~~~~~~~~~~~~~~~~
rstrna-leu   ~~~~~~~~~~~~~~~~~~~~~~~~~~~~~~~~~~~~~~~~~~~~~~~~~~~~~~~~~~~~~~~~~~~~~~~~~~~~~~~~~~~~~~~~~~
159494961oke
159496557oke
159497093oke
159496622oke
159498117oke
159494290oke
159495636oke
159497178oke
159498304oke
159496326oke
159495404oke
159493141oke
159493570oke
159434499oke
159493217oke
159493216oke
159493234oke
159492908oke
159491934oke
159492538    GTAGATTAGCTGGTTCTATAAATTTTTAAGCTCAATAAAATCTTTACGTAGTATAACAATTAGTTTAGAAATAATAGATTTATTTTTAT
159498161    GTAGATTAGCTGGTTCTATAAATTTTTAAGCTCAATAAAATCTTTACGTAGTATAACAATTAGTTTAGAAATAATAGATTTATTTTTAT
159493124    ~~~~~~~~~~~~~~~~~~~~~~~~~~~~~~~~~~~~~~~~~~~~~~~~~~~~~~~~~~~~~~~~~~~~~~~~~~~~~~~~~~~~~~~~~~
159493661    ~~~~~~~~~~~~~~~~~~~~~~~~~~~~~~~~~~~~~~~~~~~~~~~~~~~~~~~~~~~~~~~~~~~~~~~~~~~~~~~~~~~~~~~~~~
159494727    ~~~~~~~~~~~~~~~~~~~~~~~~~~~~~~~~~~~~~~~~~~~~~~~~~~~~~~~~~~~~~~~~~~~~~~~~~~~~~~~~~~~~~~~~~~
159496944    ~~~~~~~~~~~~~~~~~~~~~~~~~~~~~~~~~~~~~~~~~~~~~~~~~~~~~~~~~~~~~~~~~~~~~~~~~~~~~~~~~~~~~~~~~~
159494492    ~~~~~~~~~~~~~~~~~~~~~~~~~~~~~~~~~~~~~~~~~~~~~~~~~~~~~~~~~~~~~~~~~~~~~~~~~~~~~~~~~~~~~~~~~~
159496140    ~~~~~~~~~~~~~~~~~~~~~~~~~~~~~~~~~~~~~~~~~~~~~~~~~~~~~~~~~~~~~~~~~~~~~~~~~~~~~~~~~~~~~~~~~~
159497160    ~~~~~~~~~~~~~~~~~~~~~~~~~~~~~~~~~~~~~~~~~~~~~~~~~~~~~~~~~~~~~~~~~~~~~~~~~~~~~~~~~~~~~~~~~~
aaa07c02.g1

                     2530      2540      2550      2560      2570      2580      2590      2600      2610
             ....|....|....|....|....|....|....|....|....|....|....|....|....|....|....|....|....|....|
rsmitogen    gatctactttagtaactgtatttttgttaattttatctttaccagttttggctggaggtattactatgttactttttgatcgaaatttta
rscox2
rstrna-his
rs16s
rsnad3
rstrna-leu
rscox3       gatctactttagtaactgtatttttgttaattttatctttaccagttttggctggaggtattactatgttactttttgatcgaaatttta
rstrna-pro   ~~~~~~~~~~~~~~~~~~~~~~~~~~~~~~~~~~~~~~~~~~~~~~~~~~~~~~~~~~~~~~~~~~~~~~~~~~~~~~~~~~~~~~~~~~
rstrna-arg   ~~~~~~~~~~~~~~~~~~~~~~~~~~~~~~~~~~~~~~~~~~~~~~~~~~~~~~~~~~~~~~~~~~~~~~~~~~~~~~~~~~~~~~~~~~
rsnad6       ~~~~~~~~~~~~~~~~~~~~~~~~~~~~~~~~~~~~~~~~~~~~~~~~~~~~~~~~~~~~~~~~~~~~~~~~~~~~~~~~~~~~~~~~~~
rsnad4L      ~~~~~~~~~~~~~~~~~~~~~~~~~~~~~~~~~~~~~~~~~~~~~~~~~~~~~~~~~~~~~~~~~~~~~~~~~~~~~~~~~~~~~~~~~~
rstrna-val   ~~~~~~~~~~~~~~~~~~~~~~~~~~~~~~~~~~~~~~~~~~~~~~~~~~~~~~~~~~~~~~~~~~~~~~~~~~~~~~~~~~~~~~~~~~
rstrna-leu   ~~~~~~~~~~~~~~~~~~~~~~~~~~~~~~~~~~~~~~~~~~~~~~~~~~~~~~~~~~~~~~~~~~~~~~~~~~~~~~~~~~~~~~~~~~
159494961oke
159496557oke
159497093oke
159496622oke
159498117oke
159494290oke
159495636oke
159497178oke
159498304oke
159496326oke
159495404oke
159493141oke
159493570oke
159434499oke
159493217oke
159493216oke
159493234oke
159492908oke
159491934oke
159492538    GATCTACTTTAGTAACTGTATTTTTGTTAATTTTATCTTTACCAGTTTTGGCTGGAGGTATTACTATGTTACTTTTTGATCGAAATTTTA
159498161    GATCTACTTTAGTAACTGTATTTTTGTTAATTTTATCTTTACCAGTTTTGGCTGGAGGTATTACTATGTTACTTTTTGATCGAAATTTTA
159493124    ~~~~~~~~~~~~~~~~~~~~~~~~~~~~~~~~~~~~~~~~~~~~~~~~~~~~~~~~~~AGGCGGCCGACATGTTCCATAAAGGGGTTTTT
159493661    ~~~~~~~~~~~~~~~~~~~~~~~~~~~~~~~~~~~~~~~~~~~~~~~~~~~~~~~~~~~~~~~~~~~~~~~~~~~~~~~~~~~~~~~~~~
159494727    ~~~~~~~~~~~~~~~~~~~~~~~~~~~~~~~~~~~~~~~~~~~~~~~~~~~~~~~~~~~~~~~~~~~~~~~~~~~~~~~~~~~~~~~~~~
159496944    ~~~~~~~~~~~~~~~~~~~~~~~~~~~~~~~~~~~~~~~~~~~~~~~~~~~~~~~~~~~~~~~~~~~~~~~~~~~~~~~~~~~~~~~~~~
159494492    ~~~~~~~~~~~~~~~~~~~~~~~~~~~~~~~~~~~~~~~~~~~~~~~~~~~~~~~~~~~~~~~~~~~~~~~~~~~~~~~~~~~~~~~~~~
159496140    ~~~~~~~~~~~~~~~~~~~~~~~~~~~~~~~~~~~~~~~~~~~~~~~~~~~~~~~~~~~~~~~~~~~~~~~~~~~~~~~~~~~~~~~~~~
159497160    ~~~~~~~~~~~~~~~~~~~~~~~~~~~~~~~~~~~~~~~~~~~~~~~~~~~~~~~~~~~~~~~~~~~~~~~~~~~~~~~~~~~~~~~~~~
aaa07c02.g1

                     2620      2630      2640      2650      2660      2670      2680      2690      2700
             ....|....|....|....|....|....|....|....|....|....|....|....|....|....|....|....|....|....|
rsmitogen    attctaatttttttgaagtaggaagtggaggtaatagtttaatatttcagcatctttttttgatttttttggacatcctgaagtttaaattt
rscox2
rstrna-his
rs16s
rsnad3
rstrna-leu
rscox3       attctaatttttttgaagtaggaagtggaggtaatagtttaatatttcagcatctttttttgatttttttggacatcctgaagtttaaattt
rstrna-pro   ~~~~~~~~~~~~~~~~~~~~~~~~~~~~~~~~~~~~~~~~~~~~~~~~~~~~~~~~~~~~~~~~~~~~~~~~~~~~~~~~~~~~~~~~~~
rstrna-arg   ~~~~~~~~~~~~~~~~~~~~~~~~~~~~~~~~~~~~~~~~~~~~~~~~~~~~~~~~~~~~~~~~~~~~~~~~~~~~~~~~~~~~~~~~~~
rsnad6       ~~~~~~~~~~~~~~~~~~~~~~~~~~~~~~~~~~~~~~~~~~~~~~~~~~~~~~~~~~~~~~~~~~~~~~~~~~~~~~~~~~~~~~~~~~
rsnad4L      ~~~~~~~~~~~~~~~~~~~~~~~~~~~~~~~~~~~~~~~~~~~~~~~~~~~~~~~~~~~~~~~~~~~~~~~~~~~~~~~~~~~~~~~~~~
rstrna-val   ~~~~~~~~~~~~~~~~~~~~~~~~~~~~~~~~~~~~~~~~~~~~~~~~~~~~~~~~~~~~~~~~~~~~~~~~~~~~~~~~~~~~~~~~~~
rstrna-leu   ~~~~~~~~~~~~~~~~~~~~~~~~~~~~~~~~~~~~~~~~~~~~~~~~~~~~~~~~~~~~~~~~~~~~~~~~~~~~~~~~~~~~~~~~~~
159494961oke
159496557oke
159497093oke
159496622oke
159498117oke
159494290oke
159495636oke
159497178oke
159498304oke
159496326oke
159495404oke
159493141oke
159493570oke
159434499oke
159493217oke
159493216oke
159493234oke
159492908oke
159491934oke
159492538    ATTCTAATTTTTTTGAAGTAGGAAGTGGAGGTAATAGTTTAATATTTCCGCATCTCCCGGCC~
159498161    ATTCTAATTTTTTTGAAGTAGGAAGTGGAGGTAATAGTTTAATATTTCAGCATCTTTTTTGATTTTTTGGACATCCTGAAGTTTAAATTT
159493124    TTTTTTTTTTTTGTGAAGGAGGAAGCTTTGGGGGGAGTTTAATATTTCAGCATCTTTTTTGATTTTTTGGACATCCTGAAGTTGAAATTT
159493661    ~~~~~~~~~~~~~~~~~~~~~~~~~~~~~~~~~~~~~~~~~~~~~~~~~~~~~~~~~~~~~~~~~~~~~~~~~~~~~~~~~~~~~~~~~~
159494727    ~~~~~~~~~~~~~~~~~~~~~~~~~~~~~~~~~~~~~~~~~~~~~~~~~~~~~~~~~~~~~~~~~~~~~~~~~~~~~~~~~~~~~~~~~~
159496944    ~~~~~~~~~~~~~~~~~~~~~~~~~~~~~~~~~~~~~~~~~~~~~~~~~~~~~~~~~~~~~~~~~~~~~~~~~~~~~~~~~~~~~~~~~~
159494492    ~~~~~~~~~~~~~~~~~~~~~~~~~~~~~~~~~~~~~~~~~~~~~~~~~~~~~~~~~~~~~~~~~~~~~~~~~~~~~~~~~~~~~~~~~~
159496140    ~~~~~~~~~~~~~~~~~~~~~~~~~~~~~~~~~~~~~~~~~~~~~~~~~~~~~~~~~~~~~~~~~~~~~~~~~~~~~~~~~~~~~~~~~~
159497160    ~~~~~~~~~~~~~~~~~~~~~~~~~~~~~~~~~~~~~~~~~~~~~~~~~~~~~~~~~~~~~~~~~~~~~~~~~~~~~~~~~~~~~~~~~~
aaa07c02.g1

                     2710      2720      2730      2740      2750      2760      2770      2780      2790
             ....|....|....|....|....|....|....|....|....|....|....|....|....|....|....|....|....|....|
rsmitogen    taattttaccagcttttggtttagtcagacaaacaactttattaatttctggtaaaaaaatagtttttggttcagtagctataatttctg
rscox2
rstrna-his
rs16s
rsnad3
rstrna-leu
rscox3       taattttaccagcttttggtttagtcagacaaacaactttattaatttctggtaaaaaaatagtttttggttcagtagctataatttctg
rstrna-pro   ~~~~~~~~~~~~~~~~~~~~~~~~~~~~~~~~~~~~~~~~~~~~~~~~~~~~~~~~~~~~~~~~~~~~~~~~~~~~~~~~~~~~~~~~~~
rstrna-arg   ~~~~~~~~~~~~~~~~~~~~~~~~~~~~~~~~~~~~~~~~~~~~~~~~~~~~~~~~~~~~~~~~~~~~~~~~~~~~~~~~~~~~~~~~~~
rsnad6       ~~~~~~~~~~~~~~~~~~~~~~~~~~~~~~~~~~~~~~~~~~~~~~~~~~~~~~~~~~~~~~~~~~~~~~~~~~~~~~~~~~~~~~~~~~
rsnad4L      ~~~~~~~~~~~~~~~~~~~~~~~~~~~~~~~~~~~~~~~~~~~~~~~~~~~~~~~~~~~~~~~~~~~~~~~~~~~~~~~~~~~~~~~~~~
rstrna-val   ~~~~~~~~~~~~~~~~~~~~~~~~~~~~~~~~~~~~~~~~~~~~~~~~~~~~~~~~~~~~~~~~~~~~~~~~~~~~~~~~~~~~~~~~~~
rstrna-leu   ~~~~~~~~~~~~~~~~~~~~~~~~~~~~~~~~~~~~~~~~~~~~~~~~~~~~~~~~~~~~~~~~~~~~~~~~~~~~~~~~~~~~~~~~~~
159494961oke
159496557oke
159497093oke
159496622oke
159498117oke
```

```
159494290oke
159495636oke
159497178oke
159498304oke
159496326oke
159495404oke
159493141oke
159493570oke
159493499oke
159493217oke
159493216oke
159493234oke
159492908oke
159491934oke
159492538
159498161      TAATTTTACCAGCTTTTGGTTTAGTCAGACAAACAACTTTATTAATTTCTGGTAAAAAAATAGTTTTTGATTCAGTAGCTATCCCGGCC~
159493124      TAATTTTTCCAGCTTTTGGTTTAGTCAGACAAACAACTTTATTAATTTCTGGTAAAAAAATAGTTTTTGGTTCAGTAGCTATAATTTCTG
159493661      ~~~~~~~~~~~~~~~~~~~~~~~~~~~~~~~~~~~~~~~~~~~~~~~~~~~~~~~~~~~~~~~~~~~~~~~~~~~~~~~~~~~~~~~~~~
159494727      ~~~~~~~~~~~~~~~~~~~~~~~~~~~~~~~~~~~~~~~~~~~~~~~~~~~~~~~~~~~~~~~~~~~~~~~~~~~~~~~~~~~~~~~~~~
159496944      ~~~~~~~~~~~~~~~~~~~~~~~~~~~~~~~~~~~~~~~~~~~~~~~~~~~~~~~~~~~~~~~~~~~~~~~~~~~~~~~~~~~~~~~~~~
159494492      ~~~~~~~~~~~~~~~~~~~~~~~~~~~~~~~~~~~~~~~~~~~~~~~~~~~~~~~~~~~~~~~~~~~~~~~~~~~~~~~~~~~~~~~~~~
159496140      ~~~~~~~~~~~~~~~~~~~~~~~~~~~~~~~~~~~~~~~~~~~~~~~~~~~~~~~~~~~~~~~~~~~~~~~~~~~~~~~~~~~~~~~~~~
159497160      ~~~~~~~~~~~~~~~~~~~~~~~~~~~~~~~~~~~~~~~~~~~~~~~~~~~~~~~~~~~~~~~~~~~~~~~~~~~~~~~~~~~~~~~~~~
aaa07c02.g1

                       2800      2810      2820      2830      2840      2850      2860      2870      2880
               ....|....|....|....|....|....|....|....|....|....|....|....|....|....|....|....|....|....|
rsmitogen      ctataatagtaattggttttattggttgtttagtatgagctcaccatatatttacagtgggattagattttgattcacgtgcttatttta
rscox2
rstrna-his
rs16s
rsnad3
rstrna-leu
rscox3         ctataatagtaattggttttattggttgtttagtatgagctcaccatatatttacagtgggattagattttgattcacgtgcttatttta
rstrna-pro     ~~~~~~~~~~~~~~~~~~~~~~~~~~~~~~~~~~~~~~~~~~~~~~~~~~~~~~~~~~~~~~~~~~~~~~~~~~~~~~~~~~~~~~~~~~
rstrna-arg     ~~~~~~~~~~~~~~~~~~~~~~~~~~~~~~~~~~~~~~~~~~~~~~~~~~~~~~~~~~~~~~~~~~~~~~~~~~~~~~~~~~~~~~~~~~
rsnad6         ~~~~~~~~~~~~~~~~~~~~~~~~~~~~~~~~~~~~~~~~~~~~~~~~~~~~~~~~~~~~~~~~~~~~~~~~~~~~~~~~~~~~~~~~~~
rsnad4L        ~~~~~~~~~~~~~~~~~~~~~~~~~~~~~~~~~~~~~~~~~~~~~~~~~~~~~~~~~~~~~~~~~~~~~~~~~~~~~~~~~~~~~~~~~~
rstrna-val     ~~~~~~~~~~~~~~~~~~~~~~~~~~~~~~~~~~~~~~~~~~~~~~~~~~~~~~~~~~~~~~~~~~~~~~~~~~~~~~~~~~~~~~~~~~
rstrna-leu     ~~~~~~~~~~~~~~~~~~~~~~~~~~~~~~~~~~~~~~~~~~~~~~~~~~~~~~~~~~~~~~~~~~~~~~~~~~~~~~~~~~~~~~~~~~
159494961oke
159496557oke
159497093oke
159496622oke
159498117oke
159494290oke
159495636oke
159497178oke
159498304oke
159496326oke
159495404oke
159493141oke
159493570oke
159493499oke
159493217oke
159493216oke
159493234oke
159492908oke
159491934oke
159492538
159498161
159493124      CTATAATAGTAATTGGTTTTATTGGTTGTTTAGTATGAGCTCACCATATATTTACAGTGGGATTAGATTTTGATTCACGTGCTTATTTTA
159493661      ~~~~~~~~~~~~~~~~~~~~~~~~~~~~~~~~~~~~~~~~~~~~~~~~~~~~~~~~~~~~~~~~~~~~~~~~~~~~~~~~~~~~~~~~~~
159494727      ~~~~~~~~~~~~~~~~~~~~~~~~~~~~~~~~~~~~~~~~~~~~~~~~~~~~~~~~~~~~~~~~~~~~~~~~~~~~~~~~~~~~~~~~~~
159496944      ~~~~~~~~~~~~~~~~~~~~~~~~~~~~~~~~~~~~~~~~~~~~~~~~~~~~~~~~~~~~~~~~~~~~~~~~~~~~~~~~~~~~~~~~~~
159494492      ~~~~~~~~~~~~~~~~~~~~~~~~~~~~~~~~~~~~~~~~~~~~~~~~~~~~~~~~~~~~~~~~~~~~~~~~~~~~~~~~~~~~~~~~~~
159496140      ~~~~~~~~~~~~~~~~~~~~~~~~~~~~~~~~~~~~~~~~~~~~~~~~~~~~~~~~~~~~~~~~~~~~~~~~~~~~~~~~~~~~~~~~~~
159497160      ~~~~~~~~~~~~~~~~~~~~~~~~~~~~~~~~~~~~~~~~~~~~~~~~~~~~~~~~~~~~~~~~~~~~~~~~~~~~~~~~~~~~~~~~~~
aaa07c02.g1

                       2890      2900      2910      2920      2930      2940      2950      2960      2970
               ....|....|....|....|....|....|....|....|....|....|....|....|....|....|....|....|....|....|
rsmitogen      ctgctgctacaatagttattgctgttcctacaggtattaaagtttttagatgaataataactttatatggttcttaaataattaataata
rscox2
rstrna-his
rs16s
rsnad3
rstrna-leu
rscox3         ctgctgctacaatagttattgctgttcctacaggtattaaagtttttagatgaataataactttatatggttcttaaataattaataata
rstrna-pro     ~~~~~~~~~~~~~~~~~~~~~~~~~~~~~~~~~~~~~~~~~~~~~~~~~~~~~~~~~~~~~~~~~~~~~~~~~~~~~~~~~~~~~~~~~~
rstrna-arg     ~~~~~~~~~~~~~~~~~~~~~~~~~~~~~~~~~~~~~~~~~~~~~~~~~~~~~~~~~~~~~~~~~~~~~~~~~~~~~~~~~~~~~~~~~~
rsnad6         ~~~~~~~~~~~~~~~~~~~~~~~~~~~~~~~~~~~~~~~~~~~~~~~~~~~~~~~~~~~~~~~~~~~~~~~~~~~~~~~~~~~~~~~~~~
rsnad4L        ~~~~~~~~~~~~~~~~~~~~~~~~~~~~~~~~~~~~~~~~~~~~~~~~~~~~~~~~~~~~~~~~~~~~~~~~~~~~~~~~~~~~~~~~~~
rstrna-val     ~~~~~~~~~~~~~~~~~~~~~~~~~~~~~~~~~~~~~~~~~~~~~~~~~~~~~~~~~~~~~~~~~~~~~~~~~~~~~~~~~~~~~~~~~~
rstrna-leu     ~~~~~~~~~~~~~~~~~~~~~~~~~~~~~~~~~~~~~~~~~~~~~~~~~~~~~~~~~~~~~~~~~~~~~~~~~~~~~~~~~~~~~~~~~~
159494961oke
159496557oke
159497093oke
159496622oke
159498117oke
159494290oke
```

```
159495636oke
159497178oke
159498304oke
159496326oke
159495404oke
159493141oke
159493570oke
159493499oke
159493217oke
159493216oke
159493234oke
159492908oke
159491934oke
159492538
159498161
159493124      CTGCTGCTACAATAGTTATTGCTGTTCCTACAGGTATTAAAGTTTTTAGATGAATAATAACTTTATATGGTTCTTAAATAATTAATAATA
159493661      ~~~~~~~~~~~~~~~~~~~~~~~~~~~~~~~~GGTATTAAAGTTTTTAGATGAATAATAACTTTATATGGTTCTTAAATAATTAATAATA
159494727      ~~~~~~~~~~~~~~~~~~~~~~~~~~~~~~~~~~~~~~~~~~~~~~~~~~~~~~~~~~~~~~~~~~~~~~~~~~~~~~~~~~~~~~~~~~
159496944      ~~~~~~~~~~~~~~~~~~~~~~~~~~~~~~~~~~~~~~~~~~~~~~~~~~~~~~~~~~~~~~~~~~~~~~~~~~~~~~~~~~~~~~~~~~
159494492      ~~~~~~~~~~~~~~~~~~~~~~~~~~~~~~~~~~~~~~~~~~~~~~~~~~~~~~~~~~~~~~~~~~~~~~~~~~~~~~~~~~~~~~~~~~
159496140      ~~~~~~~~~~~~~~~~~~~~~~~~~~~~~~~~~~~~~~~~~~~~~~~~~~~~~~~~~~~~~~~~~~~~~~~~~~~~~~~~~~~~~~~~~~
159497160      ~~~~~~~~~~~~~~~~~~~~~~~~~~~~~~~~~~~~~~~~~~~~~~~~~~~~~~~~~~~~~~~~~~~~~~~~~~~~~~~~~~~~~~~~~~
aaa07c02.g1

                       2980      2990      3000      3010      3020      3030      3040      3050      3060
               ....|....|....|....|....|....|....|....|....|....|....|....|....|....|....|....|....|....|
rsmitogen      taatagtagaatgattataaggttttatttttttatttactttaggtggtttaactggtttagtattatctaattctagtttagatattc
rscox2
rstrna-his
rs16s
rsnad3
rstrna-leu
rscox3         taatagtagaatgattataaggttttatttttttatttactttaggtggtttaactggtttagtattatctaattctagtttagatattc
rstrna-pro     ~~~~~~~~~~~~~~~~~~~~~~~~~~~~~~~~~~~~~~~~~~~~~~~~~~~~~~~~~~~~~~~~~~~~~~~~~~~~~~~~~~~~~~~~~~
rstrna-arg     ~~~~~~~~~~~~~~~~~~~~~~~~~~~~~~~~~~~~~~~~~~~~~~~~~~~~~~~~~~~~~~~~~~~~~~~~~~~~~~~~~~~~~~~~~~
rsnad6         ~~~~~~~~~~~~~~~~~~~~~~~~~~~~~~~~~~~~~~~~~~~~~~~~~~~~~~~~~~~~~~~~~~~~~~~~~~~~~~~~~~~~~~~~~~
rsnad4L        ~~~~~~~~~~~~~~~~~~~~~~~~~~~~~~~~~~~~~~~~~~~~~~~~~~~~~~~~~~~~~~~~~~~~~~~~~~~~~~~~~~~~~~~~~~
rstrna-val     ~~~~~~~~~~~~~~~~~~~~~~~~~~~~~~~~~~~~~~~~~~~~~~~~~~~~~~~~~~~~~~~~~~~~~~~~~~~~~~~~~~~~~~~~~~
rstrna-leu     ~~~~~~~~~~~~~~~~~~~~~~~~~~~~~~~~~~~~~~~~~~~~~~~~~~~~~~~~~~~~~~~~~~~~~~~~~~~~~~~~~~~~~~~~~~
159494961oke
159496557oke
159497093oke
159496622oke
159498117oke
159494290oke
159495636oke
159497178oke
159498304oke
159496326oke
159495404oke
159493141oke
159493570oke
159493499oke
159493217oke
159493216oke
159493234oke
159492908oke
159491934oke
159492538
159498161
159493124      TAATAGTAGAATGATTATAAGGTTTTATTTTTTTATTTACTTTAGGTGGTTTAACTGGTTTAGTATTATCTAATTCTAGTTTAGATATTC
159493661      TAATAGTAGAATGATTATAAGGTTTTATTTTTTTATTTACTTTAGGTGGTTTAACTGGTTTAGTATTATCTAATTCTAGTTTAGATATTC
159494727      ~~~~~~~~~~~~~~~~~~~~~~~~~~~~~~~~~~~~~~~~~~~~~~~~~~~~~~~~~~~~~~~~~~~~~~~~~~~~~~~~~~~~~~~~~~
159496944      ~~~~~~~~~~~~~~~~~~~~~~~~~~~~~~~~~~~~~~~~~~~~~~~~~~~~~~~~~~~~~~~~~~~~~~~~~~~~~~~~~~~~~~~~~~
159494492      ~~~~~~~~~~~~~~~~~~~~~~~~~~~~~~~~~~~~~~~~~~~~~~~~~~~~~~~~~~~~~~~~~~~~~~~~~~~~~~~~~~~~~~~~~~
159496140      ~~~~~~~~~~~~~~~~~~~~~~~~~~~~~~~~~~~~~~~~~~~~~~~~~~~~~~~~~~~~~~~~~~~~~~~~~~~~~~~~~~~~~~~~~~
159497160      ~~~~~~~~~~~~~~~~~~~~~~~~~~~~~~~~~~~~~~~~~~~~~~~~~~~~~~~~~~~~~~~~~~~~~~~~~~~~~~~~~~~~~~~~~~
aaa07c02.g1

                       3070      3080      3090      3100      3110      3120      3130      3140      3150
               ....|....|....|....|....|....|....|....|....|....|....|....|....|....|....|....|....|....|
rsmitogen      tattacatgatacttattatgtcgtagctcattttcattaagttttaagtttaggtgcagtttttggtattattattggtttttttgttt
rscox2
rstrna-his
rs16s
rsnad3
rstrna-leu
rscox3         tattacatgatacttattatgtcgtagctcattttcattaagttttaagtttaggtgcagtttttggtattattattggtttttttgttt
rstrna-pro     ~~~~~~~~~~~~~~~~~~~~~~~~~~~~~~~~~~~~~~~~~~~~~~~~~~~~~~~~~~~~~~~~~~~~~~~~~~~~~~~~~~~~~~~~~~
rstrna-arg     ~~~~~~~~~~~~~~~~~~~~~~~~~~~~~~~~~~~~~~~~~~~~~~~~~~~~~~~~~~~~~~~~~~~~~~~~~~~~~~~~~~~~~~~~~~
rsnad6         ~~~~~~~~~~~~~~~~~~~~~~~~~~~~~~~~~~~~~~~~~~~~~~~~~~~~~~~~~~~~~~~~~~~~~~~~~~~~~~~~~~~~~~~~~~
rsnad4L        ~~~~~~~~~~~~~~~~~~~~~~~~~~~~~~~~~~~~~~~~~~~~~~~~~~~~~~~~~~~~~~~~~~~~~~~~~~~~~~~~~~~~~~~~~~
rstrna-val     ~~~~~~~~~~~~~~~~~~~~~~~~~~~~~~~~~~~~~~~~~~~~~~~~~~~~~~~~~~~~~~~~~~~~~~~~~~~~~~~~~~~~~~~~~~
rstrna-leu     ~~~~~~~~~~~~~~~~~~~~~~~~~~~~~~~~~~~~~~~~~~~~~~~~~~~~~~~~~~~~~~~~~~~~~~~~~~~~~~~~~~~~~~~~~~
159494961oke
159496557oke
159497093oke
159496622oke
159498117oke
159494290oke
159495636oke
```

```
159497178oke
159498304oke
159496326oke
159495404oke
159493141oke
159493570oke
159493499oke
159493217oke
159493216oke
159493234oke
159492908oke
159491934oke
159492538
159498161
159493124    TATTACATGATACTTATTATGTCGTAGCTCATTTTCATTAAGCCCGGCCGTA~
159493661    TATTACATGATACTTATTATGTCGTAGCTCATTTTCATTAAGTTTTAAGTTTAGGTGCAGTTTTTGGTATTATTATTGGTTTTTTTGTTT
159494727    ~~~~~~~~~~~~~~~~~~~~~~~~~~~~~~~~~~~~~~~~~~~~~~~~~~~~~~~~~~~~~~~~~~~~~~~~~~~~~~~~~~~~~~~~~~
159496944    ~~~~~~~~~~~~~~~~~~~~~~~~~~~~~~~~~~~~~~~~~~~~~~~~~~~~~~~~~~~~~~~~~~~~~~~~~~~~~~~~~~~~~~~~~~
159494492    ~~~~~~~~~~~~~~~~~~~~~~~~~~~~~~~~~~~~~~~~~~~~~~~~~~~~~~~~~~~~~~~~~~~~~~~~~~~~~~~~~~~~~~~~~~
159496140    ~~~~~~~~~~~~~~~~~~~~~~~~~~~~~~~~~~~~~~~~~~~~~~~~~~~~~~~~~~~~~~~~~~~~~~~~~~~~~~~~~~~~~~~~~~
159497160    ~~~~~~~~~~~~~~~~~~~~~~~~~~~~~~~~~~~~~~~~~~~~~~~~~~~~~~~~~~~~~~~~~~~~~~~~~~~~~~~~~~~~~~~~~~
aaa07c02.g1

                     3160      3170      3180      3190      3200      3210      3220      3230      3240
             ....|....|....|....|....|....|....|....|....|....|....|....|....|....|....|....|....|....|
rsmitogen    attaaggttaattttaaggtttagttttaaataaaattttagttaattcattttataaaatgatttttttaggtgttaatataactttttt
rscox2
rstrna-his
rs16s
rsnad3
rstrna-leu
rscox3       attaaggttaattttaaggtttagttttaaataaaattttagttaattcattttataaaatgatttttttaggtgttaatataactttttt
rstrna-pro   ~~~~~~~~~~~~~~~~~~~~~~~~~~~~~~~~~~~~~~~~~~~~~~~~~~~~~~~~~~~~~~~~~~~~~~~~~~~~~~~~~~~~~~~~~~
rstrna-arg   ~~~~~~~~~~~~~~~~~~~~~~~~~~~~~~~~~~~~~~~~~~~~~~~~~~~~~~~~~~~~~~~~~~~~~~~~~~~~~~~~~~~~~~~~~~
rsnad6       ~~~~~~~~~~~~~~~~~~~~~~~~~~~~~~~~~~~~~~~~~~~~~~~~~~~~~~~~~~~~~~~~~~~~~~~~~~~~~~~~~~~~~~~~~~
rsnad4L      ~~~~~~~~~~~~~~~~~~~~~~~~~~~~~~~~~~~~~~~~~~~~~~~~~~~~~~~~~~~~~~~~~~~~~~~~~~~~~~~~~~~~~~~~~~
rstrna-val   ~~~~~~~~~~~~~~~~~~~~~~~~~~~~~~~~~~~~~~~~~~~~~~~~~~~~~~~~~~~~~~~~~~~~~~~~~~~~~~~~~~~~~~~~~~
rstrna-leu   ~~~~~~~~~~~~~~~~~~~~~~~~~~~~~~~~~~~~~~~~~~~~~~~~~~~~~~~~~~~~~~~~~~~~~~~~~~~~~~~~~~~~~~~~~~
159494961oke
159496557oke
159497093oke
159496622oke
159498117oke
159494290oke
159495636oke
159497178oke
159498304oke
159496326oke
159495404oke
159493141oke
159493570oke
159493499oke
159493217oke
159493216oke
159493234oke
159492908oke
159491934oke
159492538
159498161
159493124
159493661    ATTAAGGTTAATTTTAAGGTTTAGTTTTAAATAAAATTTTAGTTAATTCATTTTATAAAATGATTTTTTTAGGTGTTAATATAACTTTTTT
159494727    ~~~~~~~~~~~~~~~~~~~~~~~~~~~~~~~~~~~~~~~~~~~~~~~~~~~~~~~~~~~~~~~~~~~~~~~~~~~~~~~~~~~~~~~~~~
159496944    ~~~~~~~~~~~~~~~~~~~~~~~~~~~~~~~~~~~~~~~~~~~~~~~~~~~~~~~~~~~~~~~~~~~~~~~~~~~~~~~~~~~~~~~~~~
159494492    ~~~~~~~~~~~~~~~~~~~~~~~~~~~~~~~~~~~~~~~~~~~~~~~~~~~~~~~~~~~~~~~~~~~~~~~~~~~~~~~~~~~~~~~~~~
159496140    ~~~~~~~~~~~~~~~~~~~~~~~~~~~~~~~~~~~~~~~~~~~~~~~~~~~~~~~~~~~~~~~~~~~~~~~~~~~~~~~~~~~~~~~~~~
159497160    ~~~~~~~~~~~~~~~~~~~~~~~~~~~~~~~~~~~~~~~~~~~~~~~~~~~~~~~~~~~~~~~~~~~~~~~~~~~~~~~~~~~~~~~~~~
aaa07c02.g1

                     3250      3260      3270      3280      3290      3300      3310      3320      3330
             ....|....|....|....|....|....|....|....|....|....|....|....|....|....|....|....|....|....|
rsmitogen    ttccattacattttgctggtttacaaggatttccacgtaagtaagttgattaaattgatatttaaagtatttgaaatattattagttctt
rscox2
rstrna-his
rs16s
rsnad3
rstrna-leu
rscox3       ttccattacattttgctggtttacaaggatttccacgtaagtaagttgattaaattgatatttaaagtatttgaaatattattagttctt
rstrna-pro   ~~~~~~~~~~~~~~~~~~~~~~~~~~~~~~~~~~~~~~~~~~~~~~~~~~~~~~~~~~~~~~~~~~~~~~~~~~~~~~~~~~~~~~~~~~
rstrna-arg   ~~~~~~~~~~~~~~~~~~~~~~~~~~~~~~~~~~~~~~~~~~~~~~~~~~~~~~~~~~~~~~~~~~~~~~~~~~~~~~~~~~~~~~~~~~
rsnad6       ~~~~~~~~~~~~~~~~~~~~~~~~~~~~~~~~~~~~~~~~~~~~~~~~~~~~~~~~~~~~~~~~~~~~~~~~~~~~~~~~~~~~~~~~~~
rsnad4L      ~~~~~~~~~~~~~~~~~~~~~~~~~~~~~~~~~~~~~~~~~~~~~~~~~~~~~~~~~~~~~~~~~~~~~~~~~~~~~~~~~~~~~~~~~~
rstrna-val   ~~~~~~~~~~~~~~~~~~~~~~~~~~~~~~~~~~~~~~~~~~~~~~~~~~~~~~~~~~~~~~~~~~~~~~~~~~~~~~~~~~~~~~~~~~
rstrna-leu   ~~~~~~~~~~~~~~~~~~~~~~~~~~~~~~~~~~~~~~~~~~~~~~~~~~~~~~~~~~~~~~~~~~~~~~~~~~~~~~~~~~~~~~~~~~
159494961oke
159496557oke
159497093oke
159496622oke
159498117oke
159494290oke
159495636oke
159497178oke
159498304oke
159496326oke
159495404oke
159493141oke
159493570oke
159493499oke
159493217oke
159493216oke
159493234oke
159492908oke
159491934oke
159492538
159498161
159493124
159493661    TTCCATTACATTTTGCCGGTTTACAAGGATTTCCACGTAAGTAAGTTGATTAAATTGATATTTAAAGTATTTGAAATATTATTAGTTCTT
159494727    ~~~~~~~~~~~~~~~~~~~~~~~~~~~~~~~~~~~~~~~~~~~~~~~~~~~~~~~~~~~~~~~~~~~~~~~~~~~~~~~~~~~~~~~~~~
159496944    ~~~~~~~~~~~~~~~~~~~~~~~~~~~~~~~~~~~~~~~~~~~~~~~~~~~~~~~~~~~~~~~~~~~~~~~~~~~~~~~~~~~~~~~~~~
159494492    ~~~~~~~~~~~~~~~~~~~~~~~~~~~~~~~~~~~~~~~~~~~~~~~~~~~~~~~~~~~~~~~~~~~~~~~~~~~~~~~~~~~~~~~~~~
159496140    ~~~~~~~~~~~~~~~~~~~~~~~~~~~~~~~~~~~~~~~~~~~~~~~~~~~~~~~~~~~~~~~~~~~~~~~~~~~~~~~~~~~~~~~~~~
159497160    ~~~~~~~~~~~~~~~~~~~~~~~~~~~~~~~~~~~~~~~~~~~~~~~~~~~~~~~~~~~~~~~~~~~~~~~~~~~~~~~~~~~~~~~~~~
aaa07c02.g1

                     3340      3350      3360      3370      3380      3390      3400      3410      3420
             ....|....|....|....|....|....|....|....|....|....|....|....|....|....|....|....|....|....|
rsmitogen    ttggttcaattttgagtacataatcaatatttttttttatttatttattttaagagtctttttttaattttcgtttagtttatttgaatt
rscox2
rstrna-his
rs16s
rsnad3
rstrna-leu
rscox3       ttggttcaattttgagtacataatcaatatttttttttatttatttattttaagagtctttttttaattttcgtttagtttatttgaatt
rstrna-pro   ~~~~~~~~~~~~~~~~~~~~~~~~~~~~~~~~~~~~~~~~~~~~~~~~~~~~~~~~~~~~~~~~~~~~~~~~~~~~~~~~~~~~~~~~~~
rstrna-arg   ~~~~~~~~~~~~~~~~~~~~~~~~~~~~~~~~~~~~~~~~~~~~~~~~~~~~~~~~~~~~~~~~~~~~~~~~~~~~~~~~~~~~~~~~~~
rsnad6       ~~~~~~~~~~~~~~~~~~~~~~~~~~~~~~~~~~~~~~~~~~~~~~~~~~~~~~~~~~~~~~~~~~~~~~~~~~~~~~~~~~~~~~~~~~
rsnad4L      ~~~~~~~~~~~~~~~~~~~~~~~~~~~~~~~~~~~~~~~~~~~~~~~~~~~~~~~~~~~~~~~~~~~~~~~~~~~~~~~~~~~~~~~~~~
rstrna-val   ~~~~~~~~~~~~~~~~~~~~~~~~~~~~~~~~~~~~~~~~~~~~~~~~~~~~~~~~~~~~~~~~~~~~~~~~~~~~~~~~~~~~~~~~~~
rstrna-leu   ~~~~~~~~~~~~~~~~~~~~~~~~~~~~~~~~~~~~~~~~~~~~~~~~~~~~~~~~~~~~~~~~~~~~~~~~~~~~~~~~~~~~~~~~~~
159494961oke
159496557oke
159497093oke
159496622oke
159498117oke
159494290oke
159495636oke
159497178oke
159498304oke
159496326oke
159495404oke
159493141oke
159493570oke
159493499oke
159493217oke
159493216oke
159493234oke
159492908oke
159491934oke
159492538
159498161
159493124
159493661    TTGGTTCAATTTTGAGTACATAATCAATATTTTTTTTTATTTATTTATTTTAAGAGTCTTTTTTTAATTTTCGTTTAGTTTATTTGAATT
159494727    ~TGGTTCAATTTTGAGTACATAATCAATATTTTTTTTTATTTATTTATTTTAAGAGTCTTTTTTAAATTTTCGTTTAGTTTATTTGAATT
159496944    ~~~~~~~~~~~~~~~~~~~~~~~~~~~~~~~~~~~~~~~~~~~~~~~~~~~~~~~~~~~~~~~~~~~~~~~~~~~~~~~~~~~~~~~~~~
159494492    ~~~~~~~~~~~~~~~~~~~~~~~~~~~~~~~~~~~~~~~~~~~~~~~~~~~~~~~~~~~~~~~~~~~~~~~~~~~~~~~~~~~~~~~~~~
159496140    ~~~~~~~~~~~~~~~~~~~~~~~~~~~~~~~~~~~~~~~~~~~~~~~~~~~~~~~~~~~~~~~~~~~~~~~~~~~~~~~~~~~~~~~~~~
159497160    ~~~~~~~~~~~~~~~~~~~~~~~~~~~~~~~~~~~~~~~~~~~~~~~~~~~~~~~~~~~~~~~~~~~~~~~~~~~~~~~~~~~~~~~~~~
aaa07c02.g1

                     3430      3440      3450      3460      3470      3480      3490      3500      3510
             ....|....|....|....|....|....|....|....|....|....|....|....|....|....|....|....|....|....|
rsmitogen    tttttgaaagaaattatttattattagttaaaaatatatatttacatagttttttaagattctttagtttaatattataattgatttattt
rscox2
rstrna-his
rs16s
rsnad3
rstrna-leu
rscox3       tttttgaaagaaattatttattattagttaaaaatatatatttacatagttttttaagattctttagtttaatattataat~
rstrna-pro   ~~~~~~~~~~~~~~~~~~~~~~~~~~~~~~~~~~~~~~~~~~~~~~~~~~~~~~~~~~~~~~~~~~~~~~~~~~~~~~~~~~ tttattt
rstrna-arg   ~~~~~~~~~~~~~~~~~~~~~~~~~~~~~~~~~~~~~~~~~~~~~~~~~~~~~~~~~~~~~~~~~~~~~~~~~~~~~~~~~~~~~~~~~~
rsnad6       ~~~~~~~~~~~~~~~~~~~~~~~~~~~~~~~~~~~~~~~~~~~~~~~~~~~~~~~~~~~~~~~~~~~~~~~~~~~~~~~~~~~~~~~~~~
rsnad4L      ~~~~~~~~~~~~~~~~~~~~~~~~~~~~~~~~~~~~~~~~~~~~~~~~~~~~~~~~~~~~~~~~~~~~~~~~~~~~~~~~~~~~~~~~~~
rstrna-val   ~~~~~~~~~~~~~~~~~~~~~~~~~~~~~~~~~~~~~~~~~~~~~~~~~~~~~~~~~~~~~~~~~~~~~~~~~~~~~~~~~~~~~~~~~~
rstrna-leu   ~~~~~~~~~~~~~~~~~~~~~~~~~~~~~~~~~~~~~~~~~~~~~~~~~~~~~~~~~~~~~~~~~~~~~~~~~~~~~~~~~~~~~~~~~~
159494961oke
159496557oke
159497093oke
159496622oke
159498117oke
159494290oke
159495636oke
159497178oke
159498304oke
```

```
159496326oke
159495404oke
159493141oke
159493570oke
159493499oke
159493217oke
159493216oke
159493234oke
159492908oke
159491934oke
159492538
159498161
159493124
159493661      TTTTTGAAAGAAATTATTTATTATTAGTTAAAAATATATATTTACATAGTTTTTAAGATTCTTTAGTTTAATATCCCGGCC~
159494727      TTTTTGAAAGAAATTATTTATTATTAGTTAAAAATATATATTTACATAGTTTTTAAGATTCTTTAGTTTAATATTATAATTGATTTATTT
159496944      ~~~~~~~~~~~~~~~~~~~~~~~~~~~~~~~~~~~~~~~~~~~~~~~~~~~~~~~~~~~~~~~~~~~~~~~~~~~~~~~TTGA~TTATTT
159494492      ~~~~~~~~~~~~~~~~~~~~~~~~~~~~~~~~~~~~~~~~~~~~~~~~~~~~~~~~~~~~~~~~~~~~~~~~~~~~~~~~~~~~~~~~~~
159496140      ~~~~~~~~~~~~~~~~~~~~~~~~~~~~~~~~~~~~~~~~~~~~~~~~~~~~~~~~~~~~~~~~~~~~~~~~~~~~~~~~~~~~~~~~~~
159497160      ~~~~~~~~~~~~~~~~~~~~~~~~~~~~~~~~~~~~~~~~~~~~~~~~~~~~~~~~~~~~~~~~~~~~~~~~~~~~~~~~~~~~~~~~~~
aaa07c02.g1

                     3520      3530      3540      3550      3560      3570      3580      3590      3600
               ....|....|....|....|....|....|....|....|....|....|....|....|....|....|....|....|....|....|
rsmitogen      tagttaaaatttttcttttgggtagaaaagaattttatcaattttaattcttaattttttttaaatatttattttcgaagtaaaagaatt
rscox2
rstrna-his
rs16s
rsnad3
rstrna-leu
rscox3
rstrna-pro     tagttaaaatttttcttttgggtagaaaagaattttatcaattttaattc~~~~~~~~~~~~~~~~~~~~~~~~~~~~~~~
rstrna-arg     ~~~~~~~~~~~~~~~~~~~~~~~~~~~~~~~~~~~~~~~~~~~~taattcttaattttttttaaatatttattttcgaagtaaaagaatt
rsnad6         ~~~~~~~~~~~~~~~~~~~~~~~~~~~~~~~~~~~~~~~~~~~~~~~~~~~~~~~~~~~~~~~~~~~~~~~~~~~~~~~~~~~~~~~~~~
rsnad4L        ~~~~~~~~~~~~~~~~~~~~~~~~~~~~~~~~~~~~~~~~~~~~~~~~~~~~~~~~~~~~~~~~~~~~~~~~~~~~~~~~~~~~~~~~~~
rstrna-val     ~~~~~~~~~~~~~~~~~~~~~~~~~~~~~~~~~~~~~~~~~~~~~~~~~~~~~~~~~~~~~~~~~~~~~~~~~~~~~~~~~~~~~~~~~~
rstrna-leu     ~~~~~~~~~~~~~~~~~~~~~~~~~~~~~~~~~~~~~~~~~~~~~~~~~~~~~~~~~~~~~~~~~~~~~~~~~~~~~~~~~~~~~~~~~~
159494961oke
159496557oke
159497093oke
159496622oke
159498117oke
159494290oke
159495636oke
159497178oke
159498304oke
159496326oke
159495404oke
159493141oke
159493570oke
159493499oke
159493217oke
159493216oke
159493234oke
159492908oke
159491934oke
159492538
159498161
159493124
159493661
159494727      TAGTTAAAATTTTTCTTTTGGGTAGAAAAGAATTTTATCAATTTTAATTCTTAATTTTTTTTAAATATTTATTTTCGAAGTAAAAGAATT
159496944      TAGTTAAAATTTTTCTTTT~GGTAGAAAAGAATTATATCAATTTTAATTCTTAATTTTTTTTAAATATTTATTTTCGAAGTAAAAGAATT
159494492      ~~~~~~~~~~~~~~~~~~~~~~~~~~~~~~~~~~~~~~~~~~~~~~~~~~~~~~~~~~~~~~~~~~~~~~~~~~~~~~~~~~~~~~~~~~
159496140      ~~~~~~~~~~~~~~~~~~~~~~~~~~~~~~~~~~~~~~~~~~~~~~~~~~~~~~~~~~~~~~~~~~~~~~~~~~~~~~~~~~~~~~~~~~
159497160      ~~~~~~~~~~~~~~~~~~~~~~~~~~~~~~~~~~~~~~~~~~~~~~~~~~~~~~~~~~~~~~~~~~~~~~~~~~~~~~~~~~~~~~~~~~
aaa07c02.g1

                     3610      3620      3630      3640      3650      3660      3670      3680      3690
               ....|....|....|....|....|....|....|....|....|....|....|....|....|....|....|....|....|....|
rsmitogen      atagatttttttttattattttcttatttatttttattattaagttttttagtttaagatattttaaaatgtatgcttttttt~agtatt
rscox2
rstrna-his
rs16s
rsnad3
rstrna-leu
rscox3
rstrna-pro
rstrna-arg     atagatttt~~~~~~~~~~~~
rsnad6         atagatttttttttattattttcttatttatttttattattaagttttttagtttaagatattttaaaatgtatgcttttttt~agtatt
rsnad4L        ~~~~~~~~~~~~~~~~~~~~~~~~~~~~~~~~~~~~~~~~~~~~~~~~~~~~~~~~~~~~~~~~~~~~~~~~~~~~~~~~~~~~~~~~~~
rstrna-val     ~~~~~~~~~~~~~~~~~~~~~~~~~~~~~~~~~~~~~~~~~~~~~~~~~~~~~~~~~~~~~~~~~~~~~~~~~~~~~~~~~~~~~~~~~~
rstrna-leu     ~~~~~~~~~~~~~~~~~~~~~~~~~~~~~~~~~~~~~~~~~~~~~~~~~~~~~~~~~~~~~~~~~~~~~~~~~~~~~~~~~~~~~~~~~~
159494961oke
159496557oke
159497093oke
159496622oke
159498117oke
159494290oke
159495636oke
159497178oke
159498304oke
159496326oke
159495404oke
159493141oke
159493570oke
159493499oke
159493217oke
159493216oke
159493234oke
159492908oke
159491934oke
159492538
159498161
159493124
159493661
159494727      ATAGATTTTTTTTTATTATTTTCTTATTTATTTTTATTATTAAGTTTTTTAGTTTAAGATATTTTAAAATGTATGCTTTTTTT~AGTATT
159496944      ATAGATTTTTTTTTATTATTATCTTATTTATTTTTATTATTAAGTTTTTTAGTTTAAGATATTTTAAAATGTATGCTTTTTTTTAGTATT
159494492      ~~~~~~~~~~~~~~~~~~~~~~~~~~~~~~~~~~~~~~~~~~~~~~~~~~~~~~~~~~~~~~~~~~~~~~~~~~~~~~~~~~~~~~~~~~
159496140      ~~~~~~~~~~~~~~~~~~~~~~~~~~~~~~~~~~~~~~~~~~~~~~~~~~~~~~~~~~~~~~~~~~~~~~~~~~~~~~~~~~~~~~~~~~
159497160
aaa07c02.g1

                     3700      3710      3720      3730      3740      3750      3760      3770      3780
               ....|....|....|....|....|....|....|....|....|....|....|....|....|....|....|....|....|....|
rsmitogen      aagatttataatttttataatttaaattataaaaaattttagtttataataattatatttatttataataatttttgtatcggggatttt
rscox2
rstrna-his
rs16s
rsnad3
rstrna-leu
rscox3
rstrna-pro
rstrna-arg
rsnad6         aagatttataatttttataatttaaattataaaaaattttagtttataataattatatttatttataataatttttgtatcggggatttt
rsnad4L        ~~~~~~~~~~~~~~~~~~~~~~~~~~~~~~~~~~~~~~~~~~~~~~~~~~~~~~~~~~~~~~~~~~~~~~~~~~~~~~~~~~~~~~~~~~
rstrna-val     ~~~~~~~~~~~~~~~~~~~~~~~~~~~~~~~~~~~~~~~~~~~~~~~~~~~~~~~~~~~~~~~~~~~~~~~~~~~~~~~~~~~~~~~~~~
rstrna-leu     ~~~~~~~~~~~~~~~~~~~~~~~~~~~~~~~~~~~~~~~~~~~~~~~~~~~~~~~~~~~~~~~~~~~~~~~~~~~~~~~~~~~~~~~~~~
159494961oke
159496557oke
159497093oke
159496622oke
159498117oke
159494290oke
159495636oke
159497178oke
159498304oke
159496326oke
159495404oke
159493141oke
159493570oke
159493499oke
159493217oke
159493216oke
159493234oke
159492908oke
159491934oke
159492538
159498161
159493124
159493661
159494727      AAGATTTATAATTTTTATAACTTAAATTATAAAAAATTTTAGTTTATAATAATTATATTTCCCGGCC
159496944      AAGATTTATAATTTTTATAATTTAAATTATAAAAAATTTTAGTTTATAATAATTATATTTATTTATAATAATTTTTGTATCGGGGATTTT
159494492      ~~~~~~~~~~~~~~~~~~~~~~~~~~~~~~~~~~~~~~~~~~~~~~~~~~~~~~~~~~~~~~~~~~~~~~~~~~~~~~~~~~~~~~~~~~
159496140      ~~~~~~~~~~~~~~~~~~~~~~~~~~~~~~~~~~~~~~~~~~~~~~~~~~~~~~~~~~~~~~~~~~~~~~~~~~~~~~~~~~~~~~~~~~
159497160      ~~~~~~~~~~~~~~~~~~~~~~~~~~~~~~~~~~~~~~~~~~~~~~~~~~~~~~~~~~~~~~~~~~~~~~~~~~~~~~~~~~~~~~~~~~
aaa07c02.g1

                     3790      3800      3810      3820      3830      3840      3850      3860      3870
               ....|....|....|....|....|....|....|....|....|....|....|....|....|....|....|....|....|....|
rsmitogen      tttattaattatttaacttattactttagtaaaaagttttataataaaaaattattaaatactatttattcctattatttttattaatta
rscox2
rstrna-his
rs16s
rsnad3
rstrna-leu
rscox3
rstrna-pro
rstrna-arg
rsnad6         tttattaattatttaacttattactttagtaaaaagttttataataaaaaattattaaatactatttattcctattatttttattaatta
rsnad4L        ~~~~~~~~~~~~~~~~~~~~~~~~~~~~~~~~~~~~~~~~~~~~~~~~~~~~~~~~~~~~~~~~~~~~~~~~~~~~~~~~~~~~~~~~~~
rstrna-val     ~~~~~~~~~~~~~~~~~~~~~~~~~~~~~~~~~~~~~~~~~~~~~~~~~~~~~~~~~~~~~~~~~~~~~~~~~~~~~~~~~~~~~~~~~~
rstrna-leu     ~~~~~~~~~~~~~~~~~~~~~~~~~~~~~~~~~~~~~~~~~~~~~~~~~~~~~~~~~~~~~~~~~~~~~~~~~~~~~~~~~~~~~~~~~~
159494961oke
159496557oke
159497093oke
159496622oke
159498117oke
159494290oke
159495636oke
159497178oke
159498304oke
159496326oke
159495404oke
```

```
159493141oke
159493570oke
159493499oke
159493217oke
159493216oke
159493234oke
159492908oke
159491934oke
159492538
159498161
159493124
159493661
159494727
159496944      TTTATTAATTATTTAACTTATTACTTTAGTAAAAAGTTTTATAATAAAAAATTATTAAATACTATTTATTCCTATTATTTTTATTAATTA
159494492      ~~~~~~~~~~~~~~~~~~~~~~~~~~~~~~~~~~~~~~~~~~~~~~~~~~~~~~~~~~~~~~~~~~~~~~~~~~~~~~~~~~~~~~~~~~
159496140      ~~~~~~~~~~~~~~~~~~~~~~~~~~~~~~~~~~~~~~~~~~~~~~~~~~~~~~~~~~~~~~~~~~~~~~~~~~~~~~~~~~~~~~~~~~
159497160      ~~~~~~~~~~~~~~~~~~~~~~~~~~~~~~~~~~~~~~~~~~~~~~~~~~~~~~~~~~~~~~~~~~~~~~~~~~~~~~~~~~~~~~~~~~
aaa07c02.g1

                       3880      3890      3900      3910      3920      3930      3940      3950      3960
               ....|....|....|....|....|....|....|....|....|....|....|....|....|....|....|....|....|....|
rsmitogen      agaaattaaaatttaatataatagtaatataattgaagttttaaatattttaataatatatataatatattagtaattttaatattaatttt
rscox2
rstrna-his
rs16s
rsnad3
rstrna-leu
rscox3
rstrna-pro
rstrna-arg
rsnad6         agaaattaaaatttaatataatagtaatataattgaagttttaaatattttaataatatatataatatattagtaattttaatattaatttt
rsnad4L        ~~~~~~~~~~~~~~~~~~~~~~~~~~~~~~~~~~~~~~~~~~~~~~~~~~~~~~~~~~~~~~~~~~~~~~~~~~~~~~~~~~~~~~~~~~
rstrna-val     ~~~~~~~~~~~~~~~~~~~~~~~~~~~~~~~~~~~~~~~~~~~~~~~~~~~~~~~~~~~~~~~~~~~~~~~~~~~~~~~~~~~~~~~~~~
rstrna-leu     ~~~~~~~~~~~~~~~~~~~~~~~~~~~~~~~~~~~~~~~~~~~~~~~~~~~~~~~~~~~~~~~~~~~~~~~~~~~~~~~~~~~~~~~~~~
159494961oke
159496557oke
159497093oke
159496622oke
159498117oke
159494290oke
159495636oke
159497178oke
159498304oke
159496326oke
159495404oke
159493141oke
159493570oke
159493499oke
159493217oke
159493216oke
159493234oke
159492908oke
159491934oke
159492538
159498161
159493124
159493661
159494727
159496944      AGAAATTAAAATTTAATATAATAGTA
159494492      ~~~~~~~~~~~~~~~~~~~~~~~~~~~~~~~~~~~~~~~~~~~~~~~~~~~~~~~~~~~~~~~~~~~~~~~~~~~~~~~~~~~~~~~~~~
159496140      ~~~~~~~~~~~~~~~~~~~~~~~~~~~~~~~~~~~~~~~~~~~~~~~~~~~~~~~~~~~~~~~~~~~~~~~~~~~~~~~~~~~~~~~~~~
159497160      ~~~~~~~~~~~~~~~~~~~~~~~~~~~~~~~~~~~~~~~~~~~~~~~~~~~~~~~~~~~~~~~~~~~~~~~~~~~~~~~~~~~~~~~~~~
aaa07c02.g1

                       3970      3980      3990      4000      4010      4020      4030      4040      4050
               ....|....|....|....|....|....|....|....|....|....|....|....|....|....|....|....|....|....|
rsmitogen      taatgtgttatttttttattaattttttttttgataaatcaatatctatacgtattttttattatttttttattttttaggttattatttt
rscox2
rstrna-his
rs16s
rsnad3
rstrna-leu
rscox3
rstrna-pro
rstrna-arg
rsnad6         taatgtgttatttttttattaattttttttttgataaatcaatatctatacgtattttt
rsnad4L        ~~~~~~~~~~~~~~~~~~~~~~~~~~~~~~~~~~~~~~~~~~~~~~~~~~~~~~~~~~~attatttttttattttttaggttattatttt
rstrna-val     ~~~~~~~~~~~~~~~~~~~~~~~~~~~~~~~~~~~~~~~~~~~~~~~~~~~~~~~~~~~~~~~~~~~~~~~~~~~~~~~~~~~~~~~~~~
rstrna-leu     ~~~~~~~~~~~~~~~~~~~~~~~~~~~~~~~~~~~~~~~~~~~~~~~~~~~~~~~~~~~~~~~~~~~~~~~~~~~~~~~~~~~~~~~~~~
159494961oke
159496557oke
159497093oke
159496622oke
159498117oke
159494290oke
159495636oke
159497178oke
159498304oke
159496326oke
159495404oke
159493141oke
```

```
159493570oke
159493499oke
159493217oke
159493216oke
159493234oke
159492908oke
159491934oke
159492538
159498161
159493124
159493661
159494727
159496944
159494492      ~~~~~~~~~~~~~~~~~~~~~~~~~~~~~~TGATAAATCAATATCTATACGTATTTTTTATTATTTTTTTATTTTTTAGGTTATTATTTT
159496140      ~~~~~~~~~~~~~~~~~~~~~~~~~~~~~~~~~~~~~~~~~~~~~~~~~~~~~~~~~~~~~~~~~~~~~~~~~~~~~~~~~~~~~~~~~~
159497160      ~~~~~~~~~~~~~~~~~~~~~~~~~~~~~~~~~~~~~~~~~~~~~~~~~~~~~~~~~~~~~~~~~~~~~~~~~~~~~~~~~~~~~~~~~~
aaa07c02.g1

                       4060      4070      4080      4090      4100      4110      4120      4130      4140
               ....|....|....|....|....|....|....|....|....|....|....|....|....|....|....|....|....|....|
rsmitogen      tttttaaaaaaagaattaaaattatttaattaattttgtctttagagattttagtttttgttaatttattagtatttatatttttttaaa
rscox2
rstrna-his
rs16s
rsnad3
rstrna-leu
rscox3
rstrna-pro
rstrna-arg
rsnad6
rsnad4L        tttttaaaaaaagaattaaaattatttaattaattttgtctttagagattttagtttttgttaatttattagtatttatatttttttaaa
rstrna-val     ~~~~~~~~~~~~~~~~~~~~~~~~~~~~~~~~~~~~~~~~~~~~~~~~~~~~~~~~~~~~~~~~~~~~~~~~~~~~~~~~~~~~~~~~~~
rstrna-leu     ~~~~~~~~~~~~~~~~~~~~~~~~~~~~~~~~~~~~~~~~~~~~~~~~~~~~~~~~~~~~~~~~~~~~~~~~~~~~~~~~~~~~~~~~~~
159494961oke
159496557oke
159497093oke
159496622oke
159498117oke
159494290oke
159495636oke
159497178oke
159498304oke
159496326oke
159495404oke
159493141oke
159493570oke
159493499oke
159493217oke
159493216oke
159493234oke
159492908oke
159491934oke
159492538
159498161
159493124
159493661
159494727
159496944
159494492      TTTTTAAAAAAAGAATTAAAATTATTTAATTAATTTTGTCTTTAGAGATTTTAGTTTTTGTTAATTTATTAGTATTTATATTTTTTTAAA
159496140      ~~~~~~~~~~~~~~~~~~~~~~~~~~~~~~~AATTTTGTCTTTAGAGATTTTAGTTTTTGTTAATTTATTAGTATTTATATTTTTTTAAA
159497160      ~~~~~~~~~~~~~~~~~~~~~~~~~~~~~~~~~~~~~~~~~~~~~~~~~~~~~~~~~~~~~~~~~~~~~~~~~~~~~~~~~~~~~~~~~~
aaa07c02.g1

                       4150      4160      4170      4180      4190      4200      4210      4220      4230
               ....|....|....|....|....|....|....|....|....|....|....|....|....|....|....|....|....|....|
rsmitogen      atttaaattaaatatttatttttctttgtttatctgttatttctagagttattattatttattattattatttattattaaaaattaag
rscox2
rstrna-his
rs16s
rsnad3
rstrna-leu
rscox3
rstrna-pro
rstrna-arg
rsnad6
rsnad4L        atttaaattaaatatttatttttctttgtttatctgttatttctagagttattattatttattattattatttattattaaaaattaag
rstrna-val     ~~~~~~~~~~~~~~~~~~~~~~~~~~~~~~~~~~~~~~~~~~~~~~~~~~~~~~~~~~~~~~~~~~~~~~~~~~~~~~~~~~~~~~~~~~
rstrna-leu     ~~~~~~~~~~~~~~~~~~~~~~~~~~~~~~~~~~~~~~~~~~~~~~~~~~~~~~~~~~~~~~~~~~~~~~~~~~~~~~~~~~~~~~~~~~
159494961oke
159496557oke
159497093oke
159496622oke
159498117oke
159494290oke
159495636oke
159497178oke
159498304oke
159496326oke
159495404oke
159493141oke
159493570oke
```

```
159493499oke
159493217oke
159493216oke
159493234oke
159492908oke
159491934oke
159492538
159498161
159493124
159493661
159494727
159496944
159494492     ATTTAAATTAAATATTTATTTTTCTTTGTTTATCTGTTATTTCTAGAGTTATTATTATTTTATTATTATTATTTATTATTAAAAATTAAG
159496140     ATTTAAATTAAATATTTATTTTTCTTTGTTTATCTGTTATTTCTAGAGTTATTATTATTTTATTATTATTATTTATTATTAAAAATTAAG
159497160     ~~~~~~~~~~~~~~~~~~~~~~~~~~~~~~~~~~~~~~~~~~~~~~~~~~~~~~~~~~~~~~~~~~~~~~~~~~~~~~~~~~~~~~~~~~
aaa07c02.g1

                        4240      4250      4260      4270      4280      4290      4300      4310      4320
              ....|....|....|....|....|....|....|....|....|....|....|....|....|....|....|....|....|....|
rsmitogen     gtaatgataaattaattttatttattatttaggttatttaaagacctttaaaattactatttaaaagattaataattttcttataaagcg
rscox2
rstrna-his
rs16s
rsnad3
rstrna-leu
rscox3
rstrna-pro
rstrna-arg
rsnad6
rsnad4L       gtaatgataaattaattttat
rstrna-val    ~~~~~~~~~~~~~~~~~~~~~~tattatttaggttatttaaagacctttaaaattactatttaaaagattaataattt
rstrna-leu    ~~~~~~~~~~~~~~~~~~~~~~~~~~~~~~~~~~~~~~~~~~~~~~~~~~~~~~~~~~~~~~~~~~~~~~~~~~~~~~~~~~~~~~~~~~
159494961oke
159496557oke
159497093oke
159496622oke
159498117oke
159494290oke
159495636oke
159497178oke
159498304oke
159496326oke
159495404oke
159493141oke
159493570oke
159493499oke
159493217oke
159493216oke
159493234oke
159492908oke
159491934oke
159492538
159498161
159493124
159493661
159494727
159496944
159494492     GTAACGATAAATTAATTTTATTTATTATTTAGGTTATTTAAAGACCTTTAAAATTACTATTTAAAAGATTAATAATTTTCTTATAAAGCG
159496140     GTAATGATAAATTAATTTTATTTATTATTTAGGTTATTTAAAGACCTTTAAAATTACTATTTAAAAGATTAATAATTTTCTTATAAAGCG
159497160     ~~~~~~~~~~~~~~~~~~~~~~~~~~~~~~~~~~~~~~~AAAGACCTTTAAAATTACTATTTAAAAGATTAATAATTTTCTTATAAAGCG
aaa07c02.g1

                        4330      4340      4350      4360      4370      4380      4390      4400      4410
              ....|....|....|....|....|....|....|....|....|....|....|....|....|....|....|....|....|....|
rsmitogen     atttttaaatatattttacggatttggttaatttttattaatcctttttacgtaaaattattatatttaaaaatcaagataaactatttt
rscox2
rstrna-his
rs16s
rsnad3
rstrna-leu
rscox3
rstrna-pro
rstrna-arg
rsnad6
rsnad4L
rstrna-val
rstrna-leu    ~~~~~~~~~~~~~~~~~~~~~~~~~~~~~~~~~~~~~~~~~~~~~~~~~~~~~~~~~~~~~~~~~~~~~~~~~~~~~~~~~~~~~~~~~~
159494961oke
159496557oke
159497093oke
159496622oke
159498117oke
159494290oke
159495636oke
159497178oke
159498304oke
159496326oke
159495404oke
159493141oke
159493570oke
159493499oke
159493217oke
159493216oke
159493234oke
159492908oke
159491934oke
159492538
159498161
159493124
159493661
159494727
159496944
159494492     ATTTTTAAATATATTTTACGGATTTGGTTAATTTTTATTAATCCTTTTTACGTAAAATTATTATATTTAAAAATCAAGATAAACTATTTT
159496140     ATTTTTAAATATATTTTACGGATTTGGTTAATTTTTATTAATCCTTTTTACGTAAAATTATTATATTTAAAAATCAAGATAAACTATTTT
159497160     ATTTTTAAATATATTTTACGGATTTGGTTAATTTTTATTAATCCTTTTTACGTAAAATTATTATATTTAAAAATCAAGATAAACTATTTT
aaa07c02.g1

                        4420      4430      4440      4450      4460      4470      4480      4490      4500
              ....|....|....|....|....|....|....|....|....|....|....|....|....|....|....|....|....|....|
rsmitogen     ttaaaatagtttgtaataaatggcataaggaagcaatgaattataagaaaatttagttttatttcttttagaatattagtttttttacact
rscox2
rstrna-his
rs16s
rsnad3
rstrna-leu
rscox3
rstrna-pro
rstrna-arg
rsnad6
rsnad4L
rstrna-val
rstrna-leu    ~~~~~~~~~~~~~~~~~~~~~~~~~~~~~~~~~~~~~~~~~~~~~~~~~~~~ttagttttatttcttttagaatattagtttttttacact
159494961oke
159496557oke
159497093oke
159496622oke
159498117oke
159494290oke
159495636oke
159497178oke
159498304oke
159496326oke
159495404oke
159493141oke
159493570oke
159493499oke
159493217oke
159493216oke
159493234oke
159492908oke
159491934oke
159492538
159498161
159493124
159493661
159494727
159496944
159494492     TTAAAATAGTTTGTAATAAATGGCATAAGGAAGCAATGAATTATAAGAAAATTTAGTTTTATTTCTTTTAGAATATTAGTTTTTTTACACT
159496140     TTAAAATAGTTTGTAATAAATGGCATAAGGAAGCAATGAATTATAAGAAAATTTAGTTTTATTTCTTTTAGAATATTAGTTTTTTTACACT
159497160     TTAAAATAGTTTGTAATAAATGGCATAAGGAAGCAATGAATTATAAGAAAATTTAGTTTTATTTCTTTTAGAATATTAGTTTTTTTACACT
aaa07c02.g1

                        4510      4520      4530      4540      4550      4560      4570      4580      4590
              ....|....|....|....|....|....|....|....|....|....|....|....|....|....|....|....|....|....|
rsmitogen     aaaggttatatgactaaaaaaaataaaattattaataattttatatttttaaaaaaggttgaaatttttaagcacctattgaatttagta
rscox2
rstrna-his
rs16s
rsnad3
rstrna-leu
rscox3
rstrna-pro
rstrna-arg
rsnad6
rsnad4L
rstrna-val
rstrna-leu    aaaggttatatgactaaaaaa
159494961oke
159496557oke
159497093oke
159496622oke
159498117oke
159494290oke
159495636oke
159497178oke
159498304oke
159496326oke
159495404oke
159493141oke
159493570oke
159493499oke
159493217oke
```

```
159493216oke
159493234oke
159492908oke
159491934oke
159492538
159498161
159493124
159493661
159494727
159496944
159494492    AAAGGTTATATGACTAAAAAAAATAAAATTATTAATAATTTTATACCCGGCC
159496140    AAAGGTTATATGACTAAAAAAAATAAAATTATTAATAATTTTATATTTTTAAAAAAGGTTGAAATTTTTAAGCACCTATTGAATTTAGTA
159497160    AAAGGTTATATGACTAAAAAAAATAAAATTATTAATAATTTTATATTTTTAAAAAAGGTTGAAATTTTTAAGCACCTATTGAATTTAGTA
aaa07c02.g1

                     4600      4610      4620      4630      4640      4650      4660      4670      4680
             ....|....|....|....|....|....|....|....|....|....|....|....|....|....|....|....|....|....|
rsmitogen    ttattgatttttgttaataatttttattaataataggtttttttttt~gtttttttttttaagtattttttttttgtaaaattttttatag
rscox2
rstrna-his
rs16s
rsnad3
rstrna-leu
rscox3
rstrna-pro
rstrna-arg
rsnad6
rsnad4L
rstrna-val
rstrna-leu
159494961oke
159496557oke
159497093oke
159496622oke
159498117oke
159494290oke
159495636oke
159497178oke
159498304oke
159496326oke
159495404oke
159493141oke
159493570oke
159493499oke
159493217oke
159493216oke
159493234oke
159492908oke
159491934oke
159492538
159498161
159493124
159493661
159494727
159496944
159494492
159496140    TTATTGATTTTTGTTAATAATTTTTATTAACCCGGCC
159497160    TTATTGATTTTTGTTAATAATTTTTATTAATAATAGGTTTTTTTTTTTGTTTTTTTTTTTAAGTATTTTTTTTTTGTAAAATTTTTATAG
aaa07c02.g1

                     4690      4700      4710      4720      4730      4740      4750      4760      4770
             ....|....|....|....|....|....|....|....|....|....|....|....|....|....|....|....|....|....|
rsmitogen    tagtagtttaattttgtagctttttttgtttttggtttttttgttaagtattatttgtaaaaataaaaaatcttatataaaattatttaa
rscox2
rstrna-his
rs16s
rsnad3
rstrna-leu
rscox3
rstrna-pro
rstrna-arg
rsnad6
rsnad4L
rstrna-val
rstrna-leu
159494961oke
159496557oke
159497093oke
159496622oke
159498117oke
159494290oke
159495636oke
159497178oke
159498304oke
159496326oke
159495404oke
159493141oke
159493570oke
159493499oke
159493217oke
159493216oke
159493234oke
159492908oke
159491934oke
159492538
159498161
159493124
159493661
159494727
159496944
159494492
159496140
159497160    TAGTAGTTTAATTTTGTAGCTTTTTTTGTTTTTGGTTTTTTTGTTAAGTATTATTTGTAAAAATAAAAAATCTTATATAAAATTATTTAA
aaa07c02.g1

                     4780      4790      4800      4810      4820      4830      4840      4850      4860
             ....|....|....|....|....|....|....|....|....|....|....|....|....|....|....|....|....|....|
rsmitogen    aataaaaaatttttatttttaaaaaattaattttatatttttttaaataaaaaaatacaaaattaattataataaaaattaaaaataaaa
rscox2
rstrna-his
rs16s
rsnad3
rstrna-leu
rscox3
rstrna-pro
rstrna-arg
rsnad6
rsnad4L
rstrna-val
rstrna-leu
159494961oke
159496557oke
159497093oke
159496622oke
159498117oke
159494290oke
159495636oke
159497178oke
159498304oke
159496326oke
159495404oke
159493141oke
159493570oke
159493499oke
159493217oke
159493216oke
159493234oke
159492908oke
159491934oke
159492538
159498161
159493124
159493661
159494727
159496944
159494492
159496140
159497160    AATAAAAAATTTTTATTTTTAAAAAATTAATTTTATATTTTTTTAAATAAAAAAATACAAAATTAATTATAATACCCGGCC
aaa07c02.g1

                     4870      4880      4890      4900      4910      4920      4930      4940      4950
             ....|....|....|....|....|....|....|....|....|....|....|....|....|....|....|....|....|....|
rsmitogen    atttttttattttaaataattttagttttaaaaaaagatataaaatttttttacatacgtgtaaaatttttttatttatttataattaatttt
rscox2
rstrna-his
rs16s
rsnad3
rstrna-leu
rscox3
rstrna-pro
rstrna-arg
rsnad6
rsnad4L
rstrna-val
rstrna-leu
159494961oke
159496557oke
159497093oke
159496622oke
159498117oke
159494290oke
159495636oke
159497178oke
159498304oke
159496326oke
159495404oke
159493141oke
159493570oke
159493499oke
159493217oke
159493216oke
159493234oke
```

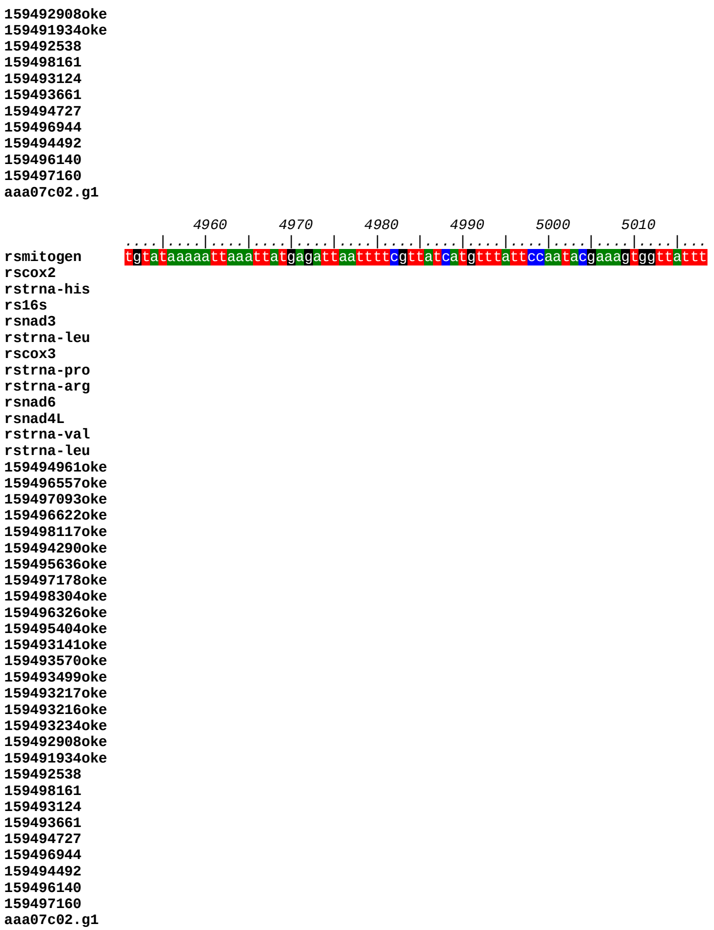

EST matching to repeat region 2

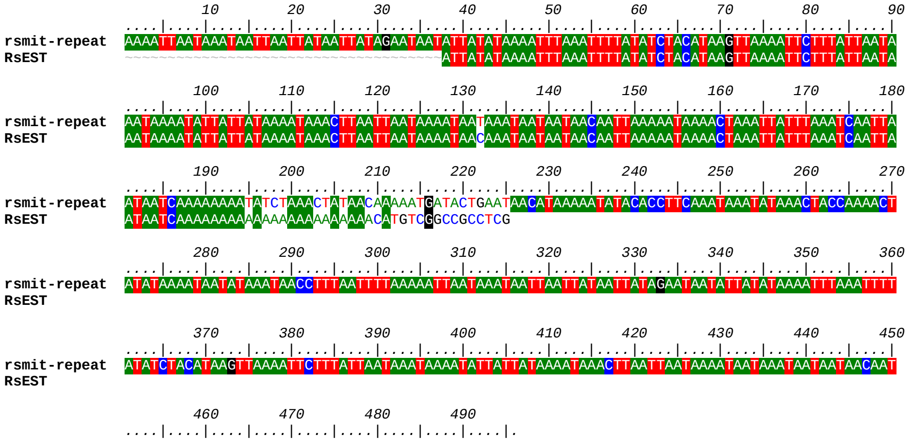

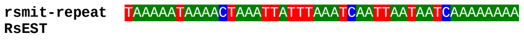

EST matching from *nad4* to *cytB*

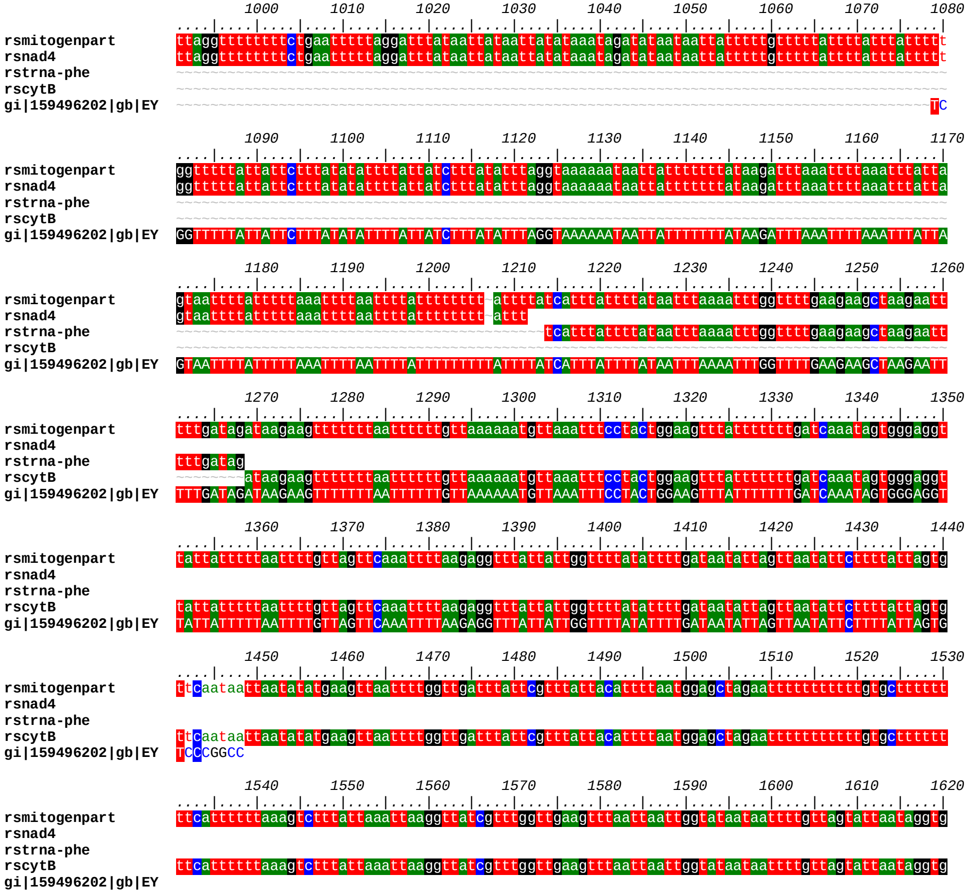

EST matching to *cox3*

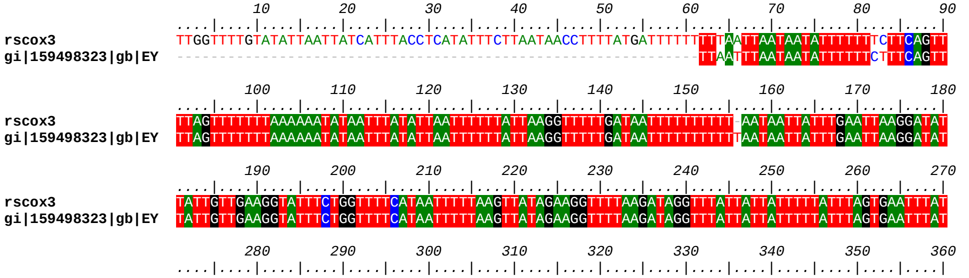

```
rscox3              ATTTTTTTTTAGTATTTTTTGATTTTTTTTT-GATAGATCTTTAGTCCCTTTAGGTGATTTTGGAGATAATTGAGTGCCTTTAGGTATAT
gi|159498323|gb|EY  ATTTTTTTTTAGTATTTTTTGATTTTTTTTTTGATAGATCTTTAGTCCCTTTAGGTGATATTGGAGATAATTGAGTGCCTTTAGGTATAT

                           370       380       390       400       410       420       430       440       450
                    ....|....|....|....|....|....|....|....|....|....|....|....|....|....|....|....|....|....|
rscox3              TATTAGTGAATCCTTTTGGTATACCATTTCTTAATTCTTTAATTTTATTAAGTAGAGCTGTAACTTTAACGTGATCACATTAAAGTTTAA
gi|159498323|gb|EY  TATTAGTGAATCCTTTTGGTATACCATTTCTTAATTCTTTAATTTTATTAAGTAGAGCTGTAACTTTAACGTGATCACATTAAAGTTTAA

                           460       470       480       490       500       510       520       530       540
                    ....|....|....|....|....|....|....|....|....|....|....|....|....|....|....|....|....|....|
rscox3              TTATAAATAATTCTTCATTAATAAGTTTATTTTAAACATTATTATTAGGTTTTTAATTTTTGTTAATTCAAATAATAGAGTAAAAAGAGT
gi|159498323|gb|EY  TTATAAATAATTCTTCATTAATAAGTTTATTTTAAACATTATTATTAGGTTTTTAATTTTTGTTAATTCAAATAATAGAGTAAAAAGAGT

                           550       560       570       580       590       600       610       620       630
                    ....|....|....|....|....|....|....|....|....|....|....|....|....|....|....|....|....|....|
rscox3              GTTTTTTTACTTTAAGTGATAGTGTTTTTGGTACTATTTTTTTTTTTGGTACTGGTTTTCATGGTTTACATGTTTTTTTGGGTTTATTAT
gi|159498323|gb|EY  GTTTTTTTACTTTAAGTGATAGTGCTTTTGGTACTATTTTTTTTTT-GGTACT-------------------------------------

                           640       650       660       670       680       690       700       710       720
                    ....|....|....|....|....|....|....|....|....|....|....|....|....|....|....|....|....|....|
rscox3              TTTTGTTATTTAATTATATTCGTATAAGATTATTTGAATTTTCTTCTATTCATCATTTAAGTTAAGAATTTTCTATTTTATAATGACATT
gi|159498323|gb|EY  ------------------------------------------------------------------------------------------

                           730       740       750       760       770
                    ....|....|....|....|....|....|....|....|....|....|....|.
rscox3              TTGTTGATGTTGTATGATTATTTTTGTATGTATTTTTTTATTAATGAAATTAATAG
gi|159498323|gb|EY  --------------------------------------------------------
```
